# Highly active fish in low oxygen environments: vertical movements and behavioural responses of bigeye and yellowfin tunas to oxygen minimum zones in the eastern Pacific Ocean

**DOI:** 10.1007/s00227-023-04366-2

**Published:** 2024-01-13

**Authors:** Nicolas E. Humphries, Daniel W. Fuller, Kurt M. Schaefer, David W. Sims

**Affiliations:** 1 The Laboratory, Marine Biological Association, Citadel Hill, Plymouth, PL1 2PB UK; 2 Inter-American Tropical Tuna Commission, La Jolla, San Diego, CA USA; 3 Ocean and Earth Science, University of Southampton, National Oceanography Centre Southampton, Waterfront Campus, Southampton, SO14 3ZH UK

**Keywords:** Anthropogenic impacts, Habitat compression, Marine predators, OMZ, Ocean deoxygenation

## Abstract

Oxygen minimum zones in the open ocean are predicted to significantly increase in volume over the coming decades as a result of anthropogenic climatic warming. The resulting reduction in dissolved oxygen (DO) in the pelagic realm is likely to have detrimental impacts on water-breathing organisms, particularly those with higher metabolic rates, such as billfish, tunas, and sharks. However, little is known about how free-living fish respond to low DO environments, and therefore, the effect increasing OMZs will have cannot be predicted reliably. Here, we compare the responses of two active predators (bigeye tuna *Thunnus obesus* and yellowfin tuna *Thunnus albacares*) to DO at depth throughout the eastern Pacific Ocean. Using time-series data from 267 tagged tunas (59,910 days) and 3D maps of modelled DO, we find that yellowfin tuna respond to low DO at depth by spending more time in shallower, more oxygenated waters. By contrast, bigeye tuna, which forage at deeper depths well below the thermocline, show fewer changes in their use of the water column. However, we find that bigeye tuna increased the frequency of brief upward vertical excursions they performed by four times when DO at depth was lower, but with no concomitant significant difference in temperature, suggesting that this behaviour is driven in part by the need to re-oxygenate following time spent in hypoxic waters. These findings suggest that increasing OMZs will impact the behaviour of these commercially important species, and it is therefore likely that other water-breathing predators with higher metabolic rates will face similar pressures. A more comprehensive understanding of the effect of shoaling OMZs on pelagic fish vertical habitat use, which may increase their vulnerability to surface fisheries, will be important to obtain if these effects are to be mitigated by future management actions.

**Supplementary Information:**

The online version contains supplementary material available at 10.1007/s00227-023-04366-2.

## Introduction

Anthropogenic climatic warming is driving increases in the volume and extent of oxygen minimum zones (OMZs), as well as increases in sea surface temperatures (SST), the combination of which is likely to threaten the functioning of marine ecosystems (Diaz and Rosenberg 2008; Stramma et al. 2008b; Keeling et al. 2010; Gruber 2011; Breitburg et al. 2018). Recovery of dissolved oxygen (DO) levels are predicted to be so long that the changes occurring at present are considered to be effectively irreversible in human timescales (Gruber 2011). Open-ocean OMZs have increased in extent over the last 60 years, with a quadrupling in the volume of anoxic water (Schmidtko et al. 2017; Breitburg et al. 2018). The northern and equatorial Pacific Ocean has seen the largest reductions in DO over the last 50 years, with approximately 40% of the global loss of oxygen occurring in this region (Schmidtko et al. 2017). Higher sea temperatures have increased stratification and reduced oxygen solubility which, with the upwelling of low DO water, have resulted in these increases (Levin 2018). Increased primary productivity in surface layers has raised the quantity of organic matter available for sub-surface microbial respiration, which further depletes mesopelagic DO concentrations (Diaz and Rosenberg 2008; Breitburg et al. 2018). Consequently, in the eastern equatorial regions of the Atlantic and Pacific Oceans, there are extensive OMZs that exist within the depth range of 100–900 m that overlap both horizontally and vertically with marine predator hotspots (Karstensen et al. 2008; Czeschel et al. 2012; Queiroz et al. 2016, 2019; Olivar et al. 2017; Vedor et al. 2021b). These OMZs are likely to increase in volume further, with reductions in global dissolved oxygen of up to 7% predicted by 2100 (Schmidtko et al. 2017) with potentially serious consequences for marine life (Stramma et al. 2008b). Hypoxia has been shown to have far-reaching effects not only on a wide range of taxa, but also to be consistently detrimental to almost all biological processes, such as survival, growth, development, and reproduction (Sampaio et al. 2021). While mitigation of these problems through the reduction in CO_2_ emissions is therefore essential, it is also vital to gain an understanding of how expanding OMZs will impact marine ecosystems if measures are to be implemented to avoid or reduce these effects.

Two processes combine to reduce the volume of suitable habitat above OMZs. First, as the OMZ expands, it forces deoxygenated water into shallower layers of the water column, thus reducing the volume from below. Simultaneously, rising sea surface temperatures reduce the volume of preferred habitat from above. This compression reduces the vertical extent of habitable water column for fishes and other organisms above the mesopelagic OMZ (Prince and Goodyear 2006; Prince et al. 2010; Stramma et al. 2010, 2011; Vedor et al. 2021b). Consequently, as the depth at which organisms experience hypoxia shoals, those with higher oxygen demands will be forced into a narrowing volume of cooler, better oxygenated water. Fishes may become habitat compressed and therefore become more vulnerable to fishing effort from surface longlines for example (Vedor et al. 2021b). Alternatively, fish might be displaced horizontally to areas outside the volume occupied by the OMZ if the hypoxic tolerance of prey species, such as euphausiids, myctophids, or squid (Trubenbach et al. 2013; Seibel et al. 2016; Olivar et al. 2017), is greater than that of the predators (e.g., tunas or sharks) which may affect foraging opportunities (Vetter et al. 2008; Breitburg et al. 2018).

Highly active, water-breathing marine predators, such as tunas, billfish, and sharks with high oxygen requirements, are likely to be most affected by changes in the distribution of low DO in the oceans (Brill 2007; Gilly et al. 2013). Consequently, bigeye tuna (*Thunnus obesus*; hereafter BET) and yellowfin tuna (*Thunnus albacares*; hereafter YFT) are appropriate species to test for the effect of low DO as they have relatively high metabolic rates among fish with consequently high O_2_ demands and are therefore likely to be more sensitive to low DO (Bernal et al. 2017). Istiophorid billfish, for example, are known to exhibit very different vertical movements with respect to DO, foraging in shallower water in the eastern tropical Pacific which is characterised by low DO at depth, and foraging in deeper waters in the western tropical Atlantic where DO is higher (Prince and Goodyear 2006). Furthermore, both tuna species are commercially important, together making up about 35% of the total global tuna catch (ISSF 2020). Therefore, understanding the importance of expanding OMZs to these species has implications not only for marine ecology but also for tuna fisheries management and food security (Baez et al. 2018).

The BET and YFT studied here exhibit vertical movements that result in different distributions of time-at-depth. Both species typically perform normal diel vertical migration (nDVM), moving to deeper water to forage during the day, with BET diving to around 200–300 m and YFT to around 50–150 m (Schaefer et al. 2009; Schaefer and Fuller 2010). Consequently, both species are likely to encounter low DO water which might act as a physiological or behavioural boundary to their vertical movements and foraging opportunities. There are considerable differences between BET and YFT, not only in the vertical habitat occupied, but in their physiological adaptations to both temperature and oxygen (Bernal et al. 2017). Given that BET spend the majority of the daytime in low DO waters when foraging at depth (Leung et al. 2019), well below the commonly accepted hypoxic threshold of 63 μmol/l (Breitburg et al. 2018), it is expected that BET would have adaptations specific to low DO and lower temperatures at depth (Bernal et al. 2017). Indeed, BET have better control over the thermoregulation of red muscle tissue by being able to selectively route blood through two vascular heat exchangers, varying the extent to which metabolic heat is retained in swimming muscles (Bernal et al. 2017). The heart muscle of BET has also been shown to have higher tolerance of low temperatures than that of YFT (Bernal et al. 2017). These adaptations provide greater tolerance to low temperatures, allowing BET to forage during the day well below the thermocline in water as cold as 7 °C. By contrast, YFT are generally restricted to warmer waters above the thermocline (Bernal et al. 2010). Furthermore, BET have been shown to have a higher blood–oxygen-binding affinity than YFT or skipjack tunas (*Katsuwonus pelamis*) (Lowe et al. 2000; Bernal et al. 2017). While studies using archival tag data confirm that YFT spend less time in hypoxic waters, they do not show the expected behavioural responses to low DO (such as increased swimming speed to improve ventilation) until DO reaches levels as low as 75 µmol/l and are able to survive hypoxic conditions for over 3 h (Bernal et al. 2017). These findings suggest that responses to low DO by BET and YFT are variable and depend on thermal habitat or prey distributions as well as hypoxia for determining times spent above and below the thermocline. For example, it is known that BET and YFT favour different forage taxa, with BET consuming more fish and squid and YFT having a greater preference for crustaceans (Menard et al. 2006) and this differing prey preference could also contribute to the observed differences in water column occupancy.

Many studies have identified distinct vertical habitats for BET and YFT, and laboratory studies have revealed tolerances for temperature and DO (for reviews see Bernal et al. 2010; Leung et al. 2019). However, it is poorly understood to what extent actual DO levels in the open ocean may affect the vertical movements and behaviour of populations of BET or YFT, such that a representative overview of responses may be obtained. Although it is expected that low DO will affect the time spent at depth by each species, a comprehensive analysis of vertical movements in response to DO will help inform the effects expanding OMZs will have on these tunas and how this may later affect foraging opportunities and the vulnerability to surface fisheries.

In this study, we investigate the extent to which dissolved oxygen levels at a range of depths may constrain the vertical space use of bigeye and yellowfin tuna. We do so by identifying changes in the occupancy of the water column from tuna electronically tagged in throughout the eastern Pacific Ocean (EPO). Clearly, experimental modification of DO profiles in the ocean is not possible, but we are able to analyse vertical movements and behavioural responses of the tuna in terms of the extent of the water column used in contrasting areas of high and low DO at depth. We hypothesise that YFT will respond more markedly to low DO than BET and that occupancy of the water column by YFT, but not BET, will be shallower where DO at depth is lower. To test this, our approach was to first determine threshold depths at which vertical occupancy changed in relation to low DO at foraging depths. Having identified thresholds for each species, we then used these to select areas (1-degree grid cells) where vertical occupancy of the water column differed and analyse DO and temperature at a range of depths to determine the likely drivers for the differing occupancy.

Additionally, BET are known to perform periodic vertical ascents to shallower waters while foraging at depth. These ascents (upward vertical excursions) are thought to allow rewarming after time spent in deeper, colder water (Schaefer et al. 2009; Schaefer and Fuller 2010). However, we also hypothesise here that these ascents into water with higher DO concentrations may enable faster physiological recovery from time spent in low DO waters. Therefore, we analysed vertical excursions in relation to DO at foraging depths, to test the hypothesis that the number of vertical ascents will be greater where DO at depth was lower. Finally, both species have been observed in numerous studies to perform occasional very deep dives (e.g., Schaefer et al. 2007; Schaefer and Fuller 2010; Fuller et al. 2015). Typically, these take the form of bounce dives (where little time is spent at depth) and reach depths of over 1800 m for BET and 1600 m for YFT. The purpose of these dives in tuna and many other species, for example blue sharks *Prionace glauca* and whale sharks *Rhincodon typus* (e.g., Brunnschweiler et al. 2009; Queiroz et al. 2012) is yet to be determined. We therefore analysed the occurrence of these dives in relation to DO at depth, to investigate whether DO concentrations at depth drive or inhibit these events.

## Methods

### Summary

This study used high-resolution depth time-series data from 92 BET and 175 YFT tagged in the Eastern Pacific Ocean (EPO) in 2000 and between 2003 and 2005 (BET) and 2002 and 2011 (YFT). Light-based geolocation estimates were modelled with the unscented Kalman filter (uKFSST) which incorporates remotely sensed SST fields to derive most probable daily locations (Lam et al. 2010). Dissolved oxygen (DO) throughout the water column within 1-degree grid cells was determined from modelled DO datasets (Copernicus Marine Services, CMEMS, https://www.copernicus.eu). From time-at-depth (TAD) profiles, time activity profiles, and prior research (Schaefer and Fuller 2002, 2010; Musyl et al. 2003; Schaefer et al. 2007; Matsumoto et al. 2013), it was clear that both species are active at depth during daylight hours and, therefore, it is over these times that the interaction with DO was probably occurring. Consequently, nighttime activity was excluded from the analysis, and from the daytime time-at-depth profiles, the depths at which most time was spent were determined for both species as 300 m for BET and 100 m for YFT.

Depth time-series data and light level geolocation positions were merged for 92 BET and 175 YFT tracks, to produce a 3D track for each individual with the resulting data being loaded into an SQL Server data base for subsequent analysis. To first identify the overall response (in terms of the TAD profiles) of the fish to different levels of DO at depth, a 1-degree grid was defined over the study area, and in each occupied grid cell, the mean DO at the putative foraging depths of 300 m for BET and 100 m for YFT was determined. Using these DO levels, the grid cells were separated into the upper and lower 10th quantiles, to represent two extremes of DO concentration potentially encountered by tagged tunas. By comparing time-at-depth plots produced from within these two sets of grid cells, differences in vertical habitat use between high and low DO areas were determined. In both species, depth thresholds were identified where occupancy of specific depths differed in low DO areas and, thus, a behavioural response to low DO was detected. Because the thresholds at which a behavioural response was identified differed markedly from the depth at which DO concentrations were used to separate grid cells, we then used the response threshold depths to further investigate the response to DO and temperature at a range of depths. To do so, we identified areas (sets of 1-degree grid cells) where the tuna spent more time above or below these response thresholds, separating them again into the upper and lower 10th percentiles. We then analysed depth time-series locations within these areas to compute correlation coefficients between the time spent below the response threshold depths and DO concentrations and temperature. By doing so, we were able to relate occupancy of the water column with DO and temperature at a range of depths. These relationships were further investigated using Generalised Additive Models (GAMs). For BET, we also analysed the number of vertical excursions from depth to surface waters the tuna performed in relation to DO and temperature, to test the hypothesis that DO, as well as temperature, was a driver for these characteristic movements. Finally, we investigated the occurrence of exceptional deep dives in reference to high and low DO grid cells.

### Tagging

Full details regarding the materials and methods utilised in the capture, tagging, and release of the fish are given by Schaefer and Fuller et al. (2002, 2007, 2009, 2010). BET tuna were captured, tagged, and released while associated with both drifting fish aggregating devices (dFADs) and moored oceanographic buoys in the equatorial EPO between 02°12 S and 2°00′N and between 94°42′ and 95°29′W, during 15–22 April 2000, March–May 2003, 2004, and 2005. Tagging was conducted on the chartered FV *Her Grace*, a 17.7-m, 99 gross-t, United States west-coast-style live-bait pole-and-line vessel. YFT were captured, tagged, and released along the coast of Baja California, Mexico between 23°18 N and 31°45′N and between 110°20′ and 118°24′W during November 2002–November 2008, and areas surrounding the Revillagigedo Islands, Mexico between 18°19 N and 19°20′N and between 110°54′ and 114°45′W during February 2006–May 2011. Tagging was conducted aboard the US flagged passenger carrying fishing vessels FV *Royal Star* and FV *Shogun*, both home-ported in San Diego, California. The archival tags used were model Mk7 and MK9 manufactured by Wildlife Computers (Redmond, WA) (Wildlife Computers, 2002), LTD_2310 and LTD_2350 manufactured by Lotek Wireless, Inc., St. John’s, Newfoundland, Canada (Schaefer and Fuller 2016). The total weight of the tags in air is about 32–40 g. Tags were designed for implantation into the peritoneal cavity of the fish, so that the sensor stalk protrudes outside the fish through an incision in the abdominal wall. A label, printed in Spanish, with information about reporting the recovery of the tag and the associated reward (US$250) was encased in the epoxy of the main body of the instrument.

### Preparation of data

For BET, 92 geo-located tracks with daily position locations were available for analysis; the light level geolocation and modelling process is described fully in Schaefer and Fuller (2009). For YFT, 175 geo-located tracks were available giving a total of 267 tracks in this study. Details of these tracks are given in the Supplementary information, Tables S1 and S2.

Dive time-series were recorded with sampling intervals ranged from 4 s to 4 min, so to standardise the data, all tracks were interpolated to 4-min intervals. This standardisation was verified in a sensitivity test (Supplementary and Figure S1). The daily position locations and the dive time-series data were then merged by interpolating the daily position locations linearly at 4-min intervals, to match the times in the depth time-series. The time of each location (originally recorded in UTC) was then converted to local time using the estimated longitude, with 15 degrees of longitude corresponding to a difference of 1 h. To remove from the analysis those days where the fish were known to be associating with floating Fish Aggregation Devices (FADs) and also to remove any post-tagging behavioural anomalies, we removed the first 14 days of data from every track. Prior analysis of the BET tracking data reveals days throughout the tracks where the tuna exhibit what is referred to as ‘surface-oriented’ behaviour and it is possible that on those days, the fish were associated with FADs. However, as these behaviours occurred when the fish were beyond observation, FAD association could not be objectively confirmed as the sole cause of the occupancy of shallower waters and, therefore, it was concluded that these days should not be removed from the analysis. To confirm that this choice did not unduly affect the work, a sensitivity analysis was performed (see Supplementary Analysis and Figures S2, S3, S4 and S5) that showed no significant differences. Consequently, the more conservative route of retaining these days was taken.

Following the merge of the position and depth data, additional environmental data were collated, so that each datum comprised track name (ID), date, latitude, longitude, depth, temperature, DO, bathymetric depth, and mixed layer depth. All data were written to an SQL Server database to allow selection of data for later analysis. Temperature data were recorded by the tag along with depth; DO and mixed layer depth were obtained from 3D statistical models from Copernicus Marine Services (CMEMS, https://marine.copernicus.eu/); bathymetry was obtained from the Gebco 30 s product (GEBCO_2014 Grid, version 20,150,318, www.gebco.net). All the analysis performed in this study began by selecting the required sub-set of data from the database (e.g., all locations from selected 1-degree grid cells) into CSV files that were imported into Excel (Microsoft Corporation) or SigmaPlot (Systat Software, San Jose, CA) for further analysis.

### Determining changes in vertical habitat use in response to low DO

For this preliminary analysis, daytime Time at Depth (TAD) plots (i.e., between the local times of 06:00 and 17:00), together with example dive time-series plots showing depth, temperature, and DO, were used as a guide to the depth at which DO was likely to be important for each species. It was hypothesised that the lower bound of the daytime depth where DO will be lower is more likely to represent a possible boundary at which the DO concentration could influence behaviour; consequently, 300 m was selected as a depth towards the lower edge of the vertical activity range of BET, while, for YFT, a value of 100 m was selected. DO concentrations at these depths (from the mean 2005 dataset) were then determined for all occupied 1-degree grid cells, and using these values, grid cells in the 10th and 90th percentiles were selected to represent the two extremes of modelled DO ‘encountered’. For BET, there were 336 occupied grid cells, from which we selected 33 locations for the upper and lower percentiles; for YFT, there were 443, giving 44 locations for each. The geographic ranges of these cells were used to select locations from the depth time-series data and generate time-at-depth plots corresponding to high and low DO locations. From these TAD plots, we identified depth thresholds for BET and YFT at which there were significant differences in occupancy of shallower waters between the low and high DO locations.

### Determining the response to DO and temperature from behavioural depth thresholds

The behavioural depth thresholds (see previous section) were then used to identify areas where DO at a range of depths (i.e., at depths other than the specified depth used previously) might be affecting behaviour. We therefore analysed all occupied 1-degree grid cells in the study area by comparing DO concentrations and temperatures at a range of depths between locations where most time was spent above or below the behavioural depth thresholds for each species. To do so, all daytime depth records (i.e., between 06:00 and 17:00 local time) were extracted for each occupied grid cell and the times above and below the threshold depths previously identified were calculated. Using these proportions of time, the grid cells in the upper and lower 10th percentiles were selected. From these grid cells, differences between the upper and lower 10th percentiles of grid cells were computed for DO and temperature at depths of 50, 100, 150, 200, 250, and 300 m together with the median and maximum daily dive depths and the bathymetric depths. To test the hypothesis that DO at depth was a driver for the observed change in the distribution of time-at-depth, correlation coefficients (Pearson’s *r*) were computed between the proportion of time spent below the threshold and the DO concentrations, temperatures, mixed layer depth, median depth, and bathymetric depth for all grid cells. As the response to DO might not be linear, we also developed Generalised Additive Models (GAMs) to investigate the relationships between time spent below the depth threshold and DO and temperature at a range of depths as described in more detail below.

### Analysis of BET vertical excursions

Vertical excursions in BET, from depth, to warmer surface waters are well known and have been observed in many studies. Vertical excursions have been hypothesised to allow the animal to rewarm after spending usually around an hour in colder deep water (Schaefer and Fuller 2002; Musyl et al. 2003). To investigate the extent to which low DO at depth might be a driver for vertical excursions, the number of upward vertical excursions performed between the local times of 06:00 and 17:00 in the one-degree cells with the lowest and highest 10th percentiles of DO were compared. The analysis was extended to compare the number of vertical excursions between the 10th–90th, 20–80, 30–70, and 40–60 percentiles, to identify the level to which DO might be a driver for vertical excursions.

### Analysis of the occurrence of exceptional deep dives

To investigate whether the frequency and depth of exceptional deep dives was influenced by low DO, we compared deep dives that occurred in the 10th and 90th percentile grid cells for DO at 300 or 100 m for BET and YFT, respectively. We selected dives deeper than 500 m for BET and deeper than 250 m for YFT.

### GAM analysis

A range of Generalised Additive Models (GAMs) were developed, using the R mgcv package (Wood 2011), to investigate non-linear relationships in more detail. Response variables (e.g., time-at-depth, median depth, and maximum depth) were computed from all locations within each occupied 1-degree grid square. Because the chosen response variables were not necessarily normally distributed, the first step determined the appropriate distribution for each model (e.g., Gaussian, Log Gaussian, and Gamma). Second, the environmental variables being considered exhibit strong collinearity, or more properly for GAMs, concurvity (Gu et al. 2010), with, for example, DO at 300 m being strongly related to DO at 250 m. To reduce this problem in the models, a preliminary analysis was performed to determine the most significant depth for DO and temperature for each response variable, with subsequent compound models using these single values (e.g., Temperature at 50 m and DO at 200 m). While SST likely does exhibit concurvity with shallow temperatures and DO concentrations, SST has been used as an indicator of water column conditions (Vedor et al. 2021a) and was therefore included. For all response variables a number of uni- and multi-variate models were developed.

To complement the large-scale spatial analysis described above, GAMs were used to analyse responses to DO and temperature from individual time-series data. Daytime (local time 06:00 to 17:00) locations were selected from the time-series data to provide date, depth, latitude, and longitude which were used to provide, for each day and individual, the maximum daily dive depth, average dive depth and, for BET, a count of the number of vertical excursions performed. Because the occasional very deep dives performed by both species were considered to be outside of normal vertical occupancy, dive locations with depths > 500 m were excluded from the analysis. This could not be done for the spatial analysis, as nearly all grid cells had maximum depths > 500 m. For each individual day, the location was used to derive DO and temperature at depths of 50, 100, 150, 200, 250, and 300 m as well as sea surface temperature (SST), to provide the environmental variables for the modelling. To allow for the error fields inherent in the light level geo-located positions, environmental variables were computed as a mean value from a 1-degree area centred on the location. The unique track identification number was included in preliminary models to assess the effect of individual variation, as was body (fork) length. Thus, the GAM analysis was performed using both spatial (large scale) and individual (detailed) data.

## Results

### Geographic distribution of tagging data

**Fig. 1:**
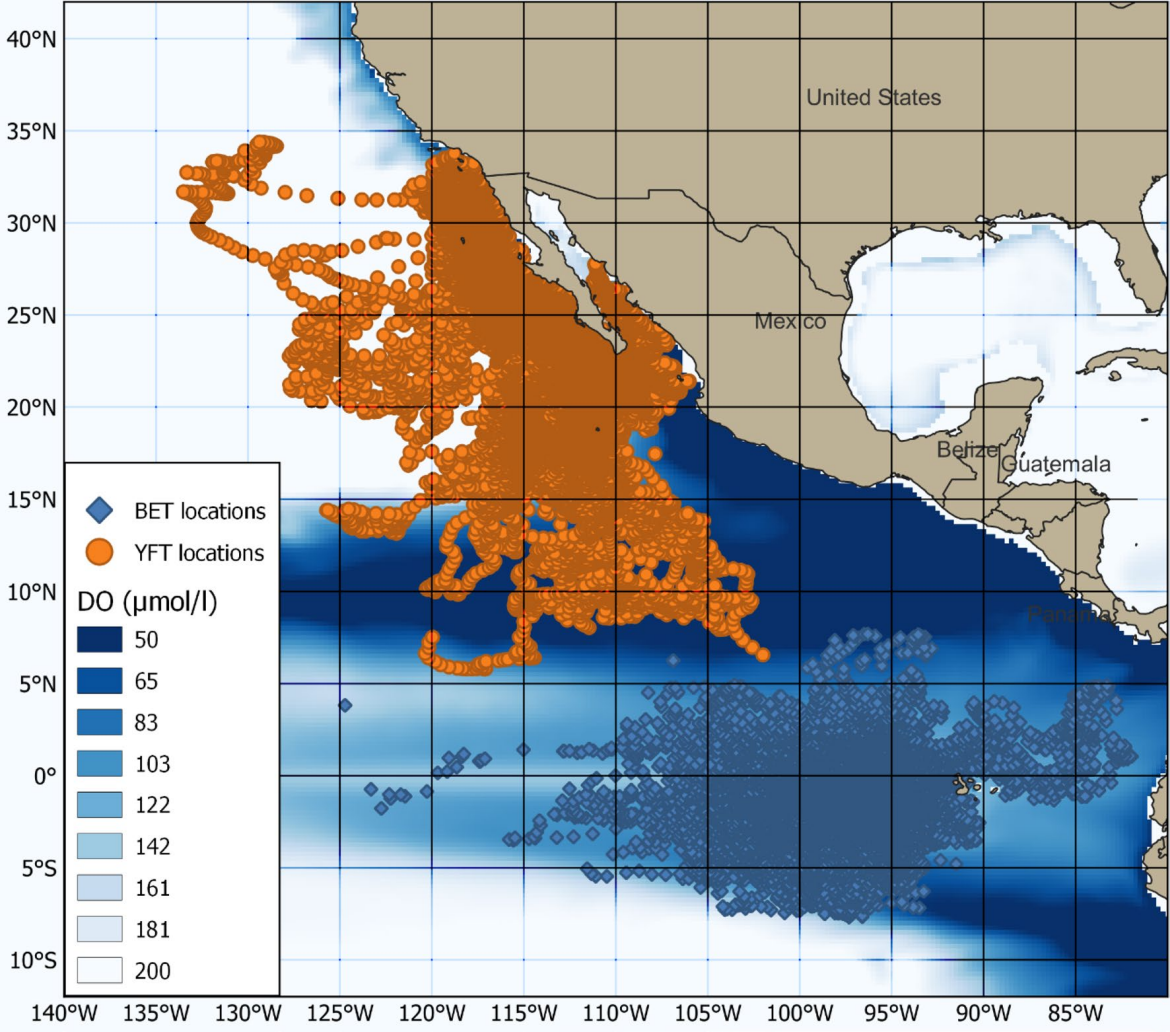
Distribution of depth time-series locations in the study area. Orange is YFT, blue is BET, and DO is the average for 2005 (µmol/l) at 100 m. A 5-degree grid is superimposed

The most probable tracks by individuals of the two species occupied two distinct areas of the EPO. The tracks of both species extended over large areas and a broad range of depths, although it was evident that some YFT exhibited a more coastal distribution around Baja California (Fig. 1).

### Behavioural thresholds for DO response analysis

**Fig. 2:**
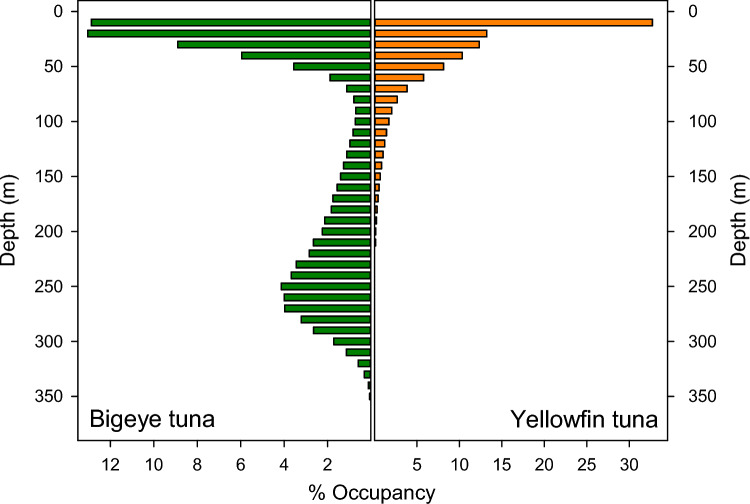
TAD for daylight hours of 06:00–17:00 local time. BET spend significantly more than YFT time at depths where DO reaches hypoxic levels (< ~ 63 µmol/L (Breitburg et al. 2018)

**Fig. 3:**
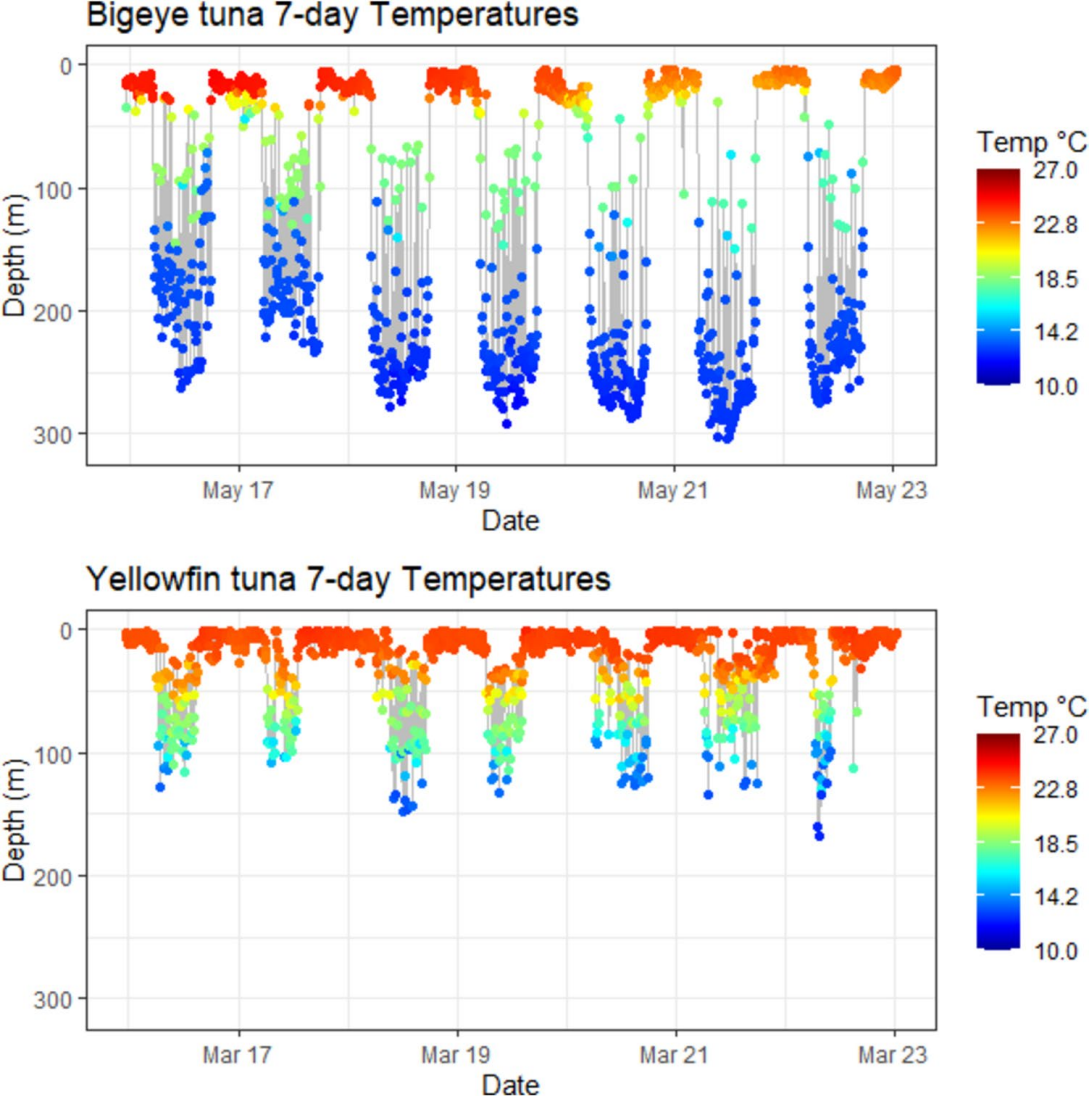
Tuna depth time-series showing water temperatures experienced. Plots show 7 day depth time-series for BET 1102 2003 and YFT 0490916 with a heat map of temperatures as recorded by the tags. BET spent more time in deeper colder water, while YFT range continuously between 25 and 100 m with occasional dives to 100 m

**Fig. 4:**
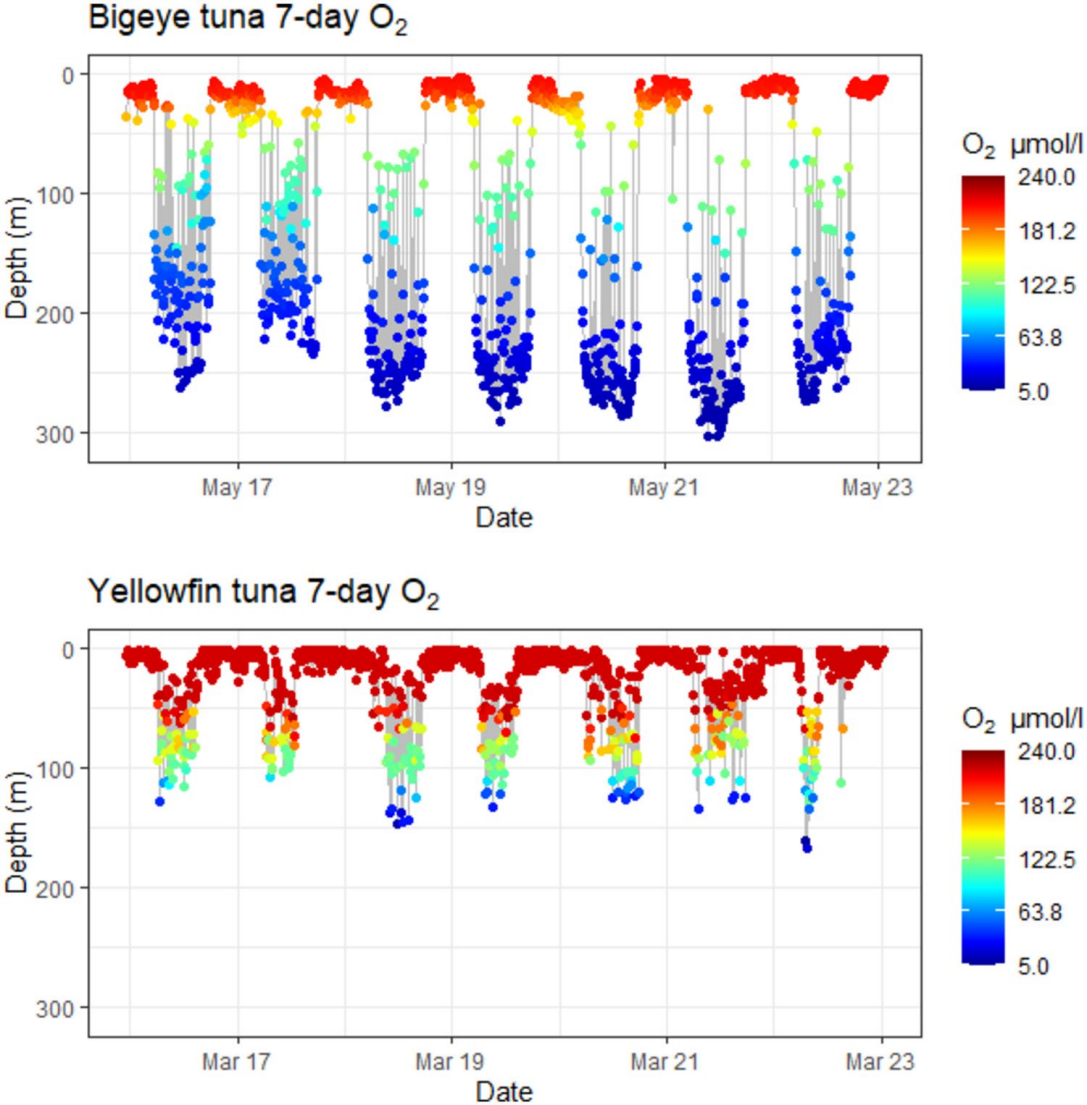
Tuna depth time-series showing O_2_ levels experienced. Plots show 7 day depth time-series for BET 1102 2003 and YFT 0490916 with a heat map of O_2_ computed from the modelled O_2_. Although BET spent more time-at-depth, YFT experience equally low levels of O_2_

**Fig. 5:**
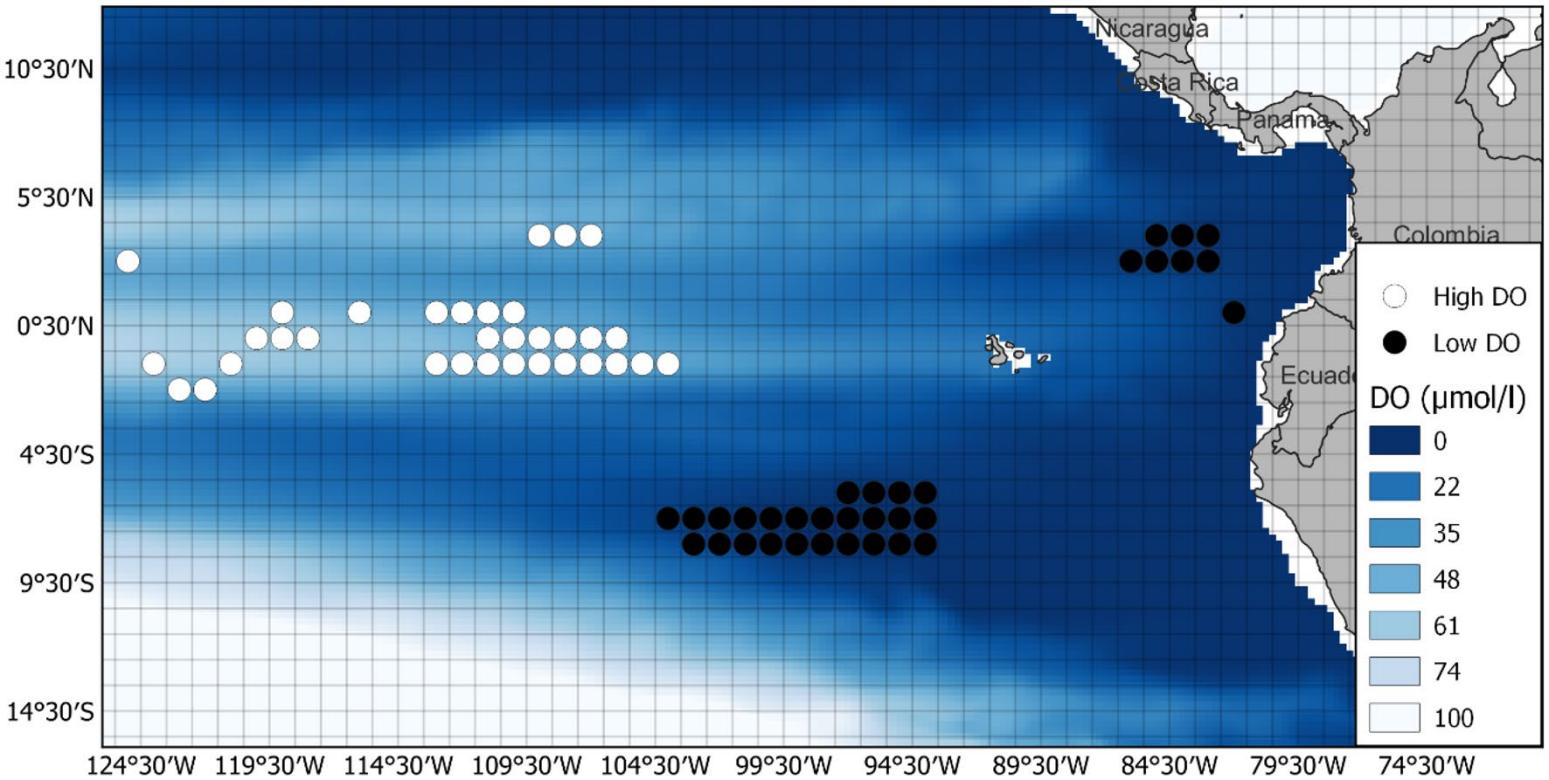
Grid cells selected for the BET analysis. The 1-degree grid cells selected for high DO (white) and low DO (black) analysis. DO heat map is the annual mean at 300 m for 2005. Legend shows DO in μmol/l

**Fig. 6:**
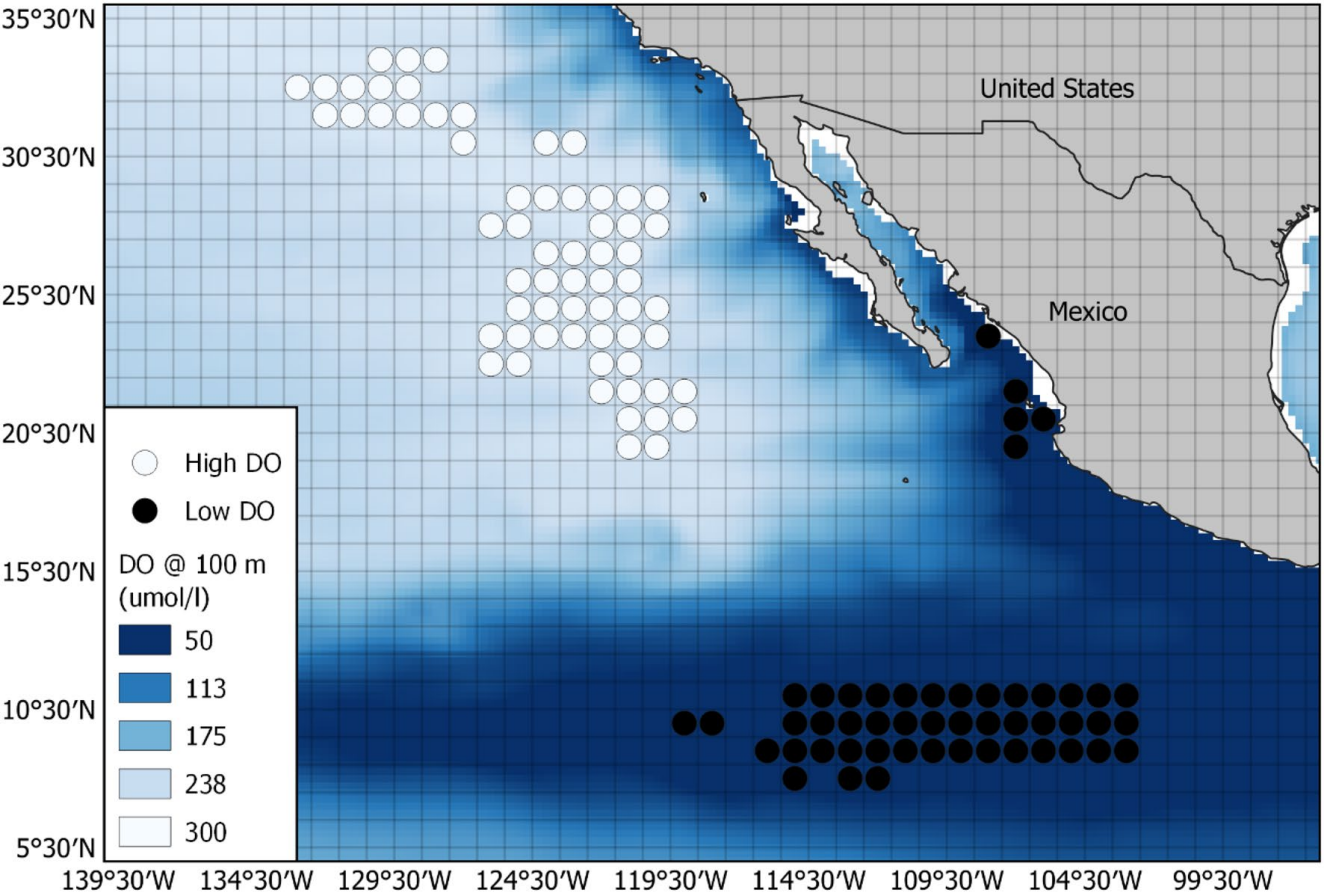
Grid cells selected for the YFT analysis. The 1-degree grid cells selected for high DO (white) and low DO (black) analysis. DO heat map is the annual mean at 100 m for 2005. Legend shows DO in μmol/l

We determined depths at which low DO might affect behaviour as being 300 m for BET and 100 m for YFT (Figs. 2, 3, 4). Using these values, we then selected grid cells where modelled DO at these depths was in the upper or lower 10th percentile (Figs. 5 and 6).

### Changes in vertical habitat use in response to low DO

**Fig. 7:**
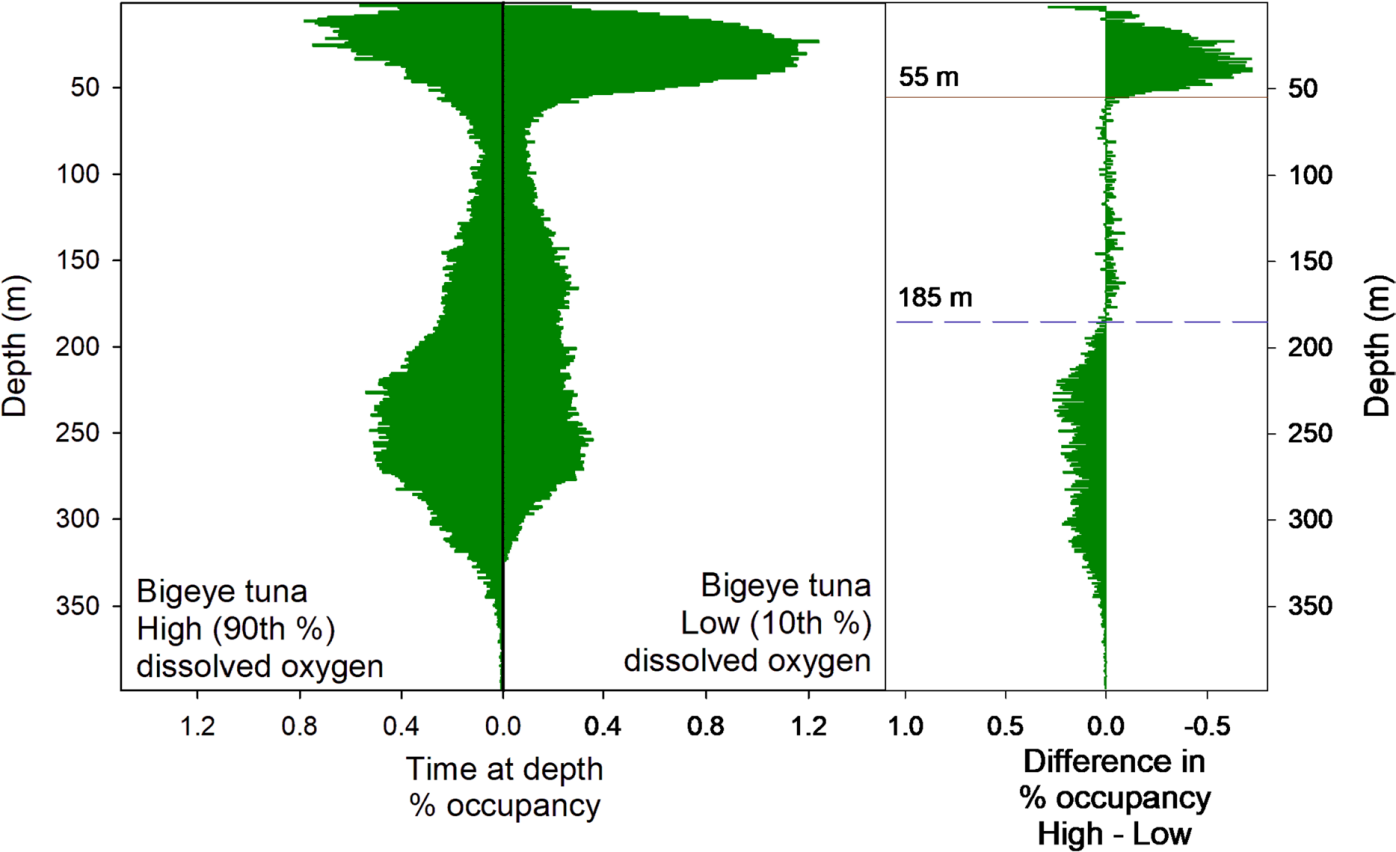
BET Time at depth for high and low DO areas. High DO areas comprise those 1 × 1 degree grid cells where DO at 300 m is above the 90th percentile. Low DO areas are the grid cells below the 10th percentile. There is a discontinuity in TAD at 55 m, below which BET spend less time in low DO areas; by contrast, in high DO areas, BET spend less time above 55 m. There is a further discontinuity at 185 m, below which BET spend more time in high DO areas

**Fig. 8:**
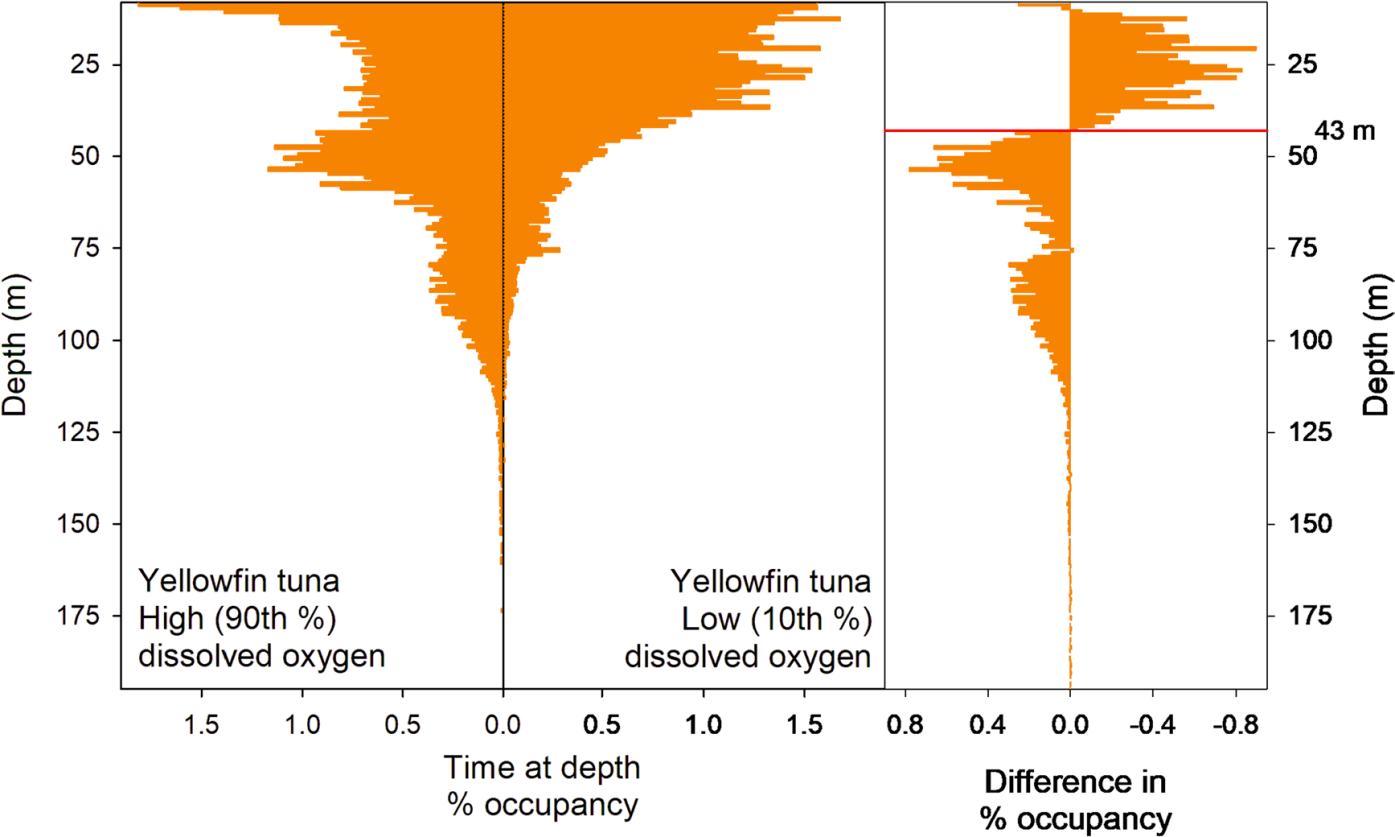
YFT Time at depth for high and low DO areas. High DO areas comprise those 1 × 1 degree grid cells where DO at 100 m is above the 90th percentile. Low DO areas are the grid cells below the 10th percentile. There is a clear discontinuity in TAD at 45 m, below which YFT spend less in low DO areas; by contrast, in high DO areas, YFT spend less above 45 m. YFT spend around 12% of their time at 1 m; this depth has been omitted for clarity

The resulting TAD plots identified discontinuities in the time-at-depth between the high DO and low DO areas (Figs. 7 and 8). For BET, 7.8% more time is spent above 20 m when DO is high (*p* < 0.001, signed-rank test), 12.6% more is spent between 20 and 55 m when DO is low (*p* < 0.001, signed-rank test), and 5.3% more time is spent below 55 m when DO is higher (*p* < 0.001, signed-rank test). YFT spend 16.8% more time-at-depth below 43 m when DO is higher (*p* = 0.012, signed-rank test) and 14% more time between 10 and 43 m when DO is low (*p* < 0.001, signed-rank test).

**Fig. 9:**
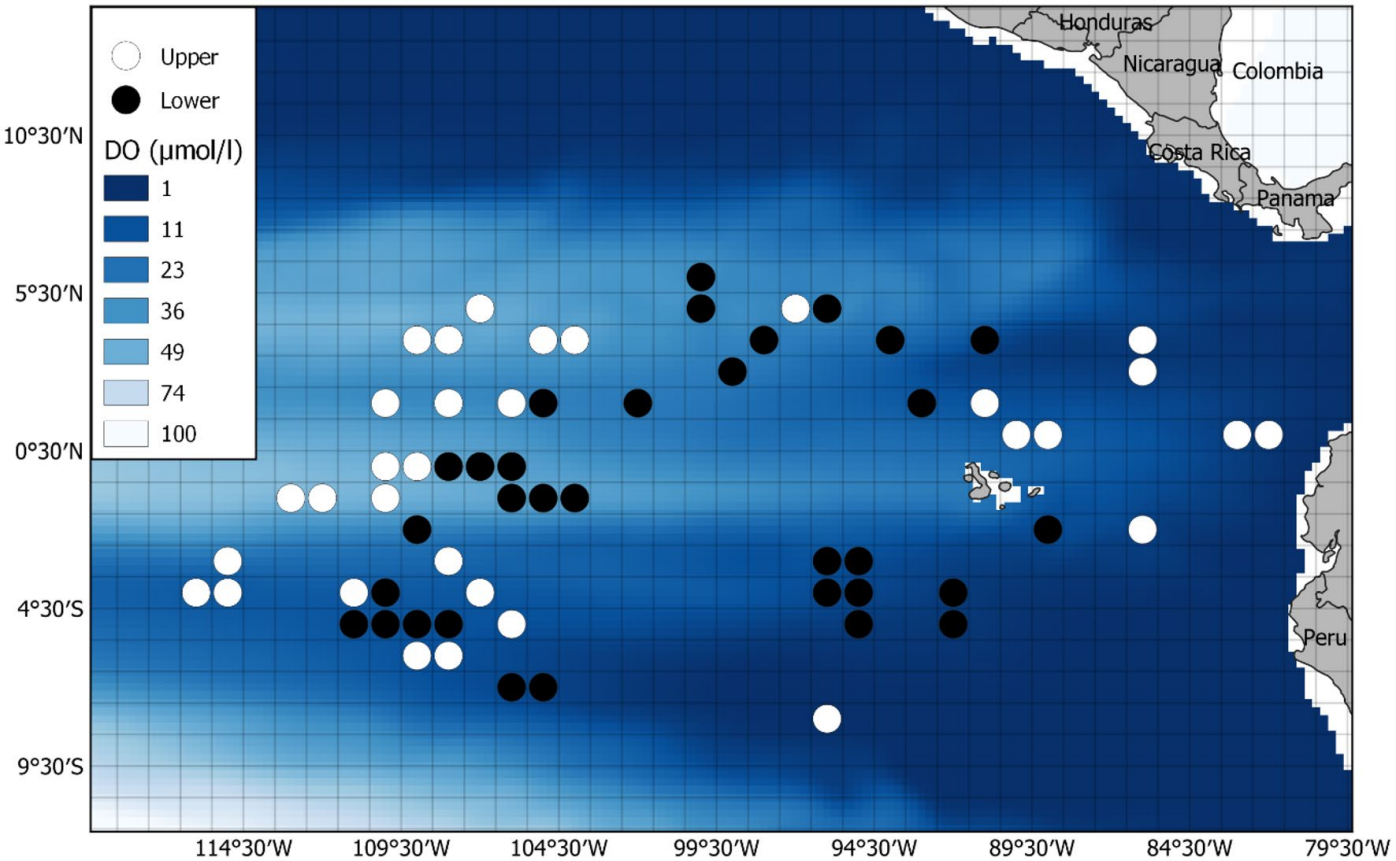
BET upper and lower 10th percentile grid cells. Grid cells where occupancy above 55 m is in the upper 10th percentile are shown in white; those in the lower 10th are shown in black. Background is mean DO from 2005 at 300 m. There is no clear pattern to the distribution of locations in relation to DO concentrations at 300 m

**Fig. 10:**
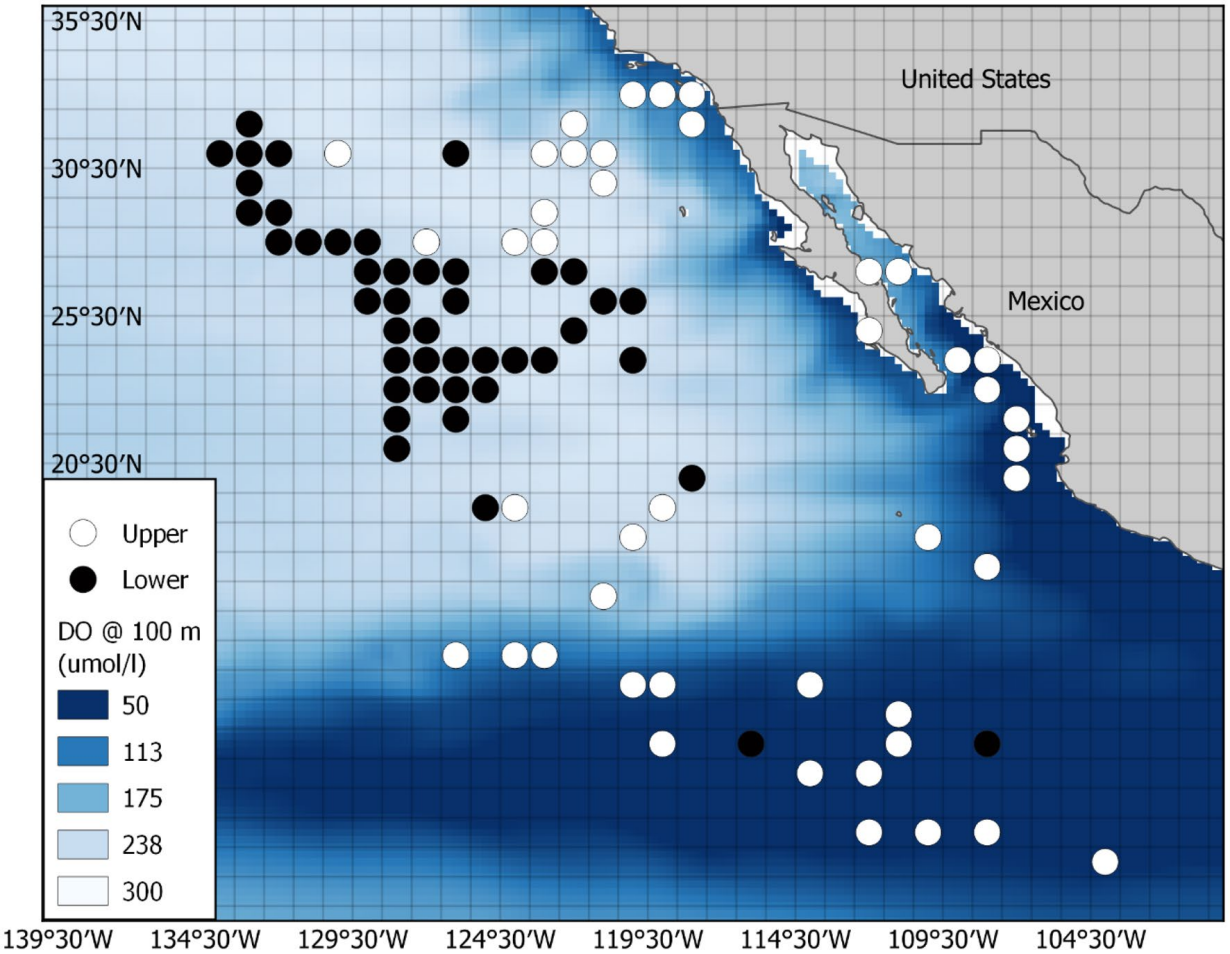
YFT upper and lower 10th percentile grid cells. Grid cells where occupancy above 43 m is in the upper 10th percentile are shown in white; those in the lower 10th are shown in black. Background is mean DO from 2005 at 100 m. Unlike with BET, there is a pattern to the distribution of locations in relation to DO concentrations at 100 m, with more cells in the lower 10th percentile in high DO areas and more of the upper 10th percentile cells in lower DO areas

Using these depth thresholds (55 and 43 m for BET and YFT, respectively), we then selected grid cells where occupancy of depths below the threshold was in the 10th and 90th percentiles (Figs. 9 and 10).

### Summary of DO and temperature levels encountered

**Fig. 11:**
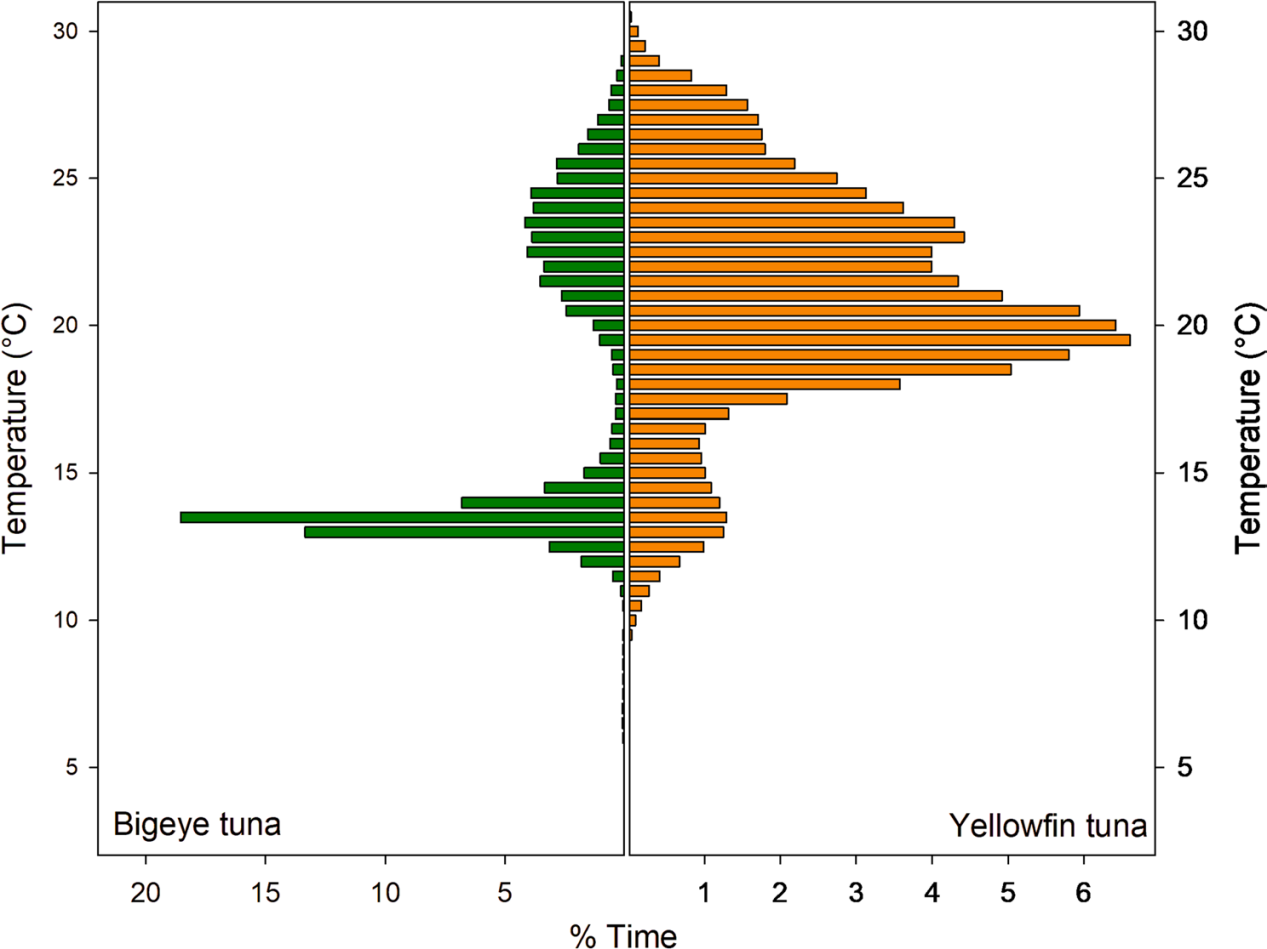
Daytime time-at-DO. BET (green) spent most of their active time in hypoxic water, while YFT (orange) spent almost all their time in DO concentrations > 200 µmol/l. BET also spend time at relatively high DO concentrations >  = 200 µmol/l. Solid red reference line is the 63 µmol/l hypoxic threshold. Data are from daytime locations, between the hours of 06:00 and 17:00 local time

**Fig. 12:**
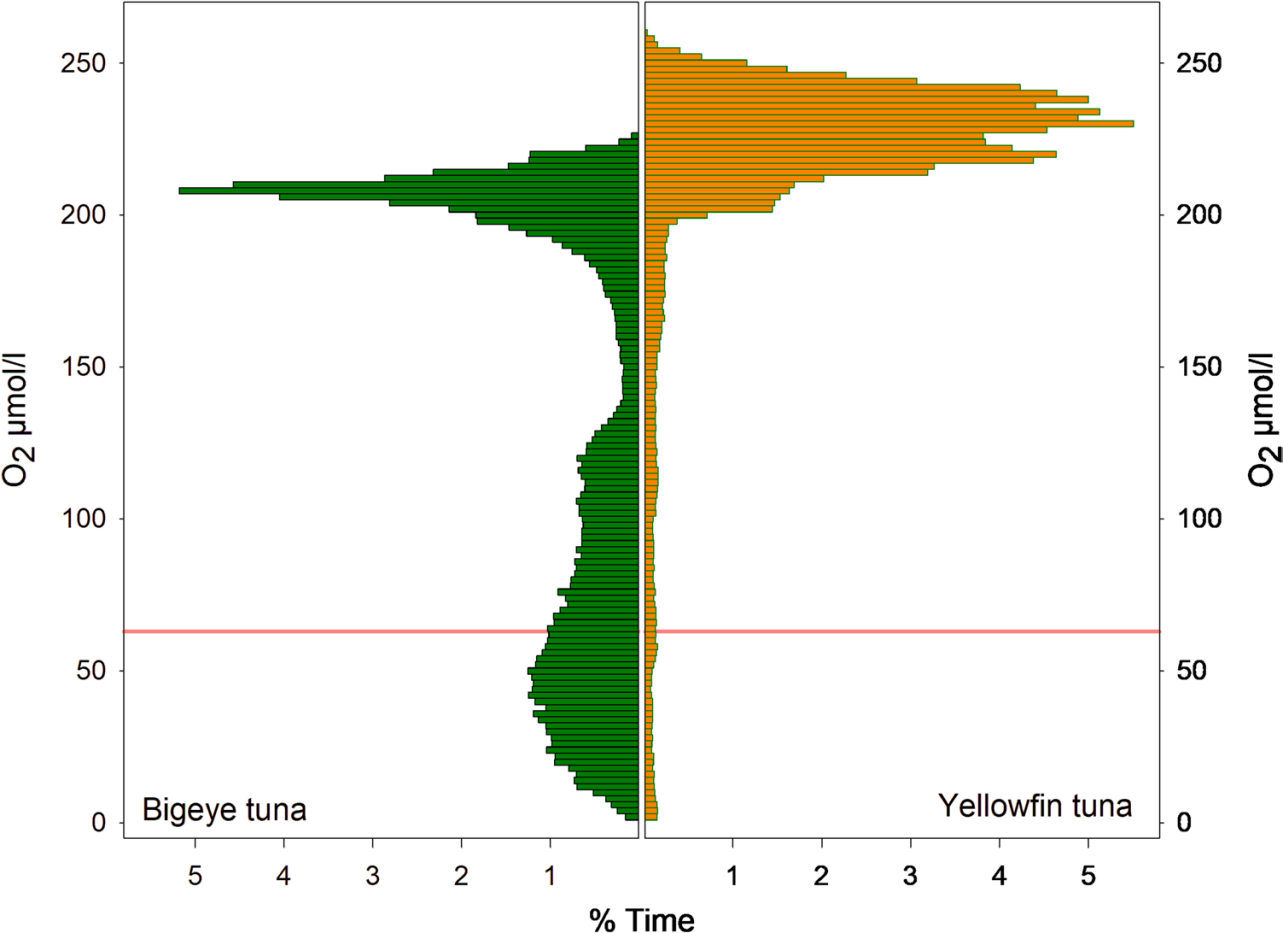
Daytime time-at-temperature. BET (green) spent more time at low temperatures than YFT (orange), reflecting time spent in cooler deeper water below the thermocline

To examine the extent to which the tunas were exposed to low DO, the DO values for all daytime (06:00 to 17:00 local time) locations were used to produce time-at-DO histograms with DO binned at 5 µmol/l (Fig. 11). BET spent 49.2% of their time in low DO waters below the hypoxic threshold of 63 µmol/l, while YFT spent much less time in low DO waters, remaining above 200 µmol/l for 85.5% of their time. However, it is also clear that YFT do visit hypoxic waters and are on occasion exposed to DO levels as low as BET, but for a much shorter time, spending only 3.5% of their time-at-DO below 63 µmol/l. The amount of time each species spent at different water temperatures was also determined (Fig. 12). YFT spent 59.8% of their time in waters warmer than 20 °C, while BET spent 53.6% of their time in cooler waters below 15 °C, with both temperatures reflecting the times spent at different depths by the two species. Therefore, YFT spent more time in higher DO and higher temperatures, but it is not clear whether it is temperature or DO that restricted their daytime depth distribution.

### Responses to DO and temperature

Here, we compare bathymetry, DO, and temperature at a range of depths between grid cells where the tunas were spending more or less time below the behavioural depth thresholds (55 m for BET, 43 m for YFT). For clarity, we refer to areas where more time is spent above the threshold as ‘shallow’ and where more time is spent below as ‘deep’. For BET we found that in the upper 10th percentile of grid cells (deep areas), 90% of their time was spent below 55 m, whereas in the upper 10th percentile of grid cells (shallow), 76% of time was spent above 55 m. The grid cells selected in these 10th percentiles therefore represent two extremes of occupancy of the water column, as confirmed by the median depths being 197 and 49 m in the deep and shallow areas, respectively.

**Fig. 13:**
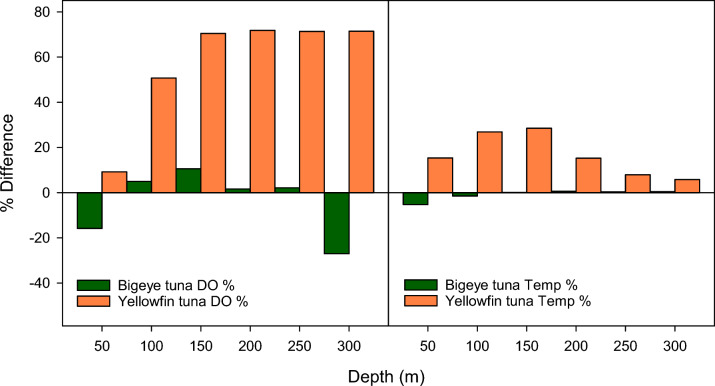
Differences in DO and temperature. Difference in DO concentration and temperature between the upper and lower 10% of 1-degree grid cells based on time spent below the depth thresholds of 55 m (BET) or 43 m (YFT). For YFT, there are significant differences with DO being consistently higher in deep regions. For BET, there is no clear pattern. For YFT, the differences in temperature are smaller than the differences in DO and show a reduction with increasing depth. For BET the differences in temperature are negligible

**Fig. 14:**
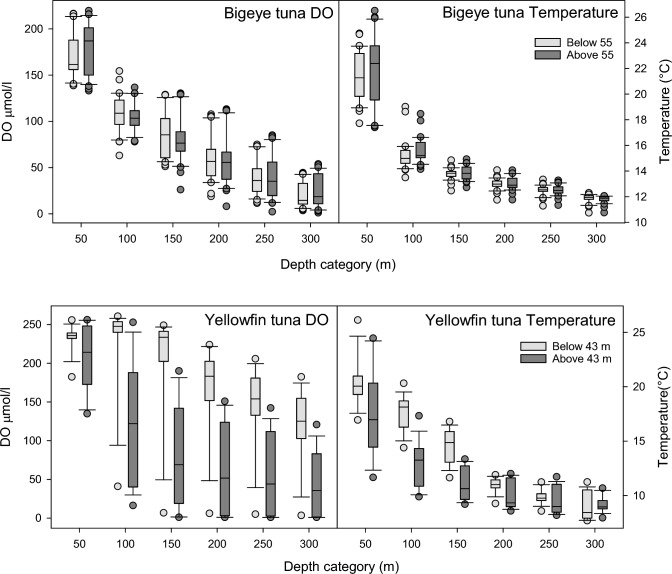
Box plots of DO and temperature above and below the behavioural depth thresholds. From these plots, it is clear that the differences in both DO and temperature are much greater for YFT. In particular, DO values are much greater at all depths below 50 m in deep areas. For BET, the reverse is true, with DO values being lower in deep areas. For YFT, in deep areas, temperatures are also higher, whereas for BET, the differences are very small

Overall, we found that for BET DO was lower at depths of 50 and 300 m; however, these differences were not significant and correlations between time spent below 55 m and DO were weak and not significant at any depth (Figs. 13, 14, Supplementary Table S3,). In contrast, for YFT at all depths, DO was significantly higher and was positively and significantly correlated with time spent below 43 m.

Differences and correlations for both species with temperature were lower (Figs. 13, 14, Supplementary Table S4), particularly for BET where the greatest difference was at 50 m (− 5.25%) and none of the differences were significant. Correlations were also weak and only significant at depths below 150 m. For YFT, while differences in temperature were smaller than found with DO, temperatures were all higher in deep areas. Correlations between time spent below 43 m and temperature were all positive and significant up to 150 m, which represents the limit of most of the vertical occupancy.

We found significant differences in median and maximum daily depths between the deep and shallow areas for BET (Supplementary Table S5), with maximum depth in deep areas being 806 m compared to 289 m in shallow areas. Median depth in deep areas was also greater at 174 m compared to 42 m and correlations between time below 55 and median and maximum depths were also positive and significant. We also computed correlations between the median depth in each location and DO at depths from 50 to 300 m (Supplementary Table S6). For BET, there was no significant correlation at any depth; however, for YFT, there were similar and significant positive correlations between median depth and DO at all depths analysed, suggesting that median depth increases with increasing DO at depth.

### BET vertical excursions

**Fig. 15:**
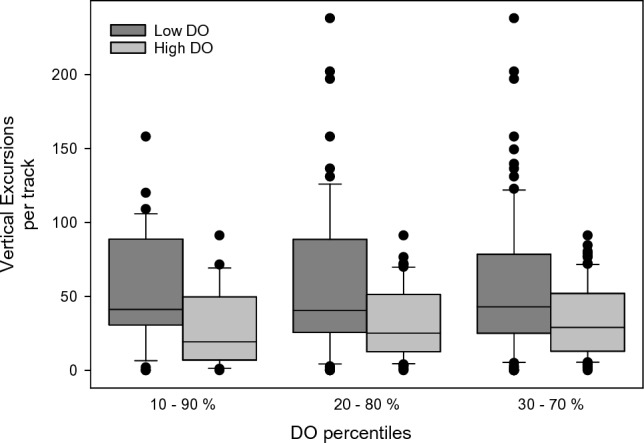
Number of vertical excursions per individual observed in low and high DO (10th–90th, 20th–80th, and 30th–70th percentiles, respectively) 1-degree grid cells

The analysis of time-at-depth in low DO grid cells showed that BET spent significantly more time above 55 m (20%, *p* < 0.001 Mann–Whitney Rank Sum Test). To test whether BET perform more upward vertical excursions and consequently spend more time in shallower, more oxygenated waters when DO at depth is low, we counted the number of vertical excursions in the upper and lower 10th percentile of grid cells for each individual. We found significantly more vertical excursions per individual in low DO areas (Table S7, Fig. 15; low DO median 41.25, high DO median 19.33, *p* = 0.005, Mann–Whitney Rank Sum test). There was no significant difference in temperature between high and low areas (low DO median temperature 19.61, high DO median temperature 20.91, *p* = 0.374). Extending the analysis, we also compared the number of vertical excursions between grid cells in the 80th v 20th, 70th v 30^th^, and 60th v 40th percentile pairs. Figure 15 (and Supplementary Table S7) shows that there were significantly more vertical excursions up to the 60–40 division of grid cells, but no significant differences in temperature, confirming that it was not only at the extremes of DO concentrations where low DO was associated with an increased number of vertical excursions.

To test the hypothesis that body size (fork length) is negatively correlated with the number of vertical excursions, a Pearson product moment correlation was performed which showed a negative correlation with *r*^2^ = − 0.503, slope = − 0.009. The associated scatter plot (Supplementary Figure S2), however, revealed two distinct clusters of points, where the BET tagged in 2000 appeared to be a different cohort to those tagged in years 2003–2005. A Mann–Whitney rank sum test comparing the length of the two groups confirmed this difference (Table S8, *p* < 0.001), and consequently, the two groups were analysed separately, revealing similar, positive correlation coefficients of *r*^2^ = 0.351, slope = 0.003 and *r*^2^ = 0.356, slope = 0.004 for BET 2003–2005 and BET 2000, respectively (Supplementary Figure S3, Table S8).

### Occurrence of exceptional deep dives

For BET, we found more and significantly deeper deep dives in the lower 10th percentile of DO grid cells (*p* < 0.001, Mann–Whitney Rank Sum Test, Supplementary Table S9). For YFT, however, while there were more deep dives in low DO grid cells, there were significantly deeper dives where DO was higher (*p* = 0.015, Mann–Whitney Rank Sum Test). However, for YFT, the sample size was considerably smaller than with BET (only 47 dives in total, compared to 710 for BET). To test whether, where DO at 300 m was low, DO at deeper depths might be higher, we plotted mean DO at depths up to 2000 m in each low DO and high DO grid cell. However, this showed no difference in DO concentrations below about 400 m (Supplementary Figure S6).

### Generalised additive modelling results

In all the detailed analysis models, where individual values were computed from time-series data for maximum daily depth, average daily depth, and, for BET, daily number of vertical excursions, the model that included the track ID as a random factor, to account for individual variation, proved to be the better model (Table 1). The implication is that in all these cases, individual variability has a greater effect on the response variable than either temperature or DO. These results are presented in full in the OSM.

**Table 1:** Summary of GAM analysis results

Detailed response variable	Best distribution	Best model	% deviance
BET daily vertical excursions	Gamma (link = identity)	s(Track, bs = re)	23.7
BET daily maximum depth	Gaussian	s(Track, bs = re)	8.6
BET daily average depth	Gaussian (link = log)	s(Track, bs = re)	21.5
YFT daily maximum depth	Gaussian (link = identity)	s(Track, bs = re)	16.7
YFT daily average depth	Gamma (link = inverse)	s(Track, bs = re)	25.6
Spatial response variable			
BET time below 55 m	Gaussian	s(SST) + s(DO100) + s(DO300)	18.9
BET maximum depth	Inverse Gaussian (link = 1/μ^2^)	s(DO150) + s(Temp200) + s(SST)	59.7
BET average depth	Gamma (link = identity)	s(DO300) + s(Temp150) + s(SST)	14.7
BET vertical excursions	Gamma (link = inverse)	s(DO150) + s(Temp50) + s(SST)	71.6
YFT time below 43 m	Gaussian (link = log)	s(Temp150) + s(SST)	50.2
YFT maximum depth	Inverse Gaussian (link = 1/μ^2^)	s(DO100) + s(SST)	28.4
YFT average depth	Gamma (link = inverse)	s(DO150) + s(Temp150) + s(SST)	44.7

Detailed response results are those derived from analysis of the depth time-series data. Spatial response results are those derived from the data selected from the 1-degree grid cells. Daily response variables were derived for each individual track using locations between the hours of 06:00 to 17:00 local time (Pacific Time, UTC-08:00 h or Mountain Standard Time, UTC-07:00 h depending upon tuna position; see Methods). Spatial response variables were computed from each occupied 1-degree grid cell, again between the hours of 06:00 and 17:00

GAM analysis of the spatially derived metrics (derived from grid cell locations) resulted in much higher percentages of deviance explained, and consequently, these models were explored in more detail (Table 1).

### BET model outputs

For time spent below 55 m, DO at 100 m explained the most deviance at 5.9%. DO at 300 m was the second highest scoring DO variable with 5.67% deviance explained. As these two values were close and much higher than the next best (DO150 at 1.72%), both DO100 and DO300 were used together with SST and temperature at 300 m in the multi-variate models. The model selected by AIC, included DO100, DO300, and SST, and explained 18.9% deviance. The model combining DO100, DO300, SST, and Temp300 explained slightly more deviance (19.7%), but the additional parameter resulted in a lower wAIC value (Supplementary Tables S28, S29 and S30). However, the plot of the GAM model (Supplementary Figure S18) shows a complex relationship with no clear trend.

For BET maximum depth, DO at 150 m and temperature at 200 m were selected as the most important depths for the analysis, explaining 19.6 and 26.5% of deviance, respectively. However, DO at 100 m explained 16.6%, and temperature at all other depths explained between 17.4 and 25.4%. Therefore, although the model selected by AIC was DO150 + Temp200 + SST, explaining 59.7%, it is clear that temperature at all depths is probably important for maximum depth. The GAM plots (Supplementary Figure S20) do not show clear trends, however, rather indicate complex relationships. Maximum depth appears to peak at DO concentrations of around 135 µmol/l, while increasing temperature appears to reduce the maximum depth (Supplementary Tables S31, S32 and S33).

For BET mean depth, the most important depths for DO and temperature were DO300 and Temp150, respectively. In both cases, the deviance explained by these factors was considerably more than factors at other depths. The model selected by AIC, includes DO300, Temp150, and SST, explaining 14.7%. The effect of all variables was complex, however. Over the range of most observations, increasing DO reduces average depth, as does SST. With Temp150, average depth is at a maximum at around 13.75 °C, although the difference between minimum and maximum depths is minimal (~ 80 to ~ 100 m). Overall, average depth is not greatly affected by temperature or DO (Supplementary Tables S34, S35 and S36).

DO at 150 m and temperature at 50 m were the most import factors for the number of BET vertical excursions observed in each grid cell, accounting for 38.1 and 41.3% of the deviance, respectively (Supplementary Tables S37 and S38). However, other depths were also important for both DO and temperature. For DO, the depths of 100 and 200 m accounted for 23.2 and 20.7% of deviance and for temperature all depths of 150 m and below accounted for at least 32% of the deviance. SST was also found to account for 45.3% of the deviance and the model selected by AIC included DO, Temperature, and SST, accounting for 71.6% of the deviance (Supplementary Table S39). The plots (Supplementary Figure S24) suggest that the number of vertical excursions exhibits a strong peak at lower temperatures (~ 23.5 °C), which then tails off. The effect of DO shown in the plot exhibits a strong peak at about 110 µmol/l; however, below ~ 90 µmol/l, there is a fairly constant lower rate, rather than an increase, which might be expected if low DO is driving vertical excursions. Despite the relatively high value of 71% of deviance explained, there is no clear relationship evident in any of the plots of the variables involved. DO at all depths contributes at least 9% of the deviance explained; however, again, there is no clear relationship evident in any of the plots shown in supplementary Figure S25.

### YFT model outputs

For time spent below 43 m, DO and temperature at 150 m explained 34.6 and 39.9% of deviance, respectively, making them the most significant variables. DO at all depths below 150 m accounted for ~ 30% deviance and temperature at 100 m accounted for 34.5% deviance, suggesting that DO and temperature throughout the water column are important, rather than at specific depths. SST alone accounted for 19.3% and the model selected by AIC included temperature at 150 m and SST, accounting for 50.2% deviance. There was a clear increase in time below 43 m with increasing temperatures at 150 m (Supplementary Figure S27). The relationship with SST is more complex, with an apparent peak at just over 20 °C. There is a clear relationship between time below 43 m and DO concentrations, with time below increasing sharply once DO exceeds 150 μmol/l.

For maximum depth, DO and temperature at 100 m are the most important factors, accounting for 24.5 and 4.09% of deviance, respectively. DO at 150 m might also be important, accounting for 17.1%. SST accounts for slightly more deviance than temperature at 100 m (5.1%) and the model selected by AIC includes DO and SST, accounting together for 28.4% deviance. The plots (Supplementary Figure S29) indicate peaks in max depth at DO concentrations around 200 µmol/l and temperatures around 23 °C.

For mean depth, DO and temperature at 150 m were the most important factors at 27.3 and 42.7% deviance explained, respectively. However, for DO, all depths below 150 m explained at least 20% deviance and temperature at 100 m explained 38%. Generally, % deviance explained by all depths was higher than with other metrics. SST explained 21.4% and the selected model included temperature and SST, explaining 44.1% of deviance. The plots (Supplementary Figure S31) show that above DO concentrations of 150 µmol/l, average depth increases markedly. Above 10 °C average depth also increases steadily. The SST plot is more complex, with a peak in average depth at just over 20 °C.

## Discussion

Here, we investigated the extent to which DO concentrations at depth act as a limiting factor in BET and YFT daytime depth distributions by analysing observed vertical distributions (time-at-depth) with DO and temperature at a range of depths. Determining the role of low DO in shifting fish distributions is important as, in many geographical regions, the distribution of both BET and YFT overlaps known oxygen minimum zones (OMZs) (Schaefer et al. 2007; Schaefer and Fuller 2009), where biological and hydrographic processes combine to produce hypoxic regions at depth (Stramma et al. 2008a).

Previous studies (e.g., Schaefer et al. 2009) have shown that when in the equatorial eastern Pacific, BET are able to stay for prolonged periods at depth, tracking the deep scattering layer (DSL) only performing brief vertical excursions to shallower, warmer and better oxygenated waters. YFT were found to be searching at similar depths close to the DSL for similar prey items, but spend little time-at-depth, instead tending to perform repetitive bounce dives between deep cold, low DO waters and shallower, warmer and better oxygenated waters (Schaefer et al. 2009). This difference in behaviour is likely because YFT lack some the physiological adaptations of BET, such as transactional heat transfer, that allows BET to retain heat in swimming muscles (Holland et al. 1992; Boye et al. 2009).

The analysis of time-at-DO performed here showed that there was a clear difference between BET and YFT, with BET spending 28% of their time-at-DO concentrations below the proposed 63 µmol/l hypoxic threshold (Breitburg et al. 2018) and 68% of their time below 200 µmol/l, while YFT spend 85% of their time above 200 µmol/l. This difference in time-at-DO could simply result from differing patterns of vertical space use, driven by different foraging strategies and target prey species, rather than by limitations imposed by low DO. For example, BET characteristically dive to depths of around 250 to 300 m to forage on vertically migrating prey close to the deep scattering layer, such as myctophids, with fish comprising a greater proportion of the diet than in YFT (Menard et al. 2006). YFT, however, although increasing their average depth during daytime hours, restrict most of their activity to above 200 m, (Schaefer et al. 2009; Schaefer and Fuller 2010; Matsumoto et al. 2013; Fuller et al. 2015) where crustaceans such as pelagic red crab (*Grimothea planipes*) are more important prey items than with BET (Menard et al. 2006). As a result of these foraging behaviours and prey preferences, the time-at-DO and time-at-temperature profiles differ considerably between the two species.

By analysing the time-at-depth profiles from areas (sets of 1-degree grid cells) at the two extremes of DO concentration at depths relevant to the probable foraging depths of the two species, we were able to identify clear threshold depths at which behaviour changed between high and low DO areas. Interestingly, the threshold depths we identified differed from the depths at which we used the DO concentration to separate the 1-degree areas. For BET, we used DO at 300 m and found behavioural thresholds at 55 and 185 m, while for YFT, we used DO at 100 m and found a threshold at 43 m. These differences between the depth used to define low and high DO areas, and the depths at which changes of behaviour were observed suggest that behavioural responses to DO in the water column are complex and that the fish are not simply limited by a particular oxycline. It is also possible that the actual DO levels experienced by the fish might be more heterogeneous than represented in the modelled DO used in the study, a possibility that future work using a tag that measures DO in situ on tagged fish, would help to explore.

By then categorising grid cells by the time spent above and below these behavioural thresholds, we were able to analyse the response to DO in relation to environmental variables at multiple depths, without specifying DO levels or hypoxic thresholds directly. This is important, as comparatively little is known about physiological responses to hypoxia in free-living fish and setting thresholds and expectations a priori could bias the results. Our results confirmed that there was a much stronger correlation between time-at-depth and DO concentrations for YFT than for BET. At all depths, DO was significantly higher in areas where YFT spent more time below the observed 43 m threshold and conversely, in regions where DO at depth was low, YFT spent significantly more time above 43 m, which is as expected if YFT are avoiding areas and depths where DO concentrations were low. We also showed that, for YFT, the median depth was positively correlated with DO concentrations at depth, further supporting either the avoidance of low DO water, or the preference for high DO water, in this species. The results from the GAM analysis support these findings, with DO at 150 m accounting for 35% of deviance in time spent below 43 m and DO at 100 m being the most important factor in determining maximum depths reached (24.5% of deviance). For average depth as well, DO at 150 m explained 27% of the deviance and there was a clear increase in average depth at DO concentrations above 150 µmol/l. We can conclude that DO likely represents a limiting factor for YFT vertical distributions throughout their vertical range, and that, as a result, expanding OMZs are likely to cause shifts in YFT horizontal and vertical distributions in those geographic regions where they overlap.

BET, by contrast, show no clear shift in vertical distribution in relation to DO below depths of around 55 m; instead, there was a trend of decreasing DO at depth in the areas where BET spent more time below 55 m. This is the opposite of what would be expected if BET were avoiding low DO, suggesting that the DO concentration was less important in driving vertical habitat use. BET continued to forage at depth, even at hypoxic DO concentrations, as evidenced by the time-at-DO analysis performed here. Similarly, in the analysis of time spent above and below the second threshold identified of 185 m, we again found no significant differences in DO or temperature at any depth. In the GAM analysis, DO at 100 m explained 5.9% of deviance for time below 55 m, and was shown to be as important as temperature at 300 m or SST, which explained 5.13 and 5.92%, respectively. In combination, DO at 100, temperature at 300 and SST explain 18.9% of the deviance, which suggests that, together, these factors are likely important drivers of the vertical distributions observed. However, the relationship between DO and time below 55 m in the GAM plots was complex and no clear trend emerged. For maximum and average depths as well, the GAM analysis found DO to be the most important factor, but again, the plots did not show a clear trend, as seen with YFT, instead showing a peak at low DO, which could simply be the result of deeper diving encountering lower DO concentrations.

For both species, the GAM analysis provided no unequivocal relationships between any measured behaviour (e.g., maximum depth) and any environmental factor investigated, despite the level of deviance explained suggesting otherwise in some cases (e.g., BET vertical excursions at 71%). To identify changes in behaviour associated with differing DO at depth, it was necessary to compare the extremes of DO (10th and 90th percentiles), which suggests that while differences were identified between species, the relationship between these changes in behaviour and environmental factors are complex and are confounded with many other factors, of which individual variation plays a significant role. In the analysis of individual time-series data, individual variation, possibly influenced to some extent by the differing sizes (fork lengths) of the fish, obscured any other relationships. Intra-specific variation of this nature can often confound the determination of drivers of behaviour and, consequently, many studies (including this one) resort to larger scale approximations from averages or aggregations of population level data (Lubitz et al. 2022).

For a high oxygen demand predator like BET to forage at such low DO concentrations, some adaptations to low DO are expected. Some of these are known to be physiological, such as BET haemoglobin having higher oxygen affinity than either yellowfin or skipjack tunas (Lowe et al. 2000; Mislan et al. 2017). Further, their specific blood chemistry has been shown to release more O_2_ when the blood is warmed when re-entering muscle tissue than either yellowfin or skipjack tunas, releasing more O_2_ where it is most in demand (Lowe et al. 2000). Nonetheless, when ambient DO is at hypoxic concentrations, well below 63 µmol/l, O_2_ stored in blood haemoglobin or muscle myoglobin, will eventually be depleted and oxygen debt will be incurred. Consequently, we hypothesised that the characteristic upward vertical excursions performed by BET when foraging would provide an opportunity to replenish blood oxygen, as well as rewarming the body. If this were the case, then we would expect there to be an increase in the number of upward vertical excursions in areas where DO is lower and, indeed, our results supported this hypothesis. BET performed four times as many upward vertical excursions in the lowest DO areas compared to the highest DO areas; however, we found no concomitant significant difference in temperature. By extending the analysis to compare areas where the difference in DO was less (e.g., to 60–40th percentiles), we found that the number of vertical excursions was still significantly different; however, we found no significant difference in temperature between these areas. These results suggest that in areas where DO is below the 40th percentile, DO likely becomes a more important driver of upward vertical excursions, with a principal function being to replenish blood oxygen. Interestingly, the GAM analysis found temperature in shallow (50 m) water to be more important than temperature at depth, explaining 41% of deviance, and with more vertical excursions being performed when surface waters were cooler. As the extent of rewarming that will occur in cooler shallow waters will be less than when the shallow water is warmer, but with cooler water typically having higher DO which will replenish blood O_2_, these results conform to our hypothesis. DO at depth (150 m) was also found to be important, explaining 38% deviance, but here the modelling suggested that more vertical excursions were performed when DO was higher. Again, however, the GAM does not necessarily identify causal relationships and it is possible that more activity took place when DO at depth was higher, possibly because prey was more abundant. More activity would likely result in an increase in the number of upward vertical excursions to repay an oxygen debt, as increased activity will consume more oxygen; however, at the same time, the increased activity would contribute to maintaining body temperature, through the warming of muscle tissue. Consequently, we propose, based on these results, that activity at depth was limited more by DO than by temperature, at least for the individuals in the habitats analysed.

It was noted by Brill (2007) that the prolonging of the repayment of oxygen debt by low ambient DO, following exercise, might limit habitat suitability, and therefore, levels of DO at depths shallower than the activity depth may also be important to both species. The results presented here appear to support this idea. DO was found to be lower at all depths in locations where the tunas shifted to shallower depths and, for both species, the depth at which a change in behaviour was noted was shallower than the depth at which DO concentrations were used to identify high and low DO areas. The effect of body size (fork length) on the number of vertical excursions performed did not, in this case, support the hypothesis that the increase in thermal inertia resulting from an increase in body size would reduce the number of vertical excursions performed. While the initial investigation seemed to confirm this, it was evident that pooling the data obscured differences between tunas tagged in 2000 compared to later years. Analysing each cohort of fish separately showed that both groups shared a similar response, with increasing body length resulting in a small increase in the number of vertical excursions (3 to 4 more per day per metre of body length). It is possible that this small increase results from an increase in size-specific post-prandial O_2_ consumption (specific dynamic action, SDA) related to consumption and processing of a meal (Fitzgibbon et al. 2007; Fitzgibbon and Seymour 2009). For example, southern bluefin tuna (*Thunnus maccoyii*) were found to increase swimming speed depending on the meal size consumed, leading to an increase in ventilation across the gills, presumably to counter the increase in O_2_ consumption resulting from higher SDA (Fitzgibbon et al. 2007). However, this would only be the case if the meal consumed was proportionally larger in comparison to body size. Another factor that could lead to larger fish performing more vertical excursions in low DO waters is that, although increased body size acts to retain body heat, the same is not true for oxygen, with larger bodies requiring more oxygen; thus, while larger fish will tend to stay warmer, this would only increase their oxygen requirements. It is most likely that both temperature and oxygen act in concert to determine the specific threshold at which an individual is forced to abandon foraging and ascend to shallower, warmer, oxygen enriched waters to recover, before diving again.

It was interesting and somewhat contrary to expectations that the number and depth of exceptional deep dives for both species was found to be greater in regions where DO at 100 m (for YFT) or 300 m (for BET) was lower. DO concentrations at the depth of the deep dives were found to be slightly but significantly lower for deep dives performed in low DO areas, which given the significantly deeper dives is expected (Table S10). However, this does suggest that the tunas are not diving to below the OMZ. This analysis has unfortunately not added to our understanding of why species such as tuna perform these exceptionally deep dives, and this remains an interesting question for further research.

A limitation of our study was that we could not determine from these observations whether the tunas were shifting to shallower, DO enriched waters because of their own physiological limitations or preferences, or whether they were following the shifting distributions of prey species. Work in the tropical and equatorial Atlantic suggests that the typical mesopelagic prey species of BET, such as myctophids, phosichthyids, and gonostomatids (Bertrand et al. 2002a; Schaefer and Fuller 2009; Karuppasamy et al. 2011) may be tolerant of low DO and able to migrate through OMZ regions along with other prey taxa and to remain at depth (Olivar et al. 2017). Therefore, the behaviour of BET might represent physiological preferences and limitations. For YFT it might be that the preferred prey species of YFT (juvenile fish and crustaceans) do have a lower tolerance of low DO, and that the vertical distribution of YFT more closely reflects that of its prey (Bertrand et al. 2002b). A further limitation pertains to the scale and nature of the modelled DO used here, and in the positional accuracy of the estimated tuna locations. The modelled DO at a scale of 0.25-degree (~ 25 km) represents a large-scale homogenous gradient of DO which very likely differs from the nature of actual DO experienced by the tunas, where micro-scale eddies and currents will contribute to increased heterogeneity. This problem of scale is exacerbated by the errors in location accuracy resulting from the light level geolocation, which can be as much as 1 degree of longitude and 2 degrees of latitude (Lam et al. 2010). Consequently, it is not possible at present to study in detail the responses of individual fish, and therefore, a larger scale spatial analysis, despite its limitations, was more appropriate.

Nonetheless, the results here suggest that if OMZs increase in volume or shoal to shallower depths, as expected due to further climate-driven deoxygenation, then the habitat occupied by tuna, especially YFT, will also be shifted and compressed, potentially altering their susceptibility to capture, regardless of whether it is the tunas’ DO intolerances or that of their prey that drives the shifts. While tunas represent the higher end of metabolic oxygen demand in water-breathing marine predators, it is likely that many other important marine apex predators, such as marlin, sailfish, and lamniform sharks having high metabolic rates, will be similarly affected by low DO as has been shown recently for ectothermic blue sharks (Vedor et al. 2021b).

## Conclusions

Both species respond to low DO in different ways. BET do not significantly adjust their depths in response to lower DO; however, they do increase the number of upward vertical excursions they perform, which reduces the time available for foraging. YFT, on the other hand, forage in shallower depths when DO is lower; however, whether this is because of YFT’s physiological intolerance of lower DO or a response to hypoxia-induced shifts in prey distributions remains to be determined. With climate-driven decreases in DO at depth, YFT are likely to shift their depth and possibly horizontal distribution as a result. There is also the further possibility that if YFT shift their vertical distributions to shallower depths, this could make them increasingly vulnerable to capture by commercial fishing vessels, particularly purse-seines.

For BET, while activity depth is less likely to be affected, the increased number of upward vertical excursions will reduce time spent at depth and increase time spent in shallower water. As with YFT, if BET spend more time at shallower depths, then there could be an increased susceptibility to capture by longlines or purse-seines. BET might also be affected as a result of prey species’ responses to lower DO; if prey species are forced into shallower, more oxygen-rich water then this habitat compression could benefit BET and reduce the impact due to increased vertical excursions. Again though, this would place BET in shallower water where susceptibility to capture might be increased. There could therefore be multiple detrimental effects on survival and reproductive capacity for both species, exacerbating the existing impacts from industrialised fishing. The increased occupancy of shallower waters predicted here should be accounted for in stock assessments as well as in mitigating their increased vulnerability to fishing. Future research would benefit considerably from tagging studies using a tag that can measure DO in situ, so that the actual DO concentration encountered by the fish could be determined. Not only would the fish be acting as oceanographers, providing accurate information on the heterogeneity of DO at depth, but the data would allow actual DO tolerances and preferences to be determined, thus making a significant contribution to our understanding of the impact of expanding OMZs on marine ecosystems.

### Supplementary Information

Below is the link to the electronic supplementary material.

Supplementary file1 (PDF 2997 KB)

## Data Availability

The datasets generated during and/or analysed during the current study are not publicly available as they are owned and archived by the Inter-American Tropical Tuna Commission, but are available from the corresponding author on reasonable request.
