# Supplementary material for: Highly active fish in low oxygen environments: vertical movements and behavioural responses of bigeye and yellowfin tunas to oxygen minimum zones in the eastern Pacific Ocean
Source: Mar Biol. 2024 Jan 13;171(2):55. doi: 10.1007/s00227-023-04366-2 (PMC10787700; doi:10.1007/s00227-023-04366-2)
Supplement: Supplementary file 1 — Supplementary file1 (PDF 2997 KB) [file 227_2023_4366_MOESM1_ESM.pdf]

Supplementary information

Supplementary tables

Table S1: Summary of BET location and dive time series data

Tag Number	Length	Horizontal Locations	Vertical Locations	Track Start Date	Track End Date	Track Elapsed Time (H)	Release Latitude	Release Longitude	Re-Capture Latitude	Re-Capture Longitude
0390041 2003	860	131	282924	2003-03-27	2003-10-09	4715	-1.980	-95.180	-2.000	-95.300
0390041 2004	950	204	523138	2004-05-11	2005-05-09	8719	-2.200	-94.700	0.000	-98.010
0390045 2004	950	148	268573	2004-05-11	2004-11-13	8419	-2.200	-94.700	-2.700	-99.420
0390045 2005	630	63	115988	2005-04-15	2005-07-04	1933	-1.980	-95.180	0.480	-99.120
0390052 2003	810	85	156163	2003-05-07	2003-08-23	2603	2.000	-95.330	0.000	-101.290
0390055 2004	850	20	57009	2004-05-11	2004-06-19	950	-2.200	-94.700	-2.570	-96.170
0390056 2003	880	63	136265	2003-05-07	2003-08-09	2271	2.000	-95.330	0.800	-98.620
0390057 2003	800	236	478797	2003-03-27	2004-02-22	7980	-1.980	-95.180	0.130	-95.080
0390057 2004	970	87	285885	2004-05-09	2004-11-23	7243	-1.980	-95.170	0.000	-110.020
0390060 2003	1210	236	261761	2003-04-05	2003-10-04	4363	-1.980	-95.180	0.130	-95.080
0390061 2003	830	159	313387	2003-04-05	2003-11-08	5223	-1.980	-95.180	3.050	-108.380
0390063 2004	870	141	241308	2004-05-11	2004-10-25	4022	-2.200	-94.700	-2.850	-98.780
0390065 2003	770	193	375548	2003-03-27	2003-12-12	6259	-1.980	-95.180	-1.050	-93.480
0390069 2003	820	107	194046	2003-05-07	2003-09-18	3234	2.000	-95.330	-2.270	-109.280
0390070 2004	950	117	234060	2004-05-11	2004-10-20	3901	-2.200	-94.700	-2.280	-95.980
0390070 2005	640	160	289160	2005-04-15	2005-11-01	9134	-1.940	-95.180	6.250	-106.880
0390072 2003	900	98	158240	2003-05-07	2003-08-25	2637	2.000	-95.330	-1.730	-107.580
0390075 2004	870	142	304533	2004-05-11	2004-12-08	5076	-2.200	-94.700	-0.530	-94.480
0390075 2005	780	311	614197	2005-04-15	2006-06-15	10237	-1.940	-95.180	-1.980	-95.180
0390080 2003	760	88	140343	2003-03-27	2003-07-02	2339	-1.980	-95.180	-2.820	-97.980
0390081 2004	950	392	828763	2004-05-11	2005-12-07	13813	-2.200	-94.700	-1.580	-98.170
0390095 2003	770	99	168246	2003-04-05	2003-07-30	2804	-1.980	-95.180	1.680	-99.480
0390100 2004	850	140	333418	2004-05-11	2004-12-28	5557	-2.200	-94.700	-0.070	-95.670
0490915 2005	790	90	261545	2005-04-16	2005-10-14	4359	-1.980	-95.180	-0.320	-95.500
0490916 2005	760	60	97354	2005-04-16	2005-06-22	1623	-1.980	-95.180	0.870	-104.120
0590053 2005	770	205	324980	2005-04-16	2005-11-27	5416	-1.940	-95.180	-1.000	-95.080
0590054 2005	770	78	135092	2005-04-16	2005-07-18	2253	-1.940	-95.180	0.850	-103.880
1082 2004	880	137	403869	2004-05-08	2005-02-12	6731	-1.980	-95.170	1.200	-99.480

Tag Number	Length	Horizontal Locations	Vertical Locations	Track Start Date	Track End Date	Track Elapsed Time (H)	Release Latitude	Release Longitude	Re-Capture Latitude	Re-Capture Longitude
1082 2005	600	75	116524	2005-04-15	2005-07-05	1942	-1.940	-95.180	-3.230	-105.750
1102 2003	570	357	345505	2003-04-14	2003-12-10	5758	-1.980	-95.480	3.820	-124.720
1103 2004	1010	79	239733	2004-05-09	2004-10-22	3996	-1.980	-95.170	-2.470	-96.020
1103 2005	870	38	92042	2005-04-15	2005-06-18	1534	-1.940	-95.180	0.050	-102.700
1105 2003	1090	357	110311	2003-05-12	2003-07-27	1839	-1.980	-95.480	3.820	-124.720
1107 2003	1190	165	320421	2003-04-07	2003-11-15	5340	-1.980	-95.180	-1.520	-98.620
1107 2004	890	50	65333	2004-05-08	2004-06-22	1089	-2.000	-95.200	2.430	-98.430
1110 2003	1000	43	95797	2003-04-13	2003-06-18	1597	-1.980	-95.180	0.230	-100.340
1111 2004	880	130	284453	2004-05-07	2004-11-20	4741	-1.980	-95.170	-2.870	-101.840
1113 2003	1150	177	306097	2003-04-10	2003-11-08	5102	-1.980	-95.180	-1.500	-104.120
1116 2003	690	319	147558	2003-04-05	2003-07-16	2459	-1.980	-95.180	1.420	-98.740
1117 2004	870	102	234008	2004-05-09	2004-10-18	3900	-1.980	-95.170	-2.470	-97.640
2308 2004	880	43	61350	2004-05-08	2004-06-19	1022	-1.980	-95.170	-2.570	-96.180
2312 2005	730	29	96124	2005-04-15	2005-06-20	1602	1.940	-95.180	0.850	-105.370
2313 2004	830	145	335133	2004-05-08	2004-12-26	5586	-1.980	-95.170	-0.520	-94.780
2318 2004	540	43	62831	2004-05-07	2004-06-19	1047	-1.980	-95.170	-2.570	-96.170
2325 2004	840	111	206912	2004-05-09	2004-09-29	3449	-1.980	-95.170	-0.930	-102.670
2325 2005	730	122	283219	2005-04-15	2005-10-28	4720	-1.940	-95.180	1.180	-103.710
2326 2004	870	116	238462	2004-05-08	2004-10-20	3974	-1.980	-95.170	-1.250	-95.930
2330 2005	760	80	131778	2005-04-15	2005-07-15	2196	-1.940	-95.180	0.250	-104.030
2332 2004	870	116	285961	2004-05-09	2004-11-23	4766	-1.980	-95.170	-0.950	-102.220
2540 2004	1230	214	221114	2004-05-08	2004-10-08	3685	-1.980	-95.170	-1.170	-102.220
2541 2004	880	166	290615	2004-05-08	2004-11-25	4844	-1.980	-95.170	-2.000	-100.410
2541 2005	770	54	102061	2005-04-15	2005-06-25	1701	-1.940	-95.180	0.000	-103.000
2633 2005	990	53	78471	2005-04-15	2005-06-08	1308	-1.940	-95.180	3.750	-107.520
4009 2005	740	89	145110	2005-04-16	2005-07-25	2418	-1.940	-95.180	-1.780	-94.200
4010 2005	740	54	80785	2005-04-15	2005-06-10	1346	-1.940	-95.180	0.000	-95.200
4016 2005	630	204	318546	2005-04-16	2005-11-23	5309	-1.940	-95.180	0.550	-96.270
4019 2005	650	68	109028	2005-04-15	2005-06-29	1817	-1.940	-95.180	0.000	-96.100
4023 2005	780	179	314560	2005-04-16	2005-11-20	5243	-1.940	-95.180	-1.370	-99.950
4026 2005	760	60	110513	2005-04-16	2005-07-01	1842	-1.940	-95.180	0.000	-91.700
4030 2005	780	30	73030	2005-04-15	2005-06-09	1333	-1.940	-95.180	0.020	-99.000
4035 2005	780	55	153204	2005-04-16	2005-07-31	2553	-1.940	-95.180	0.000	-93.360
4036 2005	770	35	59545	2005-04-16	2005-05-27	994	-1.940	-95.180	-3.070	-98.500
4070 2005	800	181	318989	2005-04-15	2005-11-22	5316	-1.940	-95.180	-0.780	-93.950

Tag Number	Length	Horizontal Locations	Vertical Locations	Track Start Date	Track End Date	Track Elapsed Time (H)	Release Latitude	Release Longitude	Re-Capture Latitude	Re-Capture Longitude
4092 2005	690	96	167922	2005-04-15	2005-08-09	5093	-1.940	-95.180	3.720	-98.730
4100 2005	670	53	78708	2005-04-15	2005-06-08	1312	-1.940	-95.180	3.750	-107.520
99-787 2000	1150	116	46489	2000-04-15	2000-08-22	3099	0.870	-97.100	1.720	-83.080
99-792 2000	1260	156	65990	2000-04-15	2000-10-15	4399	0.870	-97.100	1.350	-95.820
99-793 2000	1140	168	114274	2000-04-15	2001-02-26	7618	0.870	-97.100	0.000	-95.170
99-798 2000	1060	256	98606	2000-04-17	2001-01-16	6574	1.470	-96.970	0.000	-99.660
99-801 2000	1120	71	28844	2000-04-17	2000-07-06	1923	1.470	-96.970	1.470	-90.710
99-803 2000	1130	76	34050	2000-04-23	2000-07-26	3663	2.020	-95.400	2.200	-102.720
99-804 2000	1040	164	63096	2000-04-17	2000-10-09	4211	1.470	-96.970	0.000	-103.870
99-810 2000	1200	45	20301	2000-04-23	2000-06-18	1353	2.020	-95.400	-1.650	-93.170
99-812 2000	1160	136	57401	2000-04-23	2000-09-29	3827	2.020	-95.400	0.550	-109.000
99-814 2000	1200	164	119996	2000-04-23	2001-04-18	8792	2.200	-95.400	0.000	-93.090
99-816 2000	1220	37	13490	2000-04-23	2000-05-30	899	2.200	-95.400	1.780	-97.600
99-817 2000	1060	54	22360	2000-04-17	2000-06-18	1491	1.470	-96.970	-1.120	-95.870
99-821 2000	1100	273	129290	2000-04-15	2001-04-10	8630	0.380	-97.250	0.000	-96.390
99-826 2000	1180	224	97993	2000-04-17	2001-01-14	6533	1.470	-96.970	0.000	-99.370
99-835 2000	1090	53	20360	2000-04-23	2000-06-18	1357	2.020	-95.400	1.200	-99.680
99-839 2000	1090	54	20230	2000-04-17	2000-06-12	1349	1.470	-96.970	-2.330	-95.750
99-847 2000	1020	43	15147	2000-04-17	2000-05-29	1010	1.470	-96.970	1.770	-97.230
99-860 2000	1040	55	19478	2000-04-17	2000-06-10	1298	1.470	-96.970	-2.180	-97.870
99-862 2000	930	38	23067	2000-04-18	2000-06-21	1538	1.720	-96.800	-1.770	-94.800
99-865 2000	1010	50	21172	2000-04-17	2000-06-15	1416	1.470	-96.970	-2.670	-99.050
99-869 2000	1130	107	40608	2000-04-16	2000-08-07	2707	1.080	-97.080	4.850	-103.180
99-876 2000	990	252	91614	2000-04-15	2000-12-26	6108	0.870	-97.100	0.000	-94.520
99-883 2000	1160	51	21730	2000-04-19	2000-06-18	1449	1.930	-96.550	-1.600	-95.550
99-884 2000	1020	159	115361	2000-04-15	2001-03-01	7691	0.870	-97.100	0.000	-94.140
99-886 2000	960	121	128317	2000-04-17	2001-04-08	9316	-1.370	-95.850	-1.170	-95.700
99-887 2000	1200	128	92481	2000-04-23	2001-01-12	6346	2.020	-95.400	0.000	-100.100
99-889 2000	1120	66	27671	2000-04-16	2000-07-02	3628	1.080	-97.080	0.370	-98.580

Table S2: Summary of YFT location and dive time series data

Tag Number	Length	Horizontal Locations	Vertical Locations	Track Start Date	Track End Date	Track Elapsed Time (H)	Release Latitude	Release Longitude	Re-Capture Latitude	Re-Capture Longitude
0390081	1324	394	1133354	2007-02-23	2008-03-24	9468	18.32	-114.72	14.53	-112.05
0490916	1406	188	542510	2007-02-23	2007-09-19	5000	18.32	-114.72	18.5	-114.08
0990499	1412	290	835200	2010-04-24	2011-02-08	6983	18.32	-114.75	18.63	-114.92
1090003	1104	204	587520	2010-02-15	2010-09-13	5063	18.33	-114.75	17.91	-112.7
1090012	1104	161	460959	2010-02-15	2010-07-27	3865	18.33	-114.75	18.55	-114.48
1090013	1095	351	1010880	2010-02-15	2011-01-31	8423	18.33	-114.75	18.37	-114.72
1090024	1223	151	434910	2010-02-15	2010-07-26	3864	18.33	-114.75	18.55	-114.48
1090025	764	564	1624598	2010-02-15	2011-09-15	13850	18.33	-114.75	18.47	-114.5
1090062	921	593	1707621	2010-04-22	2012-02-06	15718	18.32	-114.75	18.35	-114.78
1090064	1134	94	270774	2010-04-22	2010-07-26	2280	18.32	-114.75	18.55	-114.48
1090072	929	481	1385280	2010-04-21	2011-10-26	13295	18.97	-112.05	9.47	-110.33
1090391	1125	245	707623	2011-02-19	2011-10-26	5992	18.33	-114.74	18.08	-114.87
1090397	657	153	442996	2011-02-16	2011-07-20	3715	19.02	-112.04	23.8	-112.22
1090400	1213	122	353602	2011-02-19	2011-06-23	2994	18.33	-114.74	19.73	-114.27
1090441	1080	306	881886	2011-04-18	2012-03-17	8021	18.78	-110.9	19.5	-110.64
1090467	1056	145	418320	2011-04-22	2011-09-15	3509	18.367	-114.65	18.47	-114.5
1190040	930	302	869760	2011-05-10	2012-04-22	8375	18.77	-110.9	16.42	-109.45
1190051	666	106	305867	2011-05-10	2011-08-28	2644	18.983	-112.067	23	-111.25
1190063	1213	337	970916	2011-05-08	2012-04-23	8426	18.7	-110.917	16.13	-110.12
A0478	910	558	802862	2002-10-13	2004-04-23	13381	25.73	-113.13	8.27	-119.83
A0509	930	298	428237	2002-10-12	2003-08-06	7137	25.73	-113.13	22.07	-109.33
A0525	900	1160	1398240	2002-10-13	2005-12-16	27840	25.73	-113.13	23.58	-111.7
A0549	850	200	286561	2003-10-09	2004-04-25	4776	29.07	-118.23	22.4	-111.08
A0644	980	298	429120	2002-10-13	2003-08-06	7151	25.73	-113.13	20.3	-109.18
A0806	730	281	403201	2002-10-12	2003-07-19	6720	25.73	-113.13	20.72	-111.47
A0826	940	288	413281	2002-10-13	2003-07-27	6888	25.73	-113.13	23.38	-111.17
A0827	760	155	221761	2003-10-15	2004-03-17	3696	25.25	-112.8	23.55	-108.85
A1425	920	292	419041	2003-10-09	2004-07-26	6984	29.07	-118.23	29.08	-118.24
A1446	670	354	508321	2004-11-08	2005-10-27	8472	25.23	-112.82	23.44	-110.6
A1448	870	199	285121	2003-10-09	2004-04-24	4752	29.07	-118.23	22.83	-110.53
A1455	850	200	286561	2003-10-09	2004-04-25	4776	29.07	-118.23	22.4	-111.08
A1461	670	194	277921	2003-10-16	2004-04-26	4632	25.72	-113.12	22.75	-111.25
A1504	660	92	131041	2004-11-08	2005-02-07	2184	25.23	-112.82	23.52	-111.12
A1506	750	253	362881	2003-10-15	2004-06-23	6048	25.1	-112.75	24.08	-112.57
A1514	830	473	417966	2003-10-09	2004-07-26	6966	29.07	-118.23	23.4	-111.17

Tag Number	Length	Horizontal Locations	Vertical Locations	Track Start Date	Track End Date	Track Elapsed Time (H)	Release Latitude	Release Longitude	Re-Capture Latitude	Re-Capture Longitude
A1526	780	186	266401	2003-10-15	2004-04-17	4440	25.25	-112.8	22.55	-110.97
A1547	770	238	341281	2003-10-15	2004-06-08	5688	25.25	-112.8	23.67	-112.08
A1550	860	192	275041	2003-10-09	2004-04-17	4584	29.07	-118.23	22.68	-110.88
A1559	750	194	277921	2003-10-15	2004-04-25	4632	25.25	-112.8	22.33	-110.98
A1565	840	155	223200	2005-10-15	2006-03-18	3719	24.93	-115.75	28.91	-118.24
A1569	860	250	358561	2003-10-11	2004-06-16	5976	24.97	-115.76	20.47	-114.15
A1895	1300	177	253441	2003-10-12	2004-04-05	4224	24.97	-115.76	24.98	-115.77
B2677	670	92	245701	2004-11-08	2005-08-17	6768	25.23	-112.82	24.03	-112.37
B2682	620	134	359101	2004-11-06	2005-03-19	3192	24.97	-115.78	22.57	-110.68
B2683	680	142	383400	2004-11-05	2005-03-26	3407	24.97	-115.78	23.58	-111.7
B2687	690	508	1368901	2004-11-05	2006-03-27	12168	24.97	-115.78	22.17	-111.08
B2707	680	134	359101	2004-11-06	2005-03-19	3192	24.97	-115.78	22.57	-110.68
B2711	610	273	734401	2004-11-05	2005-08-04	6528	24.97	-115.78	31.18	-118.28
B2712	620	321	1386719	2004-08-15	2005-07-01	7703	30.95	-116.86	23.6	-111.65
B2720	660	514	1314899	2004-11-08	2006-04-08	12384	25.23	-112.82	23.68	-111.77
B2730	750	206	553501	2004-11-08	2005-06-01	4920	25.23	-112.82	21.93	-112.03
B2734	640	316	850501	2004-11-05	2005-09-16	7560	24.97	-115.78	26.35	-113.63
B2743	740	348	936901	2004-08-16	2005-07-29	8328	31.34	-117.3	30.57	-118
B2767	570	355	955801	2004-08-15	2005-08-04	8496	30.96	-116.85	30.25	-116.65
C0003	720	373	2775598	2004-11-05	2005-11-13	8975	24.97	-115.78	21.18	-107.08
C0005	660	122	1306801	2004-11-08	2005-03-09	2904	25.23	-112.82	22.35	-110.88
C0007	730	203	2181601	2004-11-08	2005-05-29	4848	25.23	-112.82	23.37	-109.25
C0009	750	244	2624401	2004-11-05	2005-07-06	5832	24.97	-115.78	24.96	-115.77
C0017	680	388	2800797	2004-11-08	2006-02-17	11207	25.23	-112.82	22.17	-111.08
C0022	980	198	855360	2004-11-09	2005-05-25	4751	23.31	-110.65	23.44	-110.6
C0024	960	250	1800000	2004-11-07	2005-07-14	5999	24.97	-115.78	24.95	-115.77
C0026	690	185	799200	2004-11-06	2005-05-09	4439	24.97	-115.78	24.23	-113.07
C0036	810	106	756001	2004-11-09	2005-02-22	2520	23.31	-110.65	23.47	-112.1
C0041	680	213	1526401	2004-11-05	2005-07-25	6288	24.97	-115.78	21.82	-111.57
C0043	600	260	2789481	2004-08-20	2005-05-07	6217	31.53	-117.51	25.75	-113.53
C0044	820	120	514081	2004-11-06	2005-03-05	2856	24.97	-115.78	23	-110.85
C0046	690	118	505441	2004-11-08	2005-03-05	2808	24.97	-115.78	23.05	-111.18
C0053	650	159	682561	2004-11-09	2005-04-16	3792	23.31	-110.65	24.68	-108.68
C0054	680	106	453601	2004-11-08	2005-02-21	2520	25.23	-112.82	23.85	-111.28
C0055	600	238	1706401	2004-08-20	2005-04-14	5688	31.46	-117.53	30.43	-116.52

Tag Number	Length	Horizontal Locations	Vertical Locations	Track Start Date	Track End Date	Track Elapsed Time (H)	Release Latitude	Release Longitude	Re-Capture Latitude	Re-Capture Longitude
C0066	610	394	2829601	2004-08-20	2005-09-17	9432	31.46	-117.53	32.27	-119.05
C0121	830	249	1071361	2005-08-04	2006-04-09	5952	31.27	-118.33	23.5	-111.8
C0140	850	249	1071361	2005-08-04	2006-04-09	5952	31.52	-118.33	23.57	-111.78
C0174	860	250	1075681	2005-08-04	2006-04-10	5976	31.5	-118.31	23.57	-111.65
C0179	840	248	1067041	2005-08-04	2006-04-08	5928	31.52	-118.33	22.48	-111.63
C0194	800	243	1045441	2005-08-04	2006-04-03	5808	31.55	-118.3	22.53	-111.42
C0202	870	331	1425601	2005-08-04	2006-06-30	7920	31.63	-118.27	23.82	-109.4
C0203	780	202	868321	2005-08-04	2006-02-21	4824	31.73	-118.2	23.57	-111.53
C0204	830	248	1067041	2005-08-04	2006-04-08	5928	31.55	-118.3	23.57	-111.93
C0908	820	221	950401	2005-08-04	2006-03-12	5280	31.63	-118.27	25.57	-126.28
C0908B	1471	269	726994	2007-02-22	2007-11-18	6462	18.32	-114.72	18.43	-114.43
C0911	810	249	1071361	2005-08-04	2006-04-09	5952	31.55	-118.3	23.57	-111.78
C0953	850	242	2602801	2005-08-10	2006-04-08	5784	31.57	-118.95	23.57	-111.93
C0980	770	163	699841	2005-10-16	2006-03-27	3888	25.25	-112.8	22.52	-110.6
C0991	800	175	751681	2005-10-16	2006-04-08	4176	25.25	-112.8	23.57	-111.93
C0996	830	177	760321	2005-10-16	2006-04-10	4224	25.25	-112.8	22.53	-110.8
C1029	780	129	552961	2005-10-15	2006-02-20	3072	24.93	-115.75	22.8	-111.62
C1033	840	134	574561	2005-10-16	2006-02-26	3192	25.25	-112.8	22.52	-112.33
C1063	830	129	2764801	2005-08-11	2005-12-17	3072	31.57	-119.02	22.3	-111.52
C1072	810	128	548641	2005-10-15	2006-02-19	3048	24.93	-115.75	23.52	-111.7
C1079	780	173	747360	2005-10-15	2006-04-05	4151	24.93	-115.75	28.62	-116.28
C1086	950	368	1585441	2005-10-15	2006-10-17	8808	24.93	-115.75	24.95	-115.79
C1215	960	262	1127521	2005-10-15	2006-07-03	6264	24.93	-115.75	24.95	-115.76
C1226	910	64	1360801	2005-10-15	2005-12-17	1512	31.57	-118.95	23.63	-111.82
C1236	830	113	483841	2005-10-16	2006-02-05	2688	25.25	-112.8	23.02	-110.7
D0029	830	130	557281	2005-10-15	2006-02-21	3096	24.93	-115.75	23.57	-111.61
D0038	720	127	544321	2005-10-16	2006-02-19	3024	25.25	-112.8	23.52	-111.7
D0042	610	273	1175041	2005-10-14	2006-07-13	6528	24.93	-115.75	31.1	-117.62
D0043	820	129	552961	2005-10-16	2006-02-21	3072	25.25	-112.8	23.57	-111.61
D0045	800	134	574561	2005-10-15	2006-02-25	3192	24.93	-115.75	23.17	-111.37
D0064	980	235	1010881	2005-10-15	2006-06-06	5616	24.93	-115.75	24.98	-115.76
D0098	1163	472	678957	2006-02-20	2007-06-07	11315	18.34	-114.69	16.98	-114.3
D0135	850	242	1045440	2005-08-10	2006-04-08	5807	31.53	-118.9	16.92	-112.63
D0604	1017	456	655966	2006-02-19	2007-05-21	10932	18.32	-114.75	18.58	-114.7
D0606	1396	635	914649	2006-02-20	2007-11-18	15268	18.34	-114.69	18.43	-114.43

Tag Number	Length	Horizontal Locations	Vertical Locations	Track Start Date	Track End Date	Track Elapsed Time (H)	Release Latitude	Release Longitude	Re-Capture Latitude	Re-Capture Longitude
D0609	810	201	288001	2006-11-05	2007-05-24	4800	23.63	-112.28	19.65	-108.88
D0622	1125	632	909208	2006-02-20	2007-11-14	15153	18.34	-114.69	18.4	-114.67
D0632	921	385	554090	2006-02-21	2007-03-14	9258	18.33	-114.7	18.62	-114.88
D0741	1017	386	555276	2006-02-20	2007-03-14	9278	18.34	-114.69	18.62	-114.88
D0742	1095	154	222250	2006-02-20	2006-07-24	3704	18.34	-114.69	20.77	-114.83
D0744	1387	144	208562	2006-02-21	2006-07-16	3500	18.34	-114.69	16.85	-117.5
D0755	1114	469	674536	2006-02-20	2007-06-04	11242	18.34	-114.69	18.33	-114.83
D0796	770	197	282241	2007-11-28	2008-06-11	4704	23.67	-111.92	22.98	-111.5
D0846	860	129	184321	2006-11-05	2007-03-13	3072	23.63	-112.28	19.53	-109.13
D0847	710	201	288001	2006-11-03	2007-05-22	4800	24.9	-115.75	21.95	-110.82
D1104	990	217	311041	2007-11-29	2008-07-02	5184	23.5	-111.92	22.75	-112.77
D1109	630	271	388801	2006-11-03	2007-07-31	6480	24.9	-115.75	32.08	-117.42
D1110	710	361	519840	2006-11-06	2007-11-01	8663	23.63	-112.28	6.9	-102.97
D1111	600	271	388801	2006-11-03	2007-07-31	6480	24.9	-115.75	31.75	-117.1
D1144	710	271	388801	2006-11-03	2007-07-31	6480	24.9	-115.75	24.9	-115.75
D1146	710	259	371521	2006-11-07	2007-07-23	6192	25.22	-112.8	24.17	-112.53
D1153	690	140	200161	2006-11-03	2007-03-22	3336	24.9	-115.75	23.15	-110.87
D1158	710	128	2743201	2006-08-05	2006-12-10	3048	29.672	-116.734	28.45	-116.42
D1164	720	127	2743200	2006-08-05	2006-12-09	3047	29.461	-116.557	24.45	-112.77
D1165	680	264	378721	2006-11-03	2007-07-24	6312	24.9	-115.75	25.42	-113.25
D1178	760	351	1512001	2006-08-05	2007-07-21	8400	29.461	-116.557	24.38	-113.37
D1181	770	134	191521	2007-11-29	2008-04-10	3192	23.5	-111.92	24.32	-109.07
D1184	830	215	308161	2006-11-05	2007-06-07	5136	23.63	-112.28	17.82	-114.52
D1191	740	128	2743201	2006-08-05	2006-12-10	3048	29.461	-116.557	31.75	-117.3
D1198	740	311	1339201	2006-08-05	2007-06-11	7440	29.461	-116.557	28.45	-116.42
D1199	740	260	372961	2006-11-07	2007-07-24	6216	25.22	-112.8	24.1	-112.62
D1209	1100	120	171361	2007-11-27	2008-06-09	4680	23.67	-111.92	22.92	-111.5
D1214	610	263	377281	2006-11-03	2007-07-23	6288	24.9	-115.75	24.43	-113
D1215	630	128	2743201	2006-08-05	2006-12-10	3048	29.621	-116.696	26.93	-115.78
D1217	730	128	2743201	2006-08-05	2006-12-10	3048	29.461	-116.557	27.17	-115.32
D1226	900	169	241921	2007-11-29	2008-05-15	4032	23.5	-111.92	23	-109.52
D1245	740	130	2786401	2006-08-05	2006-12-12	3096	29.461	-116.557	31.85	-117.5
D1257	690	130	2786401	2006-08-05	2006-12-12	3096	29.461	-116.557	24.18	-113.18
D1260	650	120	2584922	2006-08-05	2006-12-02	2872	29.461	-116.557	32.28	-117.87
D1262	720	130	2786401	2006-08-05	2006-12-12	3096	29.672	-116.734	25.72	-113.13

Tag Number	Length	Horizontal Locations	Vertical Locations	Track Start Date	Track End Date	Track Elapsed Time (H)	Release Latitude	Release Longitude	Re-Capture Latitude	Re-Capture Longitude
D1281	760	263	2829601	2006-08-05	2007-04-24	6288	29.461	-116.557	32.18	-117.5
D1302	740	123	329401	2008-12-13	2009-04-14	2928	24.2	-111.5	23.17	-112.63
D1469	1090	217	312480	2007-11-29	2008-07-02	5207	23.5	-111.92	23.58	-111.8
D1474	1168	93	134366	2008-02-20	2008-05-23	2239	18.32	-114.62	18.38	-114.65
D1477	794	95	137160	2008-02-19	2008-05-24	2285	18.32	-114.67	18.5	-114.65
D1497	744	153	219470	2008-02-16	2008-07-18	3657	19	-112.07	19	-112.07
D1567	754	152	219254	2008-02-16	2008-07-17	3654	19	-112.07	18.97	-112.07
D1580	1036	206	296906	2007-02-16	2007-09-12	4996	18.77	-110.9	11.18	-109.71
D1589	1449	269	387517	2007-02-16	2007-11-12	6458	18.77	-110.9	10.62	-124.55
D1593	1396	397	564105	2007-02-16	2008-03-24	9647	18.77	-110.9	14.52	-112.27
D1602	1220	780	1121909	2007-11-27	2010-01-15	18698	23.67	-111.96	23.77	-112.07
D1611	740	191	273601	2007-11-29	2008-06-06	4560	23.5	-111.92	22.2	-112.23
D1612	670	348	499681	2007-11-29	2008-11-10	8328	23.5	-111.92	24.3	-108.37
D1627	1008	543	781920	2007-02-17	2008-08-12	13031	18.71	-110.9	18.37	-114.53
D1631	1263	93	132917	2007-02-16	2007-05-20	2215	18.77	-110.9	19	-112.33
D1641	970	98	139681	2007-11-29	2008-03-05	2328	23.5	-111.92	22.4	-110.5
D1642	630	116	165601	2007-11-29	2008-03-23	2760	23.5	-111.92	23.4	-107.55
D2030	1417	389	559963	2007-02-17	2008-03-12	9332	18.71	-110.9	6.27	-101.55
D2036	1438	401	577321	2007-02-17	2008-03-27	9694	18.71	-110.9	16.07	-112.72
D2050	1026	424	610498	2007-02-16	2008-04-16	10198	18.77	-110.9	17.68	-109.3
D3063	1193	505	727827	2008-02-18	2009-07-07	12130	18.32	-114.62	11	-98.05
D3099	1213	96	138701	2008-02-18	2008-05-24	2311	18.32	-114.62	18.5	-114.65
D3185	710	207	556201	2008-12-13	2009-07-07	4944	24.2	-111.5	23.5	-111.88
D3195	740	243	653401	2008-12-13	2009-08-12	5808	24.2	-111.5	24.22	-112.47
D3212	730	205	550801	2008-12-13	2009-07-05	4896	24.2	-111.5	23.02	-111.5
D3213	650	211	567001	2008-12-12	2009-07-10	5040	25.717	-113.333	23.55	-111.97
D3217	710	216	580501	2008-12-12	2009-07-15	5160	25.717	-113.333	24.02	-112.4
D3230	780	133	356401	2008-12-12	2009-04-23	3168	25.717	-113.333	23.83	-112.4
D3420	1350	300	433129	2008-04-23	2009-02-17	7218	18.35	-114.68	18.24	-114.72
D3436	978	152	219089	2008-04-24	2008-09-23	3651	18.72	-111	18.98	-113.65
D3438	881	438	630896	2008-04-24	2009-07-06	10514	18.72	-111	13.9	-109.48
D4396	1134	398	1077270	2009-02-16	2010-03-21	9575	19.333	-110.783	10.33	-109.17
D5090	1085	542	1463400	2009-04-21	2010-10-14	13007	18.317	-114.73	18.53	-114.82
D5146	1085	387	1044900	2009-04-17	2010-05-09	9311	18.7	-110.9	16.52	-109.27
D5158	1173	201	541164	2009-04-17	2009-11-04	4810	18.7	-110.9	13.7	-117.12

Supplementary analysis results

Validation of the 4-minute interpolation

To ensure that results would not be altered by the standardisation of the time-series data to 4-minute intervals (from 1-minute in the BET2003-2005 and YFT data) the longest BET track was further interpolated to 8, 12, 16, 20, 24 and 30 minutes. For each interpolation a Time at depth analysis was performed and the results verified using a scatter-plot and a Repeated Measures ANOVA. While there were differences in the TAD plots, these do not detract from the overall pattern and are not statistically significant (p = 1.0).

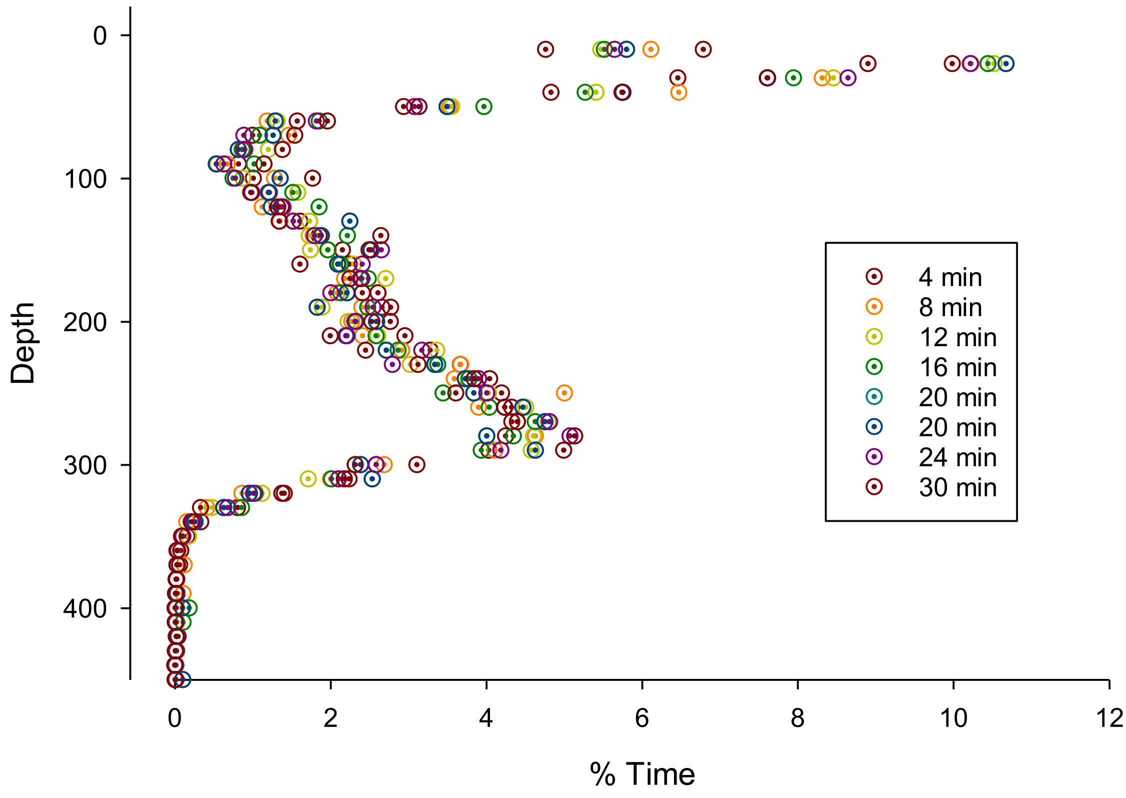

Figure S1: Verification of the interpolation to 4-minutes

One Way Repeated Measures Analysis of Variance

**Treatment Name**	**N**	**Missing**	**Mean**	**Std Dev**	**SEM**
4 min	133	0	0.752	1.589	0.138
8 min	133	0	0.752	1.704	0.148
12 min	131	0	0.763	1.701	0.149
16 min	133	0	0.752	1.648	0.143
20 min	133	0	0.752	1.675	0.145
24 min	131	0	0.763	1.703	0.149
30 min	122	0	0.820	1.691	0.153

Source of Variation	DF	SS	MS	F	P
Between Subjects	132	2519.724	19.089		
Between Treatments	6	0.000000638	0.000000106	0.00000338	1.000
Residual	777	24.437	0.0314		
Total	915	2544.617	2.781		

The differences in the mean values among the treatment groups are not great enough to exclude the possibility that the difference is due to random sampling variability; there is not a statistically significant difference (P = 1.000).

Results of checking the effect of removing FAD associated locations

To remove suspected FAD associated behaviour, along with post-tagging effects, the first 14 days were removed from all tracks prior to analysis. Analysis of the vertical behaviour throughout the BET tracks has identified numerous days where surface-oriented behaviour might be the result of FAD association. It was thought that the removal of putative FAD associations based on surface-oriented behaviour, rather than known associations, might unnecessarily bias the results through the removal of days where the tuna spent more time in surface waters when the actual cause of the surface orientation was not proven to be through FAD association. However, to check that this decision did not unduly affect the results, some of the preliminary analyses were repeated with the putative FAD associated days removed. To do so, a spreadsheet of BET location interpretations was used to set all locations that matched a tag number and date to 'FAD Associated' where the behaviour type column stated 'FAD'. This resulted in 268,302 of locations being set to FAD, of which, 106,446 were daytime locations which left 2,010,057 daytime non-FAD locations, so 5% of records are lost by filtering the putative FAD data. The results of the reanalysis are shown below.

Firstly, we computed daytime TAD plots with the FAD days removed and for just the FAD days. As expected, the FAD associated locations show a considerable surface bias, with about 75% of time spent above 50 m. The plot with the FAD days removed is indistinguishable from the original plot.

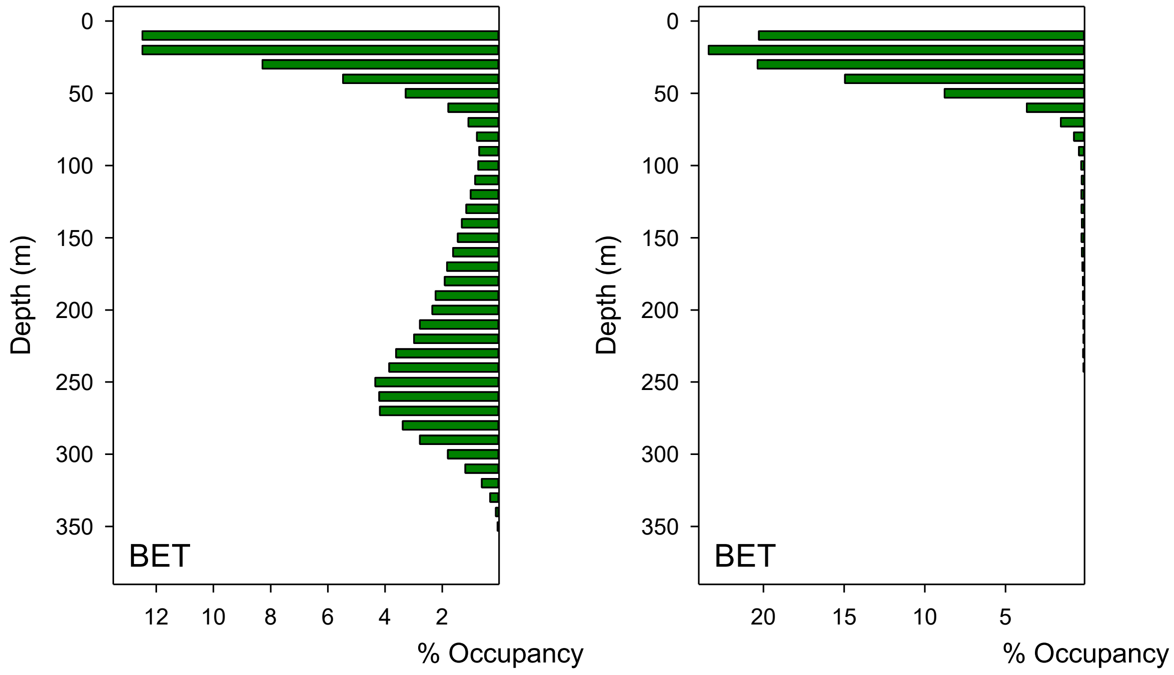

Figure S2: BET Time at depth plots from the FAD reanalysis

The plot on the left shows the TAD analysis with the FAD associated days removed. The plot on the left shows the TAD analysis of just the FAD days.

We then repeated the analysis of locations in grid cells where DO is in the upper or lower 10th percentile of DO at 100 m. This was important as this analysis determined the 55 m threshold for BET at which a shift in TAD was observed. Importantly, this threshold is conserved, as is the less important 185 m threshold.

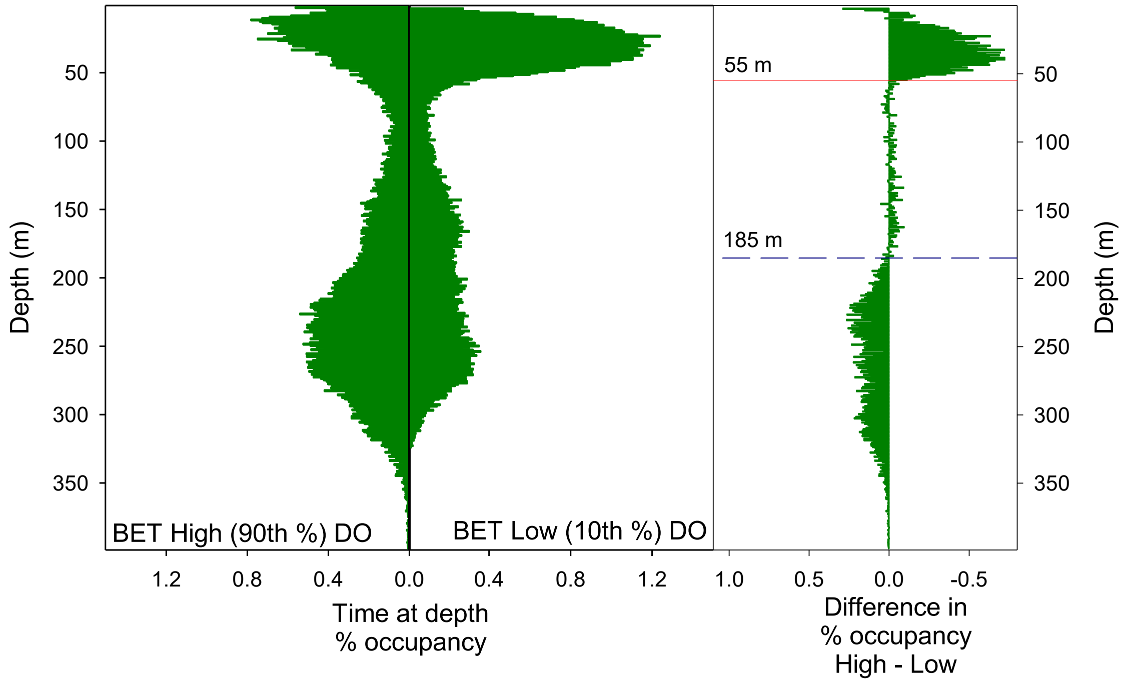

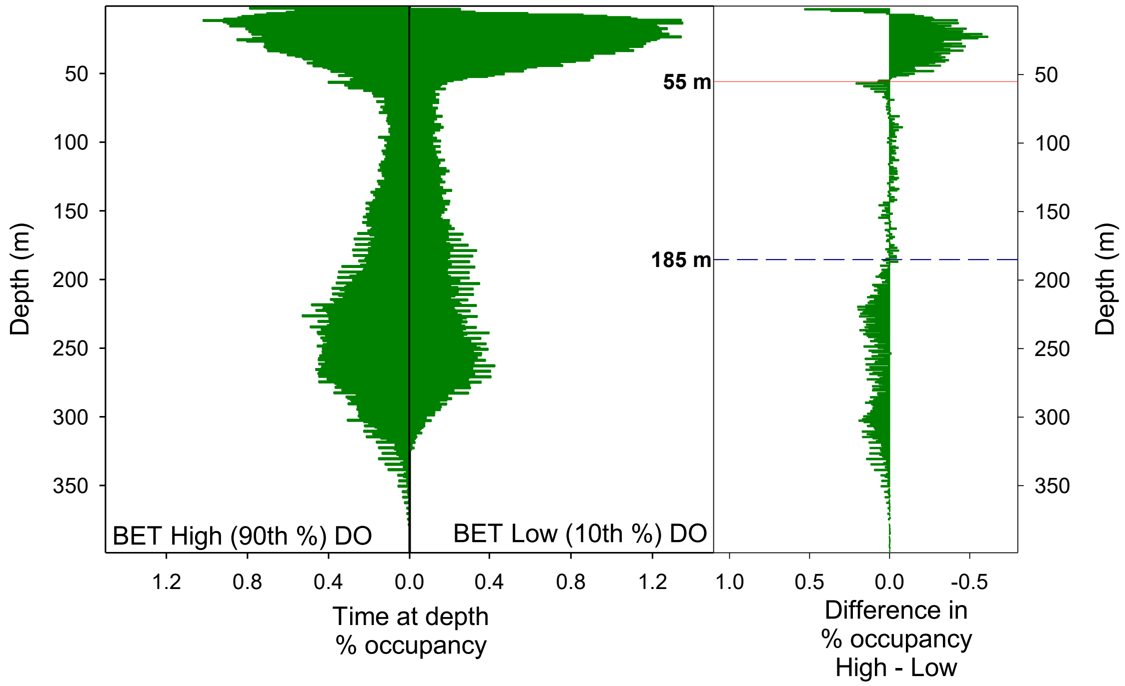

Figure S3: Comparative TAD plots with and without FAD associated days

The upper plot shows the original analysis, the lower plot shows analysis with the FAD days removed.

We then re-computed the differences in DO and temperature at a range of depths between grid cells where time spent above the 55 m threshold was in the upper and lower 10th percentile. Importantly again, the overall pattern is conserved, with an even lower value for DO at 300 m.

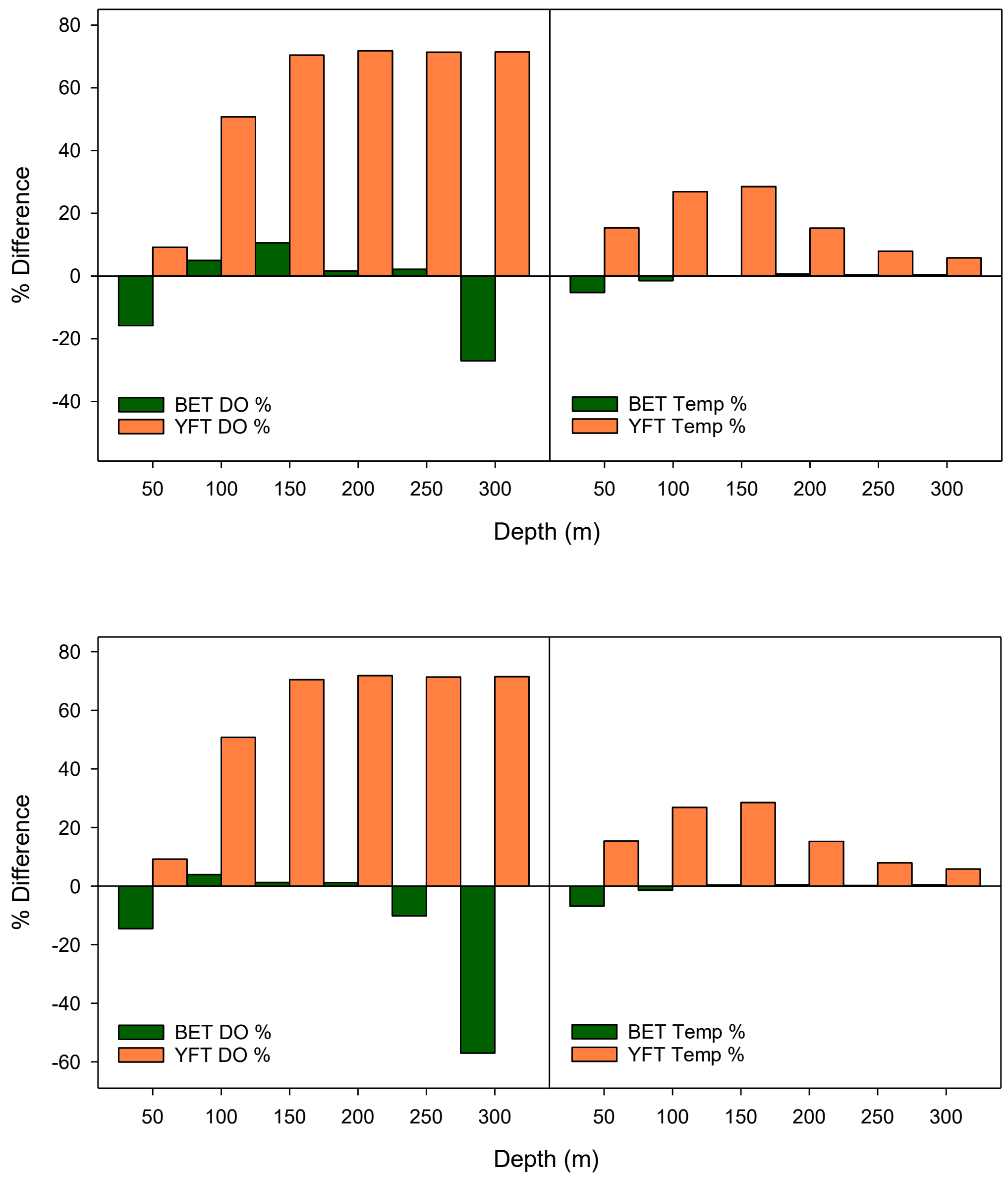

Figure S4: Differences in DO and temperature with and without FAD associated days

Upper plot shows the original analysis, lower plots shows analysis with FAD associated days removed. There is no significant difference in the overall pattern of response.

Finally, we reproduced the box plots of DO and temperature in the upper and lower 10th percentiles of time above 55 m. Here again, we find that the overall pattern is conserved.

We can conclude therefore that the removal of the 5 % of daytime locations, despite them being biased towards surface oriented behaviour, does not significantly affect the results or conclusions of the study. Consequently, there was no requirement to remove these locations and our contention that it is better to retain these locations still stands.

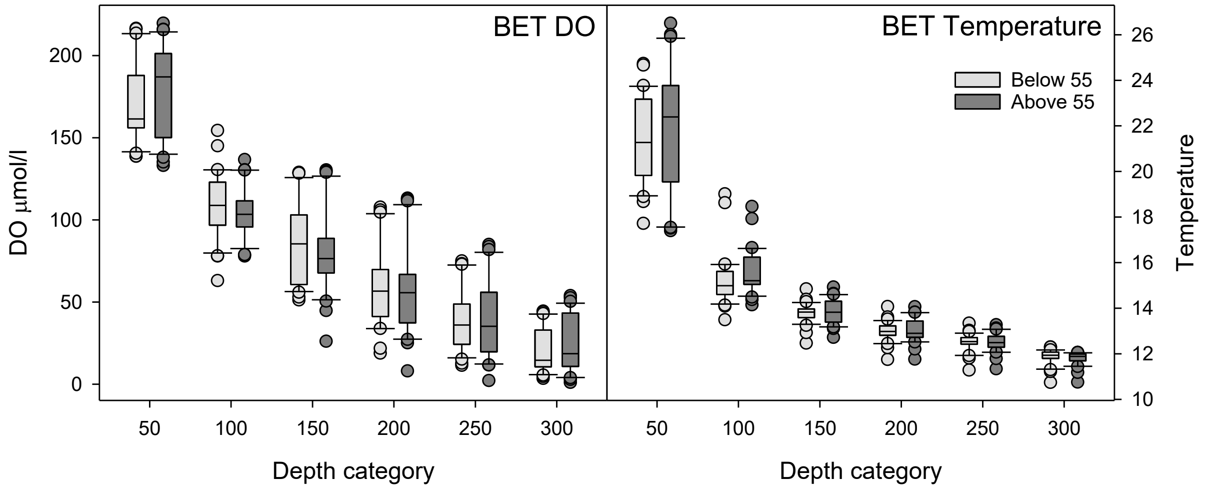

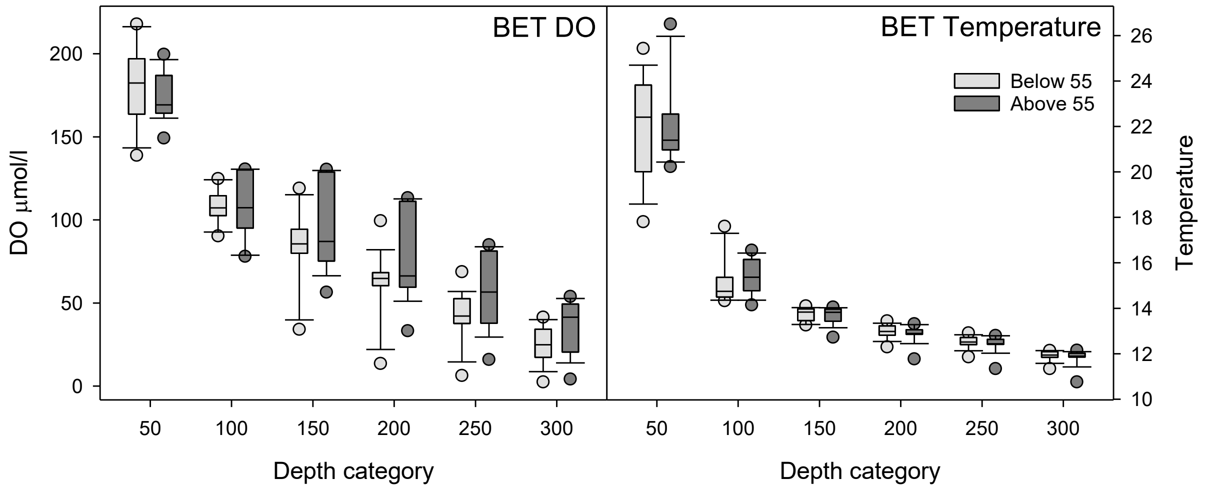

Figure S5: Box plots of DO and temperature above and below the behavioural depth thresholds

Upper plot shows the original analysis, lower plots shows analysis with FAD associated days removed. Box plots of DO and temperature above and below the behavioural depth thresholds. With the original data there are no significant differences in DO. When the FAD data are removed the differences are greater but not significant except for DO300. Temperatures are pretty much unchanged.

Responses to DO and temperature

Table S3: Differences in DO between areas where more time is spent above or below the threshold

The % of time is the median proportion of time spent above or below the threshold in the upper or lower 10th percentile of grid cells in that category. DO is shown at depths from 50 to 300 m. Differences are between the median values of the upper and lower 10th percentiles of grid cells. Statistical significance of differences was determined with a Mann-Whitney Rank Sum Test. Significant differences and correlations shown in **bold**.

BET	***% of time***	***DO50***	***DO100***	***DO150***	***DO200***	***DO250***	***DO300***
Below 55 m	78.05 %	161.47	108.86	85.48	56.65	36.07	14.64
Above 55 m	75.83 %	187.01	103.46	76.44	55.71	35.29	18.59
Difference		-25.54	5.40	9.04	0.94	0.78	-3.95
% Diff		-15.82	4.96	10.57	1.650	2.16	-27.01
p-value		0.330	0.330	0.541	0.596	0.952	0.448
Below 55 m vs		-0.0568	0.057	0.0731	0.072	0.0497	-0.002
p-value		0.313	0.312	0.194	0.201	0.378	0.972
YFT	***% of time***	***DO50***	***DO100***	***DO150***	***DO200***	***DO250***	***DO300***
Below 43 m	73.64 %	235.88	247.60	233.52	183.28	154.02	125.19
Above 43 m	94.41 %	214.22	121.99	69.00	51.66	44.13	35.73
Difference		21.66	125.61	164.52	131.62	109.89	89.46
% Diff		9.18	50.73	70.45	71.81	71.35	71.46
p-value		**0.013**	**<0.001**	**<0.001**	**<0.001**	**<0.001**	**<0.001**
Below 55 m vs		0.263	0.402	0.470	0.480	0.481	0.488
p-value		**< 0.001**	**< 0.001**	**< 0.001**	**< 0.001**	**< 0.001**	**< 0.001**

Table S4: Differences in temperature between areas where more time is spent above or below the threshold

The % of time is the median proportion of time spent above or below the threshold in the upper or lower 10th percentile of grid cells in that category. Median temperature is shown at depths from 50 to 300 m; Differences are between the median values of the upper and lower 10th percentiles of grid cells. Statistical significance of differences was determined with a Mann-Whitney Rank Sum Test. Significant differences and correlations shown in **bold**.

BET	***SST***	***Temp50***	***Temp100***	***Temp150***	***Temp200***	***Temp250***	***Temp300***
Below 55 m	25.11	21.28	14.99	13.83	12.98	12.53	11.94
Above 55 m	25.70	22.39	15.21	13.82	12.90	12.49	11.89
Difference	-0.59	-1.12	-0.22	0.01	0.08	0.04	0.05
% Diff	-2.35	-5.25	-1.48	0.07	0.64	0.35	0.46
p-value	0.069	0.274	0.061	0.941	0.587	0.846	0.095
Below 55 m vs	-0.154	-0.0113	-0.0269	-0.103	-0.118	-0.113	-0.154
p-value	0.006	0.841	0.634	0.066	0.035	0.044	0.006
YFT	***SST***	***Temp50***	***Temp100***	***Temp150***	***Temp200***	***Temp250***	***Temp300***
Below 43 m	20.51	20.06	18.13	14.87	11.01	9.77	8.96
Above 43 m	18.06	16.97	13.26	10.63	9.33	8.99	8.44
Difference	2.44	3.08	4.87	4.24	1.68	0.77	0.52
% Diff	11.92	15.37	26.86	28.51	15.27	7.92	5.80
p-value	0.393	**<0.001**	**<0.001**	**<0.001**	**0.045**	0.207	0.342
Below 43 m vs	-0.117	0.100	0.540	0.532	0.086	-0.060	-0.090
p-value	0.454	**0.039**	**<0.001**	**<0.001**	0.074	0.212	0.063

Table S5: Differences in depths between areas where more time is spent above or below the threshold

The % of time is the median proportion of time spent above or below the threshold in the upper or lower 10th percentile of grid cells in that category. MLD is mixed layer depth. Differences are between the median values of the upper and lower 10th percentiles of grid cells. Statistical significance of differences was determined with a Mann-Whitney Rank Sum Test. Significant differences and correlations shown in **bold**.

BET	***Bathymetric depth***	***MLD***	***Median depth***	***Max depth***
Below 55 m	3513.00	21.00	173.87	806.00
Above 55 m	3638.77	20.05	41.85	289.50
Difference	-125.76	0.95	132.03	516.50
% Diff	-3.58	4.54	75.93	64.08
p-value	**0.041**	0.653	**0.001**	**0.001**
Below 55 m vs	0.0586	0.0892	0.700	0.227
p-value	0.299	0.113	**<0.001**	**<0.001**
YFT	***Bathymetric depth***	***MLD***	***Median depth***	***Max depth***
Below 43 m	4409.63	59.49	62.78	182.00
Above 43 m	3076.26	19.41	16.13	216.00
Difference	1333.37	40.08	46.65	-34.00
% Diff	30.24	67.37	74.31	-18.68
p-value	**< 0.001**	**< 0.001**	**<0.001**	0.238
Below 43 m vs	0.445	0.605	0.929	-0.083
p-value	**< 0.001**	**< 0.001**	**< 0.001**	0.084

Table S6: Correlation coefficients for median depth

Significant correlations shown in **bold**

BET	***Bathymetric depth***	***DO50***	***DO100***	***DO150***	***DO200***	***DO250***	***DO300***
Median depth vs	-0.006	-0.080	0.012	0.054	0.058	-0.006	-0.080
p-value	0.911	0.154	0.838	0.339	0.300	0.911	0.154
YFT	***Bathymetric depth***	***DO50***	***DO100***	***DO150***	***DO200***	***DO250***	***DO300***
Median depth vs	0.393	0.232	0.392	0.450	0.451	0.393	0.232
p-value	**< 0.001**	**< 0.001**	**< 0.001**	**< 0.001**	**< 0.001**	**< 0.001**	**< 0.001**

BET Vertical excursions in relation to DO

Table S7: Differences in BET vertical excursions

The table shows differences in the median number of vertical excursions per track observed between areas in the upper (high) or lower (low) percentiles of grid cells based on DO at 300 m. Comparisons are shown between DO percentiles of 90-10 to 60-40. Significantly more vertical excursions occur in low DO areas in all comparisons. No significant differences in temperature were found. Significant p-values shown in **bold**.

DO difference	*Low DO excursions*	*High DO excursions*	*p*	*Low DO temperature*	*High DO temperature*	*p*
90-10	41.25	19.33	**0.005**	19.61	20.91	0.374
80-20	40.50	25.20	**0.003**	19.38	19.12	0.292
70-30	43.00	29.07	**0.003**	19.22	19.50	0.831
60-40	40.00	30.00	**0.006**	19.13	19.18	0.951

BET Vertical excursions in relation to body length

It is hypothesised that the number of vertical excursions performed is lower in larger (higher body mass) individuals, as a result of greater thermal inertia and therefore tolerance to lower temperatures. To test this, a scatter plot was produced of body (fork) length and average number of vertical excursions (Figure S2). Linear regression revealed a negative correlation with r2 = -0.503, slope = -0.009, supporting the hypothesis. However, it is clear from the plot that there are two distinct clusters, with all points in the lower right of the figure belonging to the cohort of BET tagged in 2000. To test whether the lengths of these individuals represented a significantly different cohort, the length distributions of the two sets of BET were compared using a Mann-Whitney rank sum test (Table S8). The results, shown below, confirm that the data represent two distinct cohorts ($p < 0.001$), which should be analysed separately.

The scatter plot in Figure S3 shows that when analysed separately, the two groups have a similar, positive correlation between body size and the number of vertical excursions ($r^2 = 0.351$, slope = 0.004 for the BET tagged between 2003-2005; $r^2 = 0.356$, slope = 0.003 for those tagged in 2000).

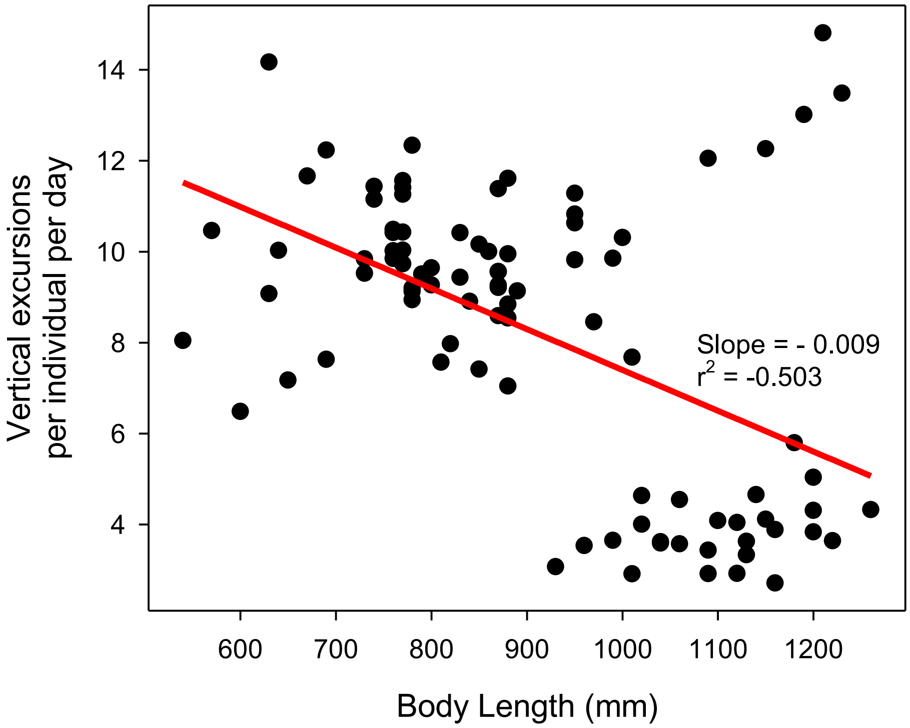

Figure S6: Body length v number of vertical excursions

The negative correlation appears to support the hypothesis that the number of vertical excursions decreases with body size, however, the plot reveals two distinct clusters of individuals.

Table S8: Mann-Whitney Rank Sum Test on body length

Mann-Whitney U Statistic= 117.500; Yates continuity correction option applied to calculations; T = 1988.500 n(small)= 27 n(big)= 64 (P = <0.001)

**Group**	**N**	**Median**	**25%**	**75%**
BET 2003-2005	64	815	760	880
BET 2000	27	1120	1040	1160

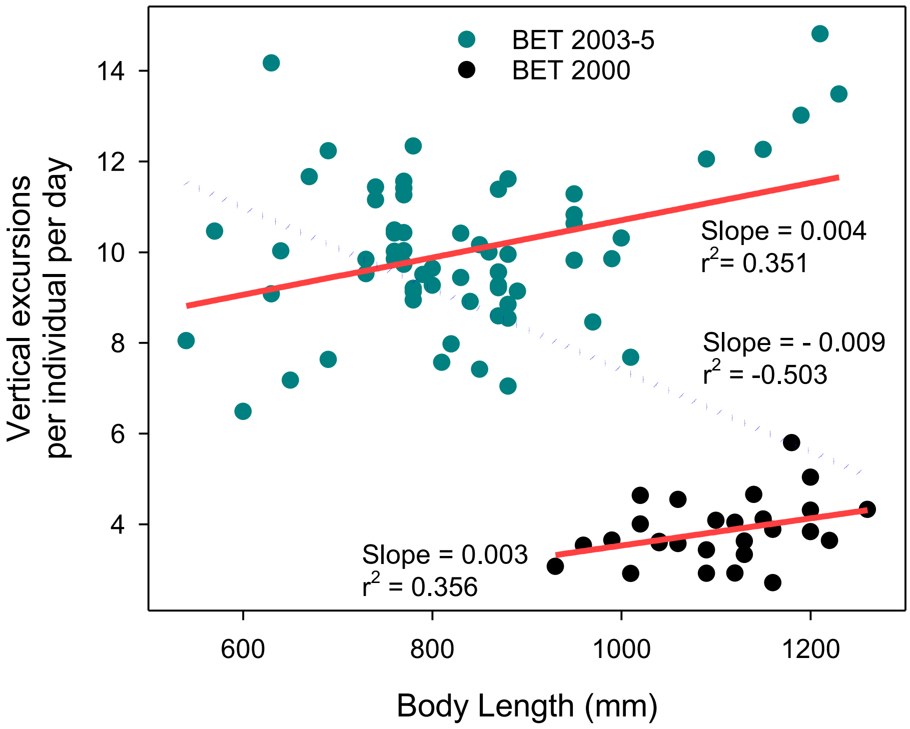

Figure S7: Body length v vertical excursions analysed separately for the two cohorts

When analysed as separate cohorts, both cohorts show similar, positive correlations. The dotted blue line shows the correlation when both cohorts are treated as one.

Results of checking the vertical excursion/length relationship

The original analysis used the beginning of track lengths. As the end of track lengths might differ this relationship might not hold if end of track lengths are used for the analysis. End of track lengths were used to repeat the analysis and confirmed that two clear cohorts of fish sizes were found. There were no differences in the results. This is not surprising considering the very small increase in mean length from 912.53 to 913 cm.

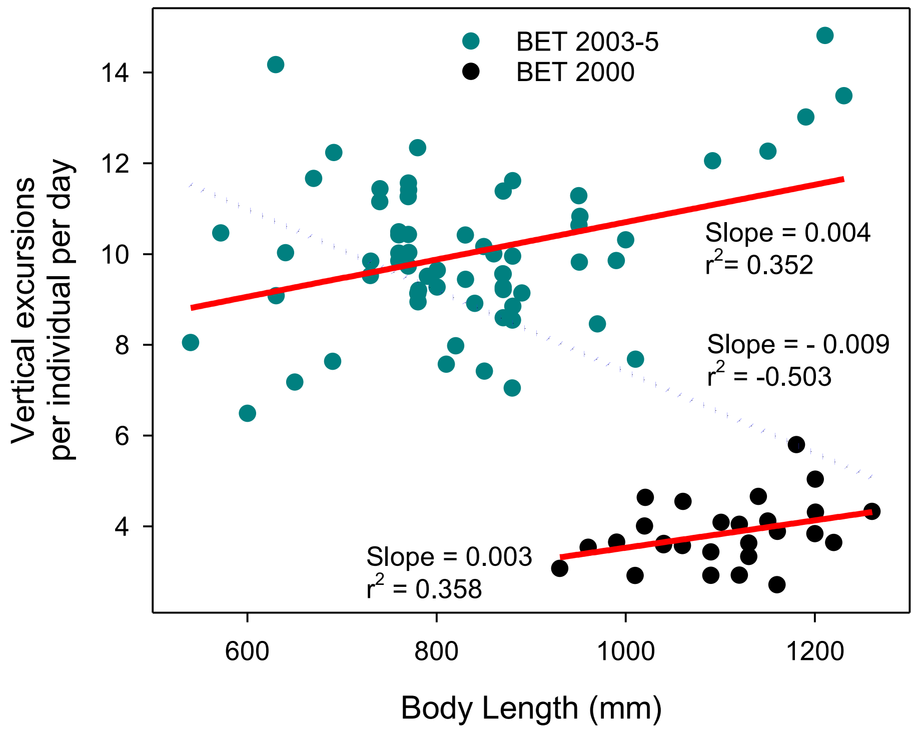

Figure S8: Reanalysis of body length vs vertical excursions analysis using end of track lengths

Analysis of the occurrence of exceptional deep dives.

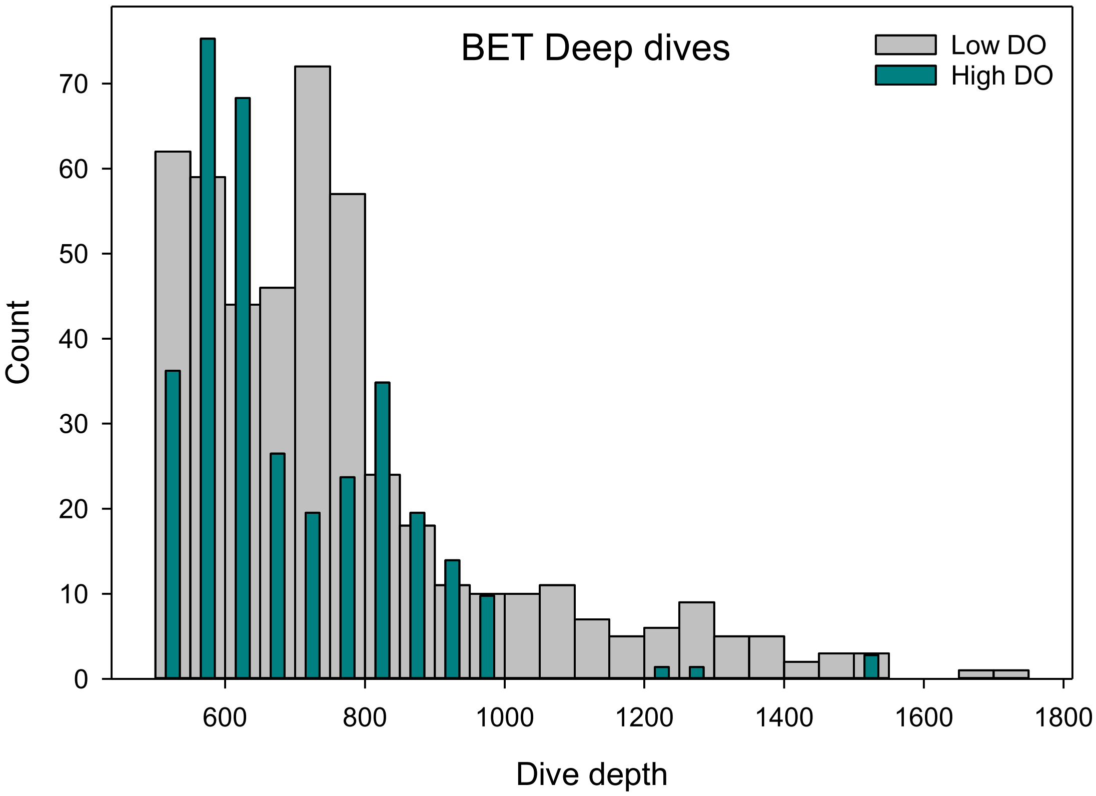

Figure S9: Histogram showing the distribution of deep dives for BET
More deeper dives are observed in areas where DO is low

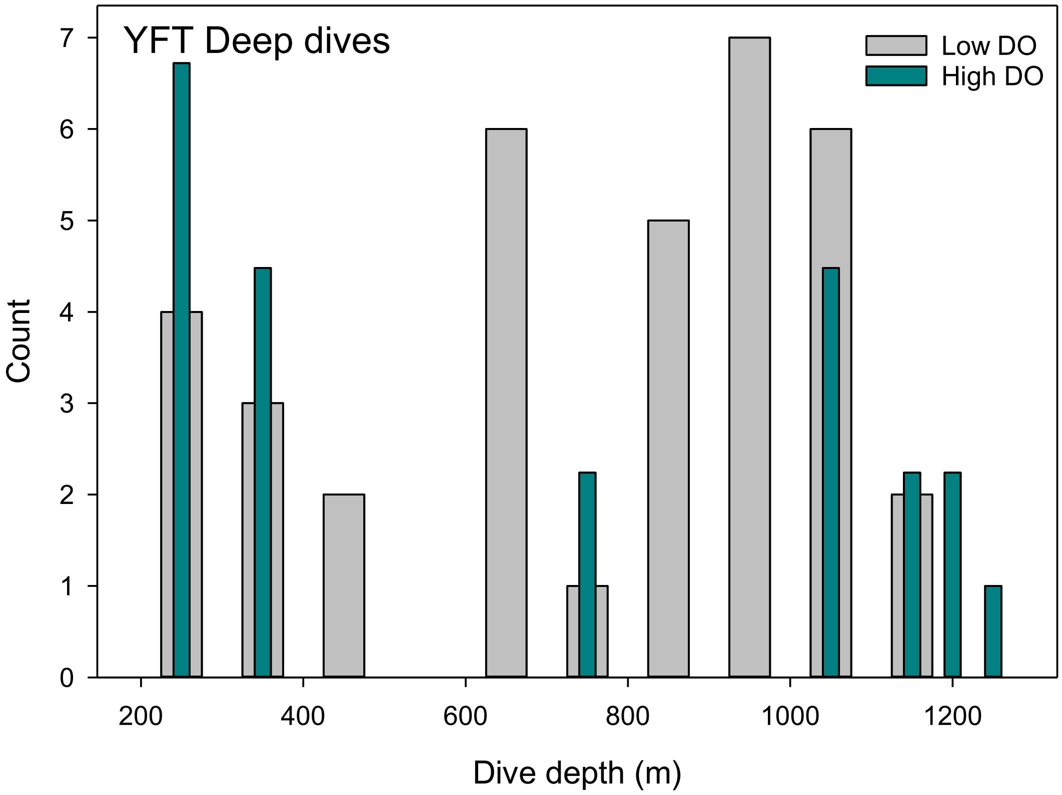

Figure S10: Histogram showing the distribution of deep dives for YFT

Table S9: Deep dives in low and high DO grid cells

	BET			YFT		
	**Count**	**Max depth**	**Median depth**	**Count**	**Max depth**	**Median depth**
Low DO	471	1706	806.0	36	1118	910.0
High DO	239	1530	289.5	11	1204	1129.0

Table S10: DO concentrations (umol/l) for deep dives in low and high DO regions

DO is significantly lower for deep dives performed in low DO regions (Mann-Whitney Rank Sum test, p = 0.017), which is expected, given that the dives are significantly deeper.

	No. of dives	Median DO	25% DO	75% DO
Low O2	471	37.044	28.819	53.547
High O2	239	43.251	32.548	49.836

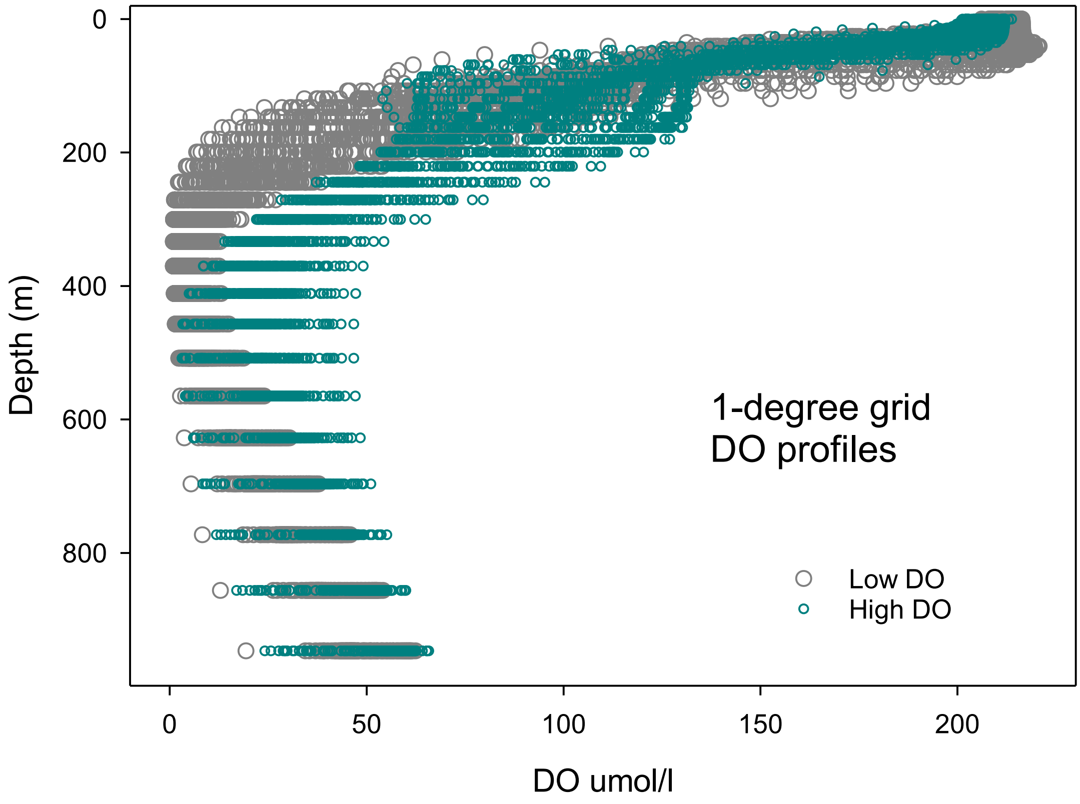

Figure S11: DO Profiles for the 1-degree grid cells in the upper and lower percentiles of DO at 300 m

The grid cells selected for the BET analysis represented the 10th and 90th percentiles of DO at 300, and the differences in DO at depths around 300 m are clear. However, below 400 m there is very little difference in DO concentration.

Analysis of lengths of BET and YFT individuals

We found that the lengths of BET were slightly but significantly greater than YFT (Mann-Whitney Rank Sum Test, p = 0.011).

Table S11: Lengths of BET and YFT in this study

Species	N	Median	25%	75%
YFT	175	820	710	980
BET	92	880	770	1060

Analysis at 185 m threshold for BET

Table S12: Differences in DO between areas where more time is spent above or below the threshold

The % of time is the median proportion of time spent above or below the threshold in the upper or lower 10th percentile of grid cells in that category. DO is shown at depths from 50 to 300 m. Differences are between the median values of the upper and lower 10th percentiles of grid cells. Statistical significance of differences was determined with a Mann-Whitney Rank Sum Test. Significant differences and correlations shown in **bold**.

	**% of time**	**DO50**	**DO100**	**DO150**	**DO200**	**DO250**	**DO300**
Below 185	56.75%	161.13	109.95	80.90	56.26	31.67	13.18
Above 185	97.63%	185.13	103.05	70.12	53.13	34.92	18.60
Difference		-24.00	6.90	10.77	3.13	-3.25	-5.41
% Diff		-14.90	6.28	13.32	5.57	-10.25	-41.05
p-value		0.268	0.481	0.245	0.559	0.773	0.251
Below 55 m vs		-0.15	0.14	0.20	0.15	0.06	-0.06
p-value		**<0.001**	0.013	**<0.001**	**0.008**	0.277	0.299

Table S13: Differences in temperature between areas where more time is spent above or below the threshold

The % of time is the median proportion of time spent above or below the threshold in the upper or lower 10th percentile of grid cells in that category. Median temperature is shown at depths from 50 to 300 m; Differences are between the median values of the upper and lower 10th percentiles of grid cells. Statistical significance of differences was determined with a Mann-Whitney Rank Sum Test. Significant differences and correlations shown in **bold**.

	**SST**	**Temp50**	**Temp100**	**Temp150**	**Temp200**	**Temp250**	**Temp300**
Below 185	24.28	20.67	14.91	13.86	13.09	12.58	11.98
Above 185	25.37	22.09	15.37	13.59	12.87	12.44	11.82
Difference	-1.09	-1.41	-0.46	0.27	0.21	0.14	0.16
% Diff	-4.51	-6.83	-3.07	1.92	1.64	1.13	1.30
p-value	**0.006**	**0.037**	**0.049**	0.235	**0.011**	**0.011**	**<0.001**
Below 55 m vs	-0.28	-0.19	-0.08	0.13	0.18	0.20	0.27
p-value	**<0.001**	**<0.001**	0.179	**0.017**	**0.002**	**<0.001**	**<0.001**

Table S14: Differences in depths between areas where more time is spent above or below the threshold

The % of time is the median proportion of time spent above or below the threshold in the upper or lower 10th percentile of grid cells in that category. MLD is mixed layer depth. Differences are between the median values of the upper and lower 10th percentiles of grid cells. Statistical significance of differences was determined with a Mann-Whitney Rank Sum Test. Significant differences and correlations shown in **bold**.

	Median depth	Max Depth	Vertical Excursions	Bathymetric depth
Below 185	170.91	911.50	61.38	3491.73
Above 185	46.61	260.50	7.50	3694.70
Difference	124.30	651.00	53.88	-202.97
% Diff	72.73	71.42	87.78	-5.81
p-value	**<0.001**	**<0.001**	**<0.001**	**0.005**
Below 55 m vs	0.77	0.35	0.15	-0.09
p-value	**<0.001**	**<0.001**	**0.008**	0.097

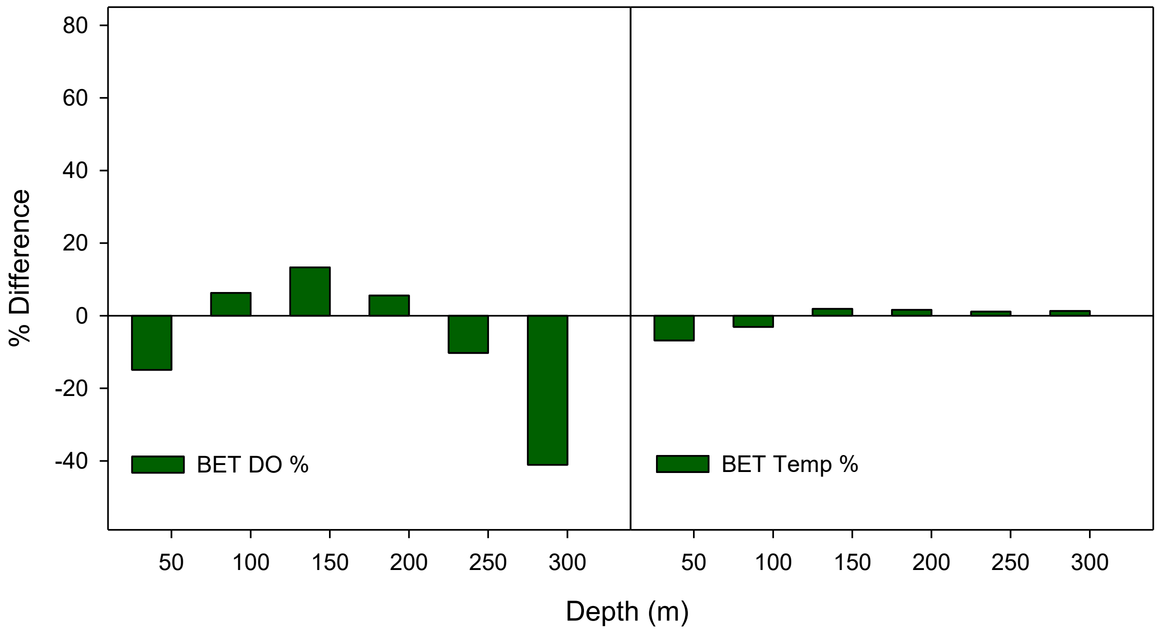

Figure S12: Analysis of differences in DO and temperature above and below the 185 m depth threshold

Difference in DO concentration and temperature between the upper and lower 10 % of 1-degree grid cells based on time spent below the depth thresholds of 185 m (BET).

GAM Analysis

Summary

In the BET models for maximum daily depth and average (median) daily depth, no model explained more than 8 % of the deviance, except for the models incorporating fork length or track ID, which

explained 2.65 and 8.5 % respectively for maximum depth, 13.7 and 21.5 % for average depth and 17.5 and 23.7 % for the number of vertical excursions.

In the YFT models for maximum daily depth, no model other than the one including track, accounted for more than 6 % of the deviance, suggesting that none of the models offered a useful predictor for the response variable. For average depth, however, Temp100 and SST were found to explain 11.0 and 7.34 % of the deviance respectively and in combination to explain 12.8 % which suggests that temperature may be an important factor in the average depth. Nonetheless, the model with track ID as a random factor still explained almost twice the deviance (24.1 %) and the response graphs show that the effect of increasing temperature at 100 m, or SST is complex (Supplementary Figure S16) and neither provided a straightforward predictor of average depth.

With both BET and YFT we found that fish fork length explained more deviance than any factor other than Track ID as a random factor. To investigate whether the fork length (i.e. body mass) of the fish was itself a significant factor we constructed models to allow fork length and track to be compared (e.g. `MaximumDepth ~ s(Length) + s(Track, bs="re")`). In all cases, we found that the combination of track and body length explained no more deviance than track alone and graphs of length as a factor showed no clear relationship (e.g. Supplementary Figure S9, Figure S11 and Figure S14). These results confirmed our hypothesis that fork length, like Track ID, was essentially a random factor contributing to the overall effect of individual variation in the data.

The models

A number of models were tested, in the form:

```
Response ~ s(DO)
Response ~ s(Temp)
Response ~ s(SST)
Response ~ s(DO) + s(Temp)
Response ~ s(DO) + s(SST)
Response ~ s(Temp) + s(SST)
Response ~ s(DO) + s(Temp) + s(SST)
Response ~ s(Length)                For time series analyses only
Response ~ s(Track, bs=”re”)        For time series analyses only
```

GAM Analysis of detailed BET data

The analysis presented here utilised the detailed (individual) depth time-series location data, as opposed to the summarised spatial data derived from the analysis of 1 degree grid cells, which is presented later. The analysis performed on the detailed data was less extensive than that perform on the spatial data, because it became clear that track (as a random factor) or fish length (for vertical

excursions), explained more deviance than any of the environmental factors. Individual variation therefore confounded the results.

BET vertical excursions

The detailed vertical excursion analysis uses counts of vertical excursions calculated each day for each shark. Vertical excursions were computed from the merged location data, which includes depths from the depth time series data, together with the daily locations from the geo-located position data. Step lengths in the depth component were computed as the vertical distances between reversals in the direction of movement, summing consecutive move steps. A computed move step that was >= 100 m was counted as half a vertical excursion, as a complete excursion involves movement to and from the surface. Only movements between the daytime hours of 06:00 to 17:00 (local time) were counted, although movements greater than 100 m during night-time hours were very rare.

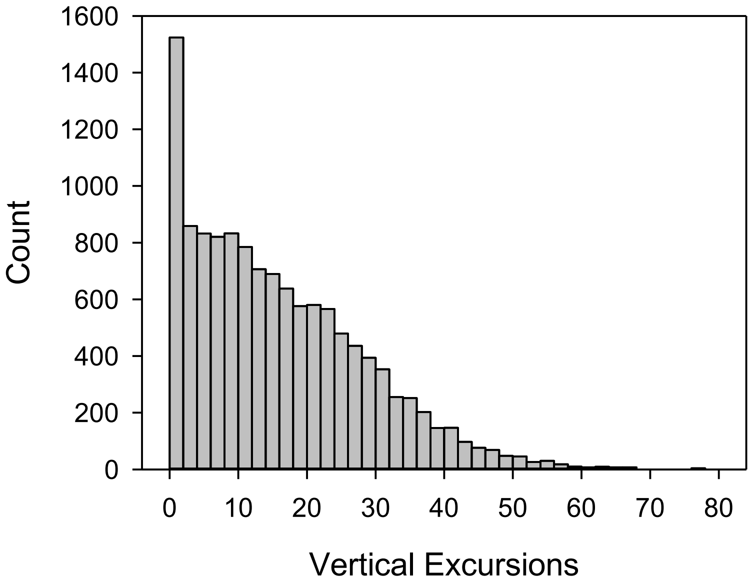

Figure S13: Histogram showing the distribution of vertical excursion counts for BET

Using a simple model, we determined the best distribution for the number of vertical excursions to be Inverse Gaussian:

```
gam(VerticalExcursions ~ + s(DO300), family=inverse.gaussian(link = "1/mu^2") ,
      data=BETVEDetail, method = 'REML', select = TRUE)
```

Table S15: Models to determine the best fit distribution for BET vertical excursion counts
The best fit model shown in **bold**

Distribution	df	AIC	wAIC
Gaussian	8.803641	70625.82	0.000
Gaussian (link = log)	8.818296	70626.67	0.000
Inverse Gaussian (link = 1/mu^2)	6.459414	69073.72	0.000
Gamma (link = inverse)	8.735477	67397.93	0.287
**Gamma (link = identity)**	**8.713982**	**67396.11**	**0.713**
Poisson	9.952434	85960.65	0.000

Table S16: GAM outputs showing track as a random factor explains the most deviance

Model	df	AIC	wAIC	% Deviance
s(DO300)	9.2996	85420.90	0.0000	1.12
s(Temp300)	9.4974	85423.14	0.0000	1.11
s(SST)	10.9430	85384.31	0.0000	1.44
s(DO300) + s(Temp300)	17.1361	85270.54	0.0000	2.12
s(DO300) + s(SST)	17.0127	85309.43	0.0000	2.43
s(Temp300) + s(SST)	17.5633	85275.33	0.0000	2.40
s(DO300) + s(Temp300) + s(SST)	25.7881	85128.60	0.0000	3.67
s(Length)	9.9670	83183.66	0.0000	17.50
**s(Track, bs="re")**	**88.5055**	**82376.10**	**1.0000**	**23.70**

The model that included Track as a random factor, to account for individual variation explained considerably more deviance and therefore we concluded that individual variation has a greater effect than the environmental variables. Length accounted for 17.5% of the deviance and possibly contributed to the high deviance explained by Track. No other model accounted for more than 3.67% of the deviance.

To better understand the effects of length and track id and investigate the possibility that the deviance explained by length was confounded with the individual variation explained by track as a random factor, separate models were tested using track and length as shown in Table S17. Length and track together explain slightly less deviance than track as a random factor, which suggests that both track and length are expressing the individual variation in the data.

Table S17: GAM outputs comparing length with Track as a random factor

Model	df	AIC	wAIC	% Deviance
s(Length)	9.9670	83183.66	0.0000	17.50
**s(Track, bs="re")**	**88.5055**	**82376.10**	**0.5283**	**23.70**
s(Length) + s(Track, bs="re")	82.7158	82376.33	0.4717	23.60

The GAM plot of length (Figure S9) confirms that there is no clear relationship between length and the number of vertical excursions observed.

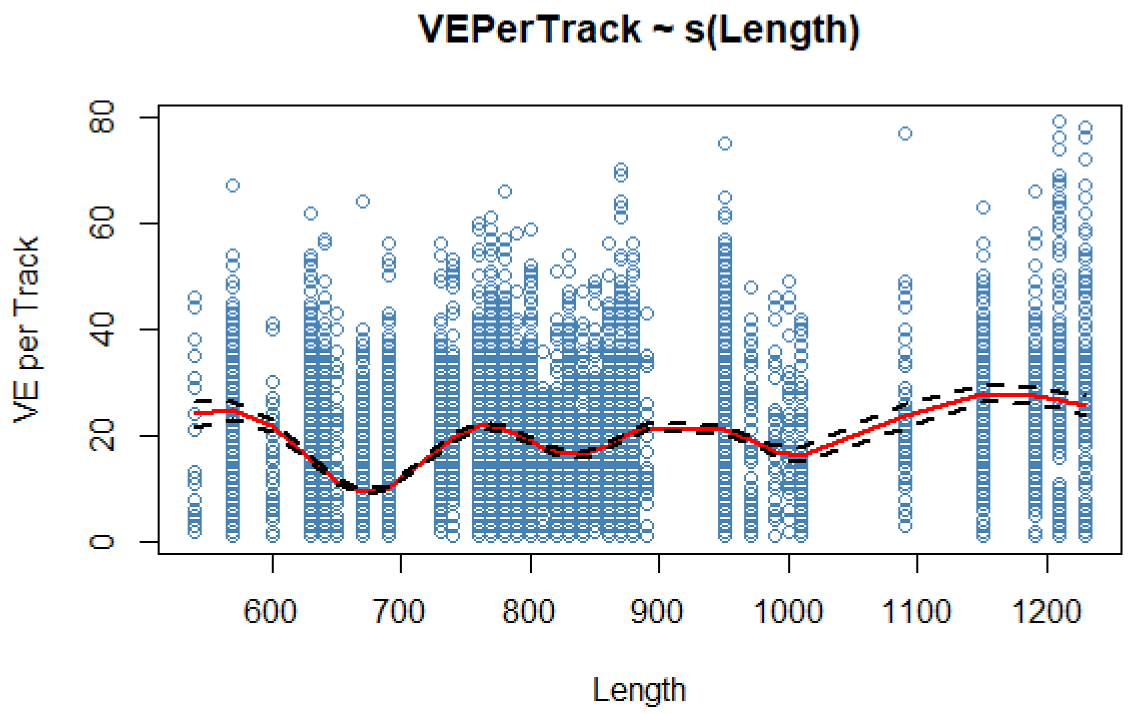

Figure S14: GAM plot of the effect of length on the number of vertical excursions
There is no clear relationship between length and vertical excursions

BET maximum daily depths

For each individual we computed the maximum daily depth; the occasional very deep dives, to below 500 m for which there is still no clear explanation, were not considered to be part of the normal daily diving behaviour and were therefore excluded.

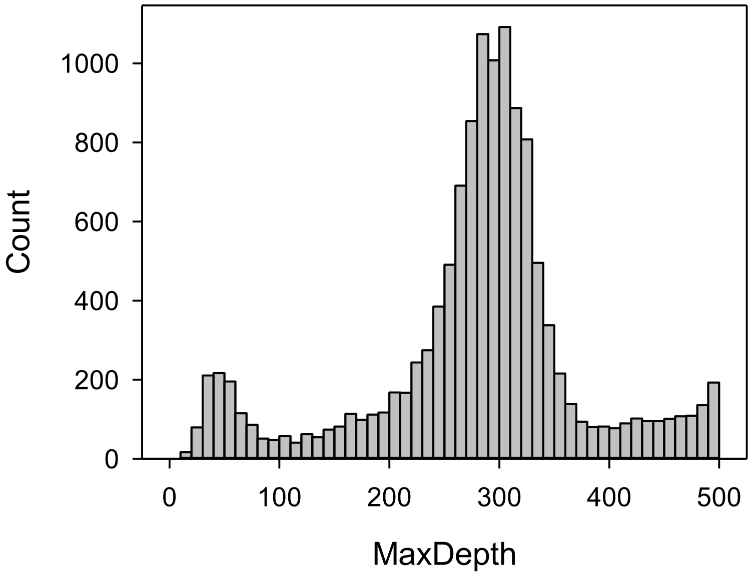

Figure S15: Histogram showing the distribution of maximum daily depth counts for BET

Again, using a simple model, we first determine the best fit distribution for the data, which in this case is Gaussian.

Table S18: Models to determine the best fit distribution for BET maximum daily depth counts
The best fit model shown in **bold**

Model	df	AIC	wAIC
**Gaussian**	**6.4373**	**150166.40**	**0.5000**
Gaussian (link = log)	6.3923	150166.40	0.5000
Inverse Gaussian (link = 1/mu^2)	4.1911	162050.50	0.0000
Gamma (link = inverse)	5.0240	155208.40	0.0000
Gamma (link = identity)	5.0123	155208.60	0.0000
Poisson	9.9839	599480.40	0.0000

As with vertical excursions, we test the hypothesis that maximum daily depth is driven by DO or temperature at depth (300 m) or by SST. Again, we include Track as a random factor to account for individual variation in a null model.

Table S19: GAM outputs showing track as a random factor explains the most deviance

Model	Df	AIC	wAIC	% Deviance
s(DO300)	6.4373	150166.40	0.0000	0.42
s(Temp300)	8.3096	150139.30	0.0000	0.66
s(SST)	10.5838	150092.90	0.0000	1.07
s(DO300) + s(Temp300)	13.2772	150102.70	0.0000	1.03
s(DO300) + s(SST)	14.8599	150035.10	0.0000	1.59
s(Temp300) + s(SST)	17.0383	149980.30	0.0000	2.05
s(DO300) + s(Temp300) + s(SST)	21.3236	149947.60	0.0000	2.37
s(Length)	9.7940	149835.30	0.0000	2.65
**s(Track, bs="re")**	**85.5514**	**149251.20**	**1.0000**	**8.51**

No model explained more than 2.37 % of the deviance, except the model that included Length (2.65 %), or the model with Track as a random factor, which itself only explained 8.51 %. Consequently, we concluded that individual variation has a greater effect than the environmental variables, and that only part of that effect was due to the length.

As with the vertical excursion analysis, we tested length and track separately to determine whether length contributed significantly to the maximum depths or was simply part of the individual variation encompassed by track.

Table S20: GAM outputs comparing length with Track as a random factor

Model	df	AIC	wAIC	% Deviance
s(Length)	9.7940	149835.30	0.0000	2.65
**s(Track, bs="re")**	**85.4859**	**149208.10**	**0.4378**	**8.51**
s(Length) + s(Track, bs="re")	84.4701	149207.60	0.5622	8.50

The GAM plot of length (Figure S9) confirms that there is no clear relationship between length and the number of vertical excursions observed.

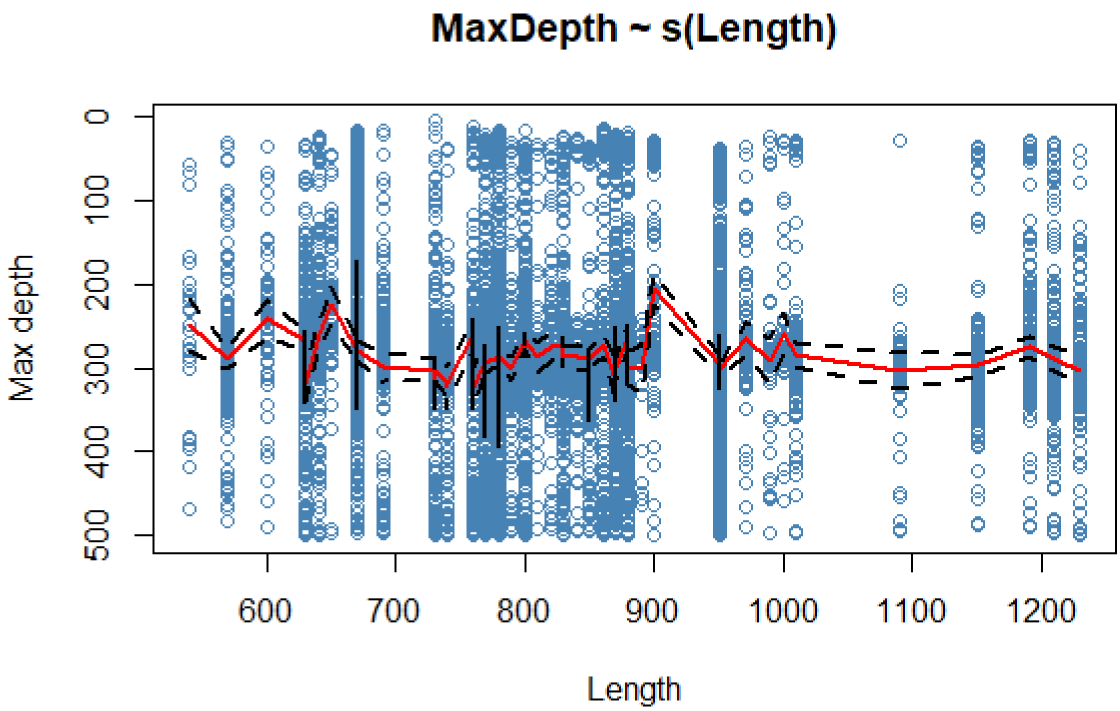

Figure S16: GAM plot of the effect of length on the maximum daily depth
There is no clear relationship between length and maximum depth

BET average daily depth

For each individual we computed the average daily depth between the hours of 06:00 and 17:00 local time; again the occasional very deep dives were not considered to be part of the normal daily diving behaviour and were therefore excluded.

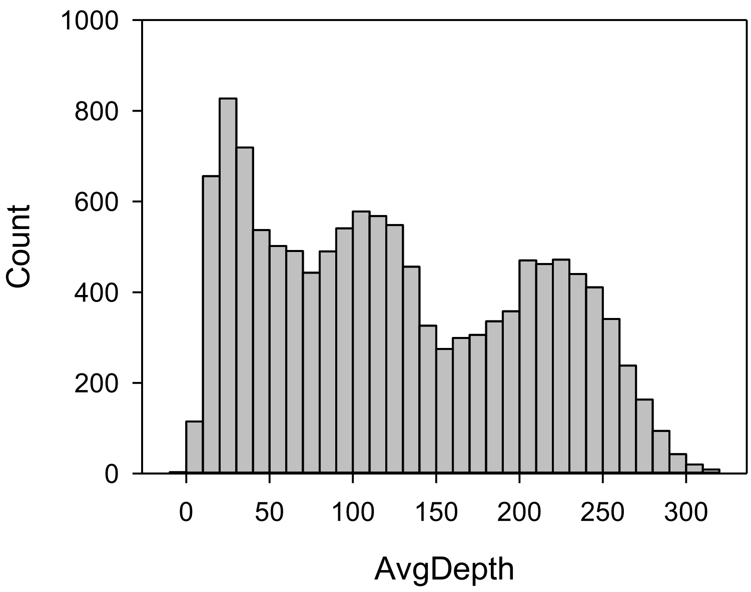

Figure S17: Histogram showing the distribution of average daily depth counts for BET

Again, using a simple model, we first determine the best fit distribution for the data, which in this case is Gaussian with link=log. In this case, given the form of the histogram, it is likely that Gaussian is simply the least bad fitting distribution of those tested.

Table S21: Models to determine the best fit distribution for BET average daily depth counts
The best fit model shown in **bold**

Distribution	df	AIC	wAIC
Gaussian	9.7428	145263.90	0.3540
**Gaussian (link = log)**	**9.5182**	**145262.70**	**0.6460**
Inverse Gaussian (link = 1/mu^2)	4.1911	162050.50	0.0000
Gamma (link = log)	5.0240	155208.40	0.0000
Gamma (link = identity)	5.0123	155208.60	0.0000
Poisson	9.9839	599480.40	0.0000

As with vertical excursions, we test the hypothesis that average daily depth is driven by DO or temperature at depth (300 m) or by SST, including Track as a random factor to account for individual variation in a null model.

Table S22: GAM outputs showing track as a random factor explains the most deviance

Model	Df	AIC	wAIC	% Deviance
s(DO300)	9.0754	144740.00	0.0000	5.01
s(Temp300)	9.4192	145044.70	0.0000	2.67
s(SST)	9.7175	145135.10	0.0000	1.97
s(DO300) + s(Temp300)	16.8581	144500.70	0.0000	6.92
s(DO300) + s(SST)	16.3265	144626.10	0.0000	5.97
s(Temp300) + s(SST)	17.3728	144905.70	0.0000	3.87
s(DO300) + s(Temp300) + s(SST)	25.1367	144427.90	0.0000	7.58
s(Length)	9.8892	143544.90	0.0000	13.70
**s(Track, bs="re")**	**88.7126**	**142501.80**	**1.0000**	**21.50**

As with the previous two models, the inclusion of track as a random factor explains considerably more deviance, suggesting that individual variation is a more important factor than the environmental variables. However, in this case, length is a more important factor than with maximum depth, explaining 13.7 % of the deviance. Also, the model with DO300 + Temp300 and SST explains 7.58 % of the deviance. These results suggest that DO and temperature at depth have some effect on the average depth.

GAM Analysis of detailed YFT data

YFT max daily depth

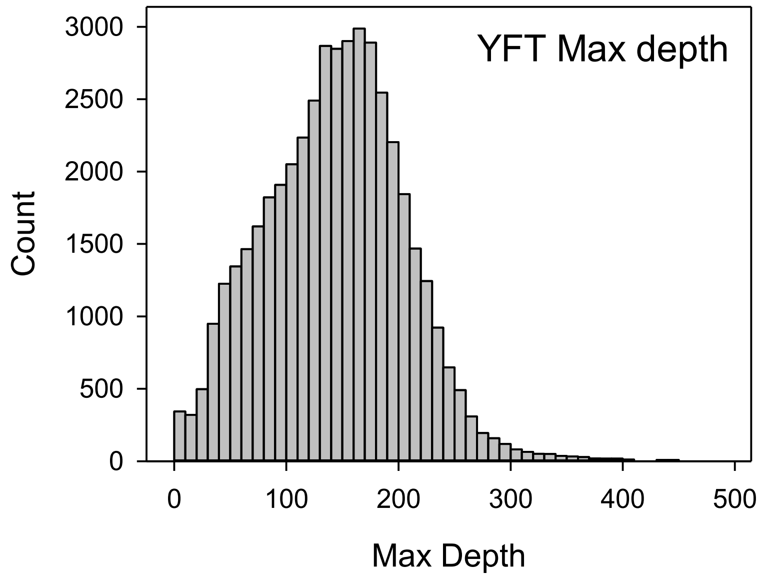

Figure S18: Histogram showing the distribution of maximum daily depth counts for YFT

Again, using a simple model, we first determine the best fit distribution for the data, which in this case is Gaussian.

Table S23: Models to determine the best fit distribution for YFT maximum daily depth counts
The best fit model shown in **bold**

Distribution	df	AIC	wAIC
**Gaussian**	**9.561053**	**503332.9**	**0.5351**
Gaussian (link = log)	9.54281	503333.2	0.4649
Inverse Gaussian (link = 1/mu^2)	8.231942	549119.5	0.0000
Gamma (link = log)	9.466314	509839.9	0.0000
Gamma (link = identity)	9.460227	509839	0.0000
Poisson	9.989236	1631819	0.0000

As with BET, the inclusion of track explains considerably more deviation than the environmental variables and length is less important.

Table S24: GAM outputs showing track as a random factor explains the most deviance

Model	df	AIC	wAIC	% Deviance
s(DO300)	9.5401	502644.50	0.0000	0.78
s(Temp300)	9.8863	500939.60	0.0000	4.50
s(SST)	10.7557	501834.20	0.0000	2.57
s(DO300) + s(Temp300)	17.3268	500603.60	0.0000	5.27
s(DO300) + s(SST)	18.4370	501125.40	0.0000	4.27
s(Temp300) + s(SST)	17.7220	500726.40	0.0000	4.96
s(DO300) + s(Temp300) + s(SST)	25.9653	500280.00	0.0000	6.00
s(Length)	10.9564	500527.00	0.0000	5.32
**s(Track, bs="re")**	**172.800914**	**495535.5**	**1.0000**	**15.8**

As with BET, separate models were tested using track and length as shown in Table S17. Length and track together explain the same deviance as just track as a random factor, which suggests that both track and length are expressing the individual variation in the data.

Table S25: GAM outputs comparing length with Track as a random factor

Model	df	AIC	wAIC	% Deviance
s(Length)	10.9564	500527.00	0.0000	5.32
**s(Track, bs="re")**	**172.8009**	**495535.50**	**0.5390**	**15.80**
s(Length) + s(Track, bs="re")	172.1164	495535.80	0.4610	15.80

The GAM plot of length (Figure S14) confirms that there is no clear relationship between length and the maximum daily depths.

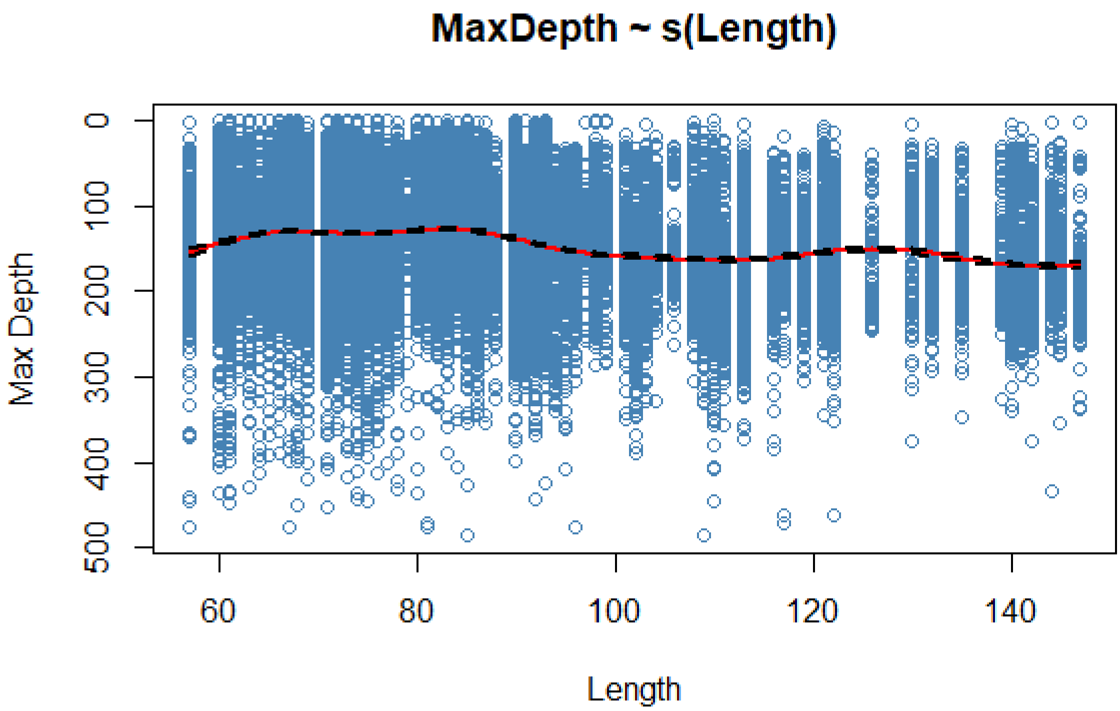

Figure S19: GAM plot of the effect of length on the maximum daily depth
There is no relationship between length and maximum depth

YFT Average daily depth

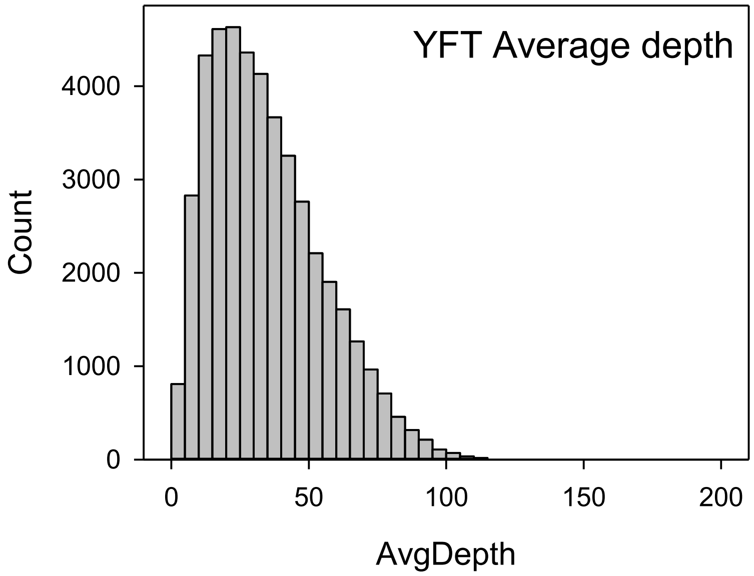

Figure S20: Histogram showing the distribution of average daily depth counts for YFT

The simple test model revealed Gamm with link=inverse to be the best fit distribution.

Table S26: Models to determine the best fit distribution for YFT average daily depth counts
The best fit model shown in **bold**

Distribution	df	AIC	wAIC
Gaussian	10.89258	401386.0	0.0000
Gaussian (link = log)	10.85067	401391.1	0.0000
Inverse Gaussian (link = 1/mu^2)	9.958553	406102.9	0.0000
**Gamma (link = inverse)**	**10.8481**	**393672.7**	**1.0000**
Gamma (link = identity)	9.940119	393758.5	0.0000
Poisson	9.98952	778080.4	0.0000

Once again, the inclusion of track to account for individual variation explains more of the deviance than any of the other models.

Table S27: GAM outputs showing track as a random factor explains the most deviance

Model	df	AIC	wAIC	% Deviance
s(DO300)	10.8402	393572.5	0.0000	0.99
s(Temp100)	10.9454	388430.2	0.0000	11.0
s(SST)	9.9259	390380.9	0.0000	7.34
s(DO300) + s(Temp100)	17.9321	388297.7	0.0000	11.2
s(DO300) + s(SST)	18.7588	389047.3	0.0000	9.91
s(Temp100) + s(SST)	17.8611	387465.3	0.0000	12.8
s(DO300) + s(Temp100) + s(SST)	26.6046	387126.9	0.0000	13.5
s(Length)	10.9564	500527.0	0.0000	11.3
s(Track, bs="re")	174.7985	381230.1	1.0000	24.1

Here, SST, temperature at 100 m and length have a greater effect on average depth than maximum depth. However, individual variation is still the dominant factor (24.1 %), with length (11.3 %) likely being part of this.

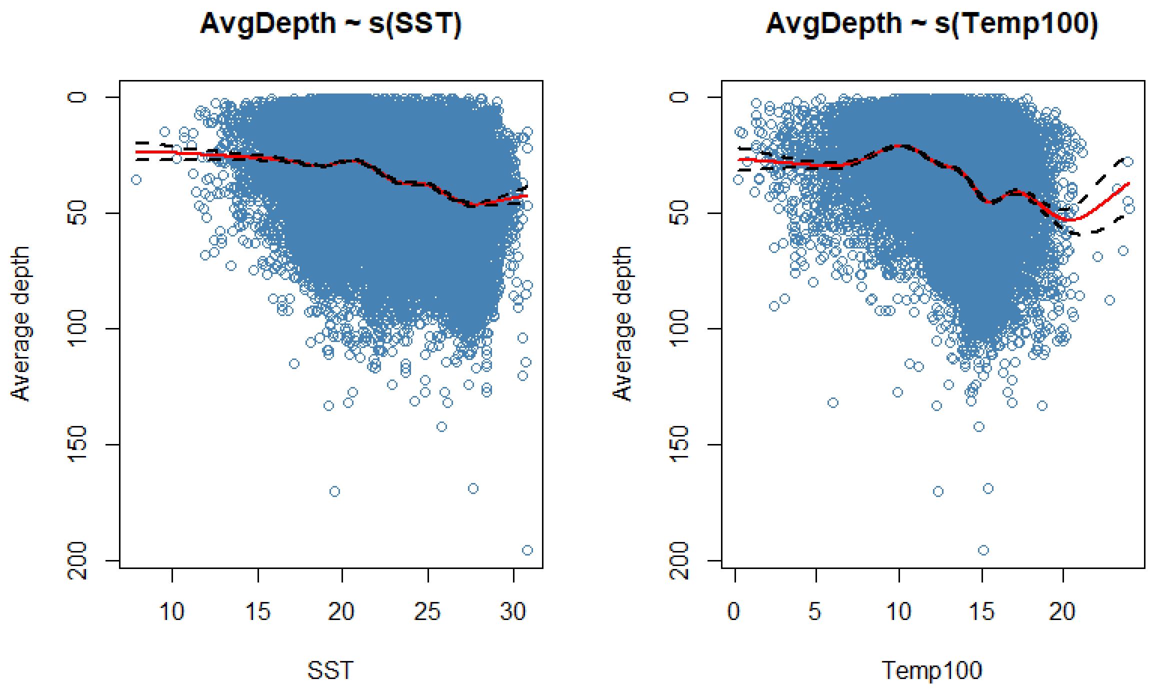

Figure S21: Plots of the variables explaining the most deviance for YFT average depth

Increasing SST and temperature at 100 m tends to increase the average depth, however this is not a simple linear relationship and is difficult to interpret.

GAM analysis of BET spatial data

This analysis is based on average values derived from all occupied 1x1 degree grid cells and is therefore a spatial analysis of occupancy of the water column that can be related to the averaged environmental variables in each location.

In this analysis we first tested a simple model (using just DO300) to determine which distribution best described the data. We then tested a further set of simple models, in the form

Below55 ~ s(DO300)

to determine which depth explained most deviation for DO and temperature. With these factors determined, we then tested models with the factors in combination to ascertain the most important drivers of behaviour (such as time spend below 55 m).

BET time below 55 m

Here we are using the GAM to relate the time spent below 55 m to SST, DO and temperature at depth in each occupied grid cell.

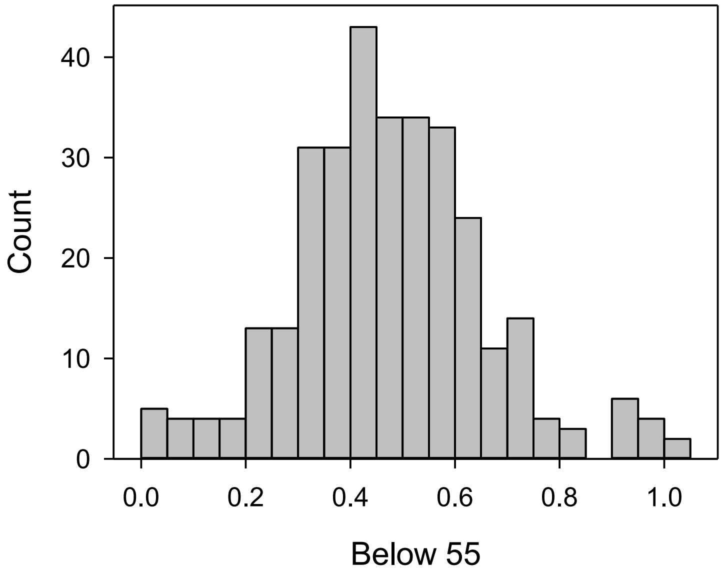

Figure S22: Histogram showing the distribution of time below 55 m proportions for BET

Again, using a simple model, we first determine the best fit distribution for the data, which in this case is Gaussian.

Table S28: Models to determine the best fit distribution for BET time below 55 m proportions
The best fit model shown in **bold**

Distribution	df	AIC	wAIC
**Gaussian**	**6.9073**	**2621.07**	**0.6415**
Gaussian (link = log)	6.7364	2622.31	0.3451
Inverse Gaussian (link = 1/mu^2)	2.0013	2719.05	0.0000
Gamma (link = log)	7.2135	2636.13	0.0003
Gamma (link = identity)	7.5396	2628.86	0.0131
Poisson	9.1066	3559.36	0.0000

Using this distribution, we test DO and temperature at each depth separately in simple models (e.g. Below55P ~ s(DO300)), to determine the most important depths for DO and temperature, which here are DO300 and Temp300.

Table S29: Models to determine the most important depths for DO and temperature variables. The selected depths shown in **bold**

Model	df	AIC	wAIC	% Deviance
DO50	2.0057	2552.76	0.0034	0.00
**DO100**	**5.3236**	**2542.70**	**0.5197**	**5.90**
DO150	6.6407	2543.74	0.3091	1.72
DO200	4.1270	2551.77	0.0056	0.00
DO250	2.0024	2552.75	0.0034	0.00
**DO300**	**6.9504**	**2545.07**	**0.1588**	**5.67**
Temp50	6.2338	2547.42	0.0703	4.48
Temp100	4.1058	2552.35	0.0060	1.52
Temp150	6.0689	2548.15	0.0488	4.14
Temp200	2.9770	2548.45	0.0420	2.06
Temp250	2.9775	2548.44	0.0422	2.06
**Temp300**	**4.8424**	**2542.58**	**0.7907**	**5.13**

Having established that DO300 and Temp300 are the most important factors, we then tested models using combinations of those factors, together with SST, to determine the most important drivers of behaviour resulting in increased time below 55 m. We note here that while DO100 explains slightly more deviance, DO300 is also an important factor, and therefore models including both factors were tested.

Table S30: GAM outputs showing DO and SST explain the most deviance

Model	df	AIC	wAIC	% Deviance
s(DO100)	5.3236	2542.70	0.0000	5.90
s(DO300)	6.9504	2545.07	0.0000	5.67
s(Temp300)	4.8424	2542.58	0.0000	5.13
s(SST)	4.4946	2539.37	0.0000	5.92
s(DO100) + s(Temp300)	7.4729	2539.08	0.0000	7.85
s(DO100) + s(SST)	7.3686	2531.72	0.0000	10.00
s(Temp300) + s(SST)	7.0080	2531.76	0.0000	9.79
s(DO100) + s(Temp300) + s(SST)	8.7187	2531.80	0.0000	10.80
s(SST) + s(DO100) + s(DO300)	**12.9901**	**2511.72**	**0.6058**	**18.90**
s(DO100) + s(DO300) + s(Temp300) + s(SST)	14.9282	2512.58	0.3941	19.70

Including DO100 and DO300 increased deviance to 18.9 %, suggesting that time below 55 m is influenced by DO at both depths. Including SST in the model did increase deviance explained, but the additional parameter reduced the *wAIC*, making the simpler model the better choice.

```
Family: gaussian
Link function: identity

Formula:
```

```
Below55P ~ s(SST) + s(DO100) + s(DO300)

Parametric coefficients:
            Estimate Std. Error t value Pr(>|t|)
(Intercept)  47.2226     0.8816   53.56   <2e-16 ***
---
Signif. codes:  0 ‘***’ 0.001 ‘**’ 0.01 ‘*’ 0.05 ‘.’ 0.1 ‘ ’ 1

Approximate significance of smooth terms:
           edf Ref.df     F  p-value
s(SST)   1.979      9 2.030 2.49e-05 ***
s(DO100) 2.558      9 1.776 0.000197 ***
s(DO300) 4.720      9 2.897 1.91e-05 ***
---
Signif. codes:  0 ‘***’ 0.001 ‘**’ 0.01 ‘*’ 0.05 ‘.’ 0.1 ‘ ’ 1

R-sq.(adj) =  0.163   Deviance explained = 18.9%
-REML = 1259.9  Scale est. = 233.94    n = 301
```

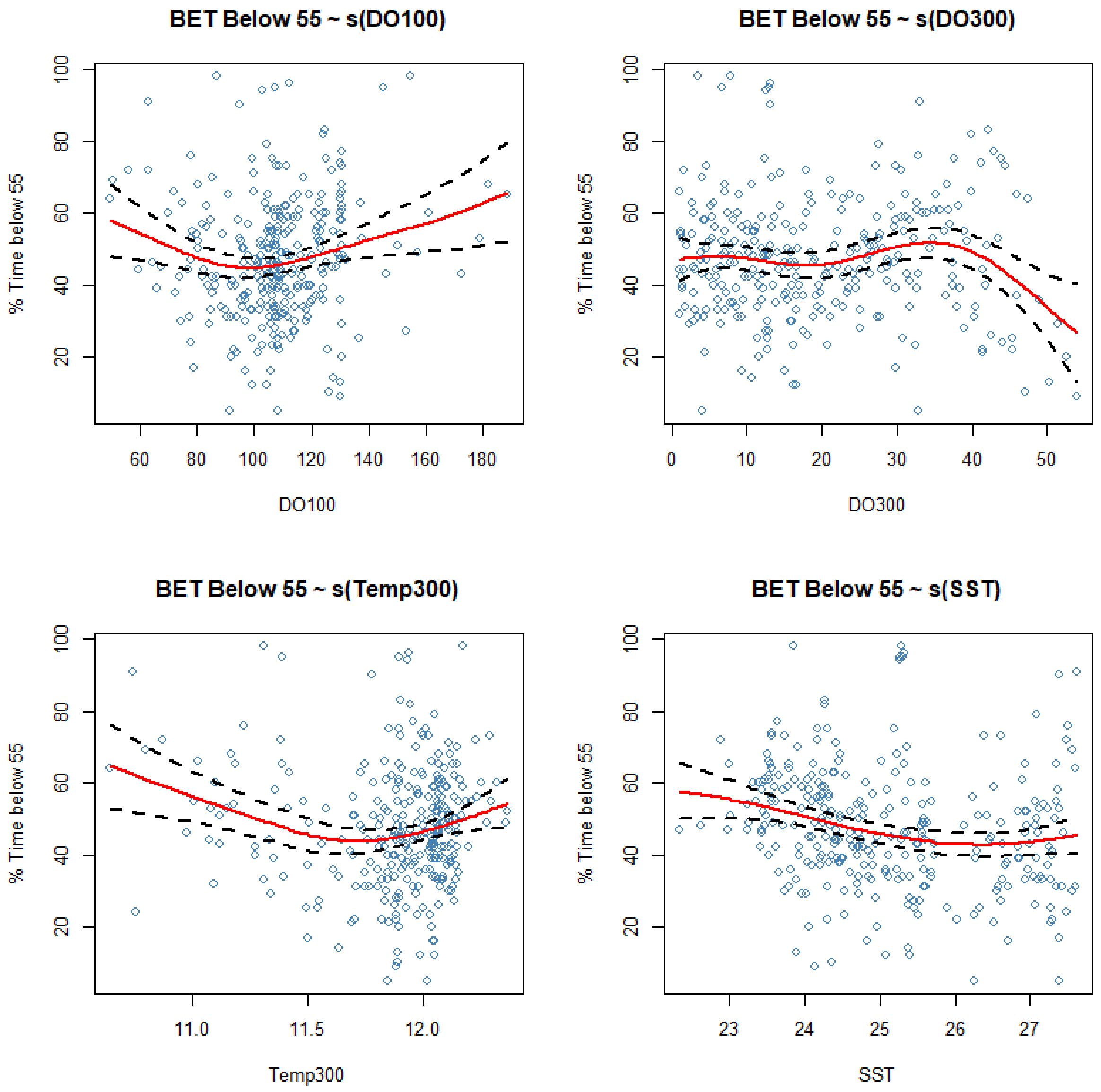

Figure S23: Plots of the variables explaining the most deviance for BET time below 55 m

The model selected (DO100, DO300 and Temp300) explains 18.9 % of the deviance. While adding SST increases deviance explained to 19.7 %, it reduces the wAIC value. The relationship between time below 55 m and DO at 300 m is unexpected, however, with increasing DO resulting in reduced time below 55 m. None of the factors make large differences to behaviour. Blue circles are observations; red line is best fit; dashed lines are 95% confidence intervals.

BET Maximum depth

For the detailed analysis of time below the behavioural threshold of 55 m and the analysis of maximum depths, time series records with depths > 500 m were omitted, as these were considered to represent behaviour outside of normal daily activity; for example, sporadic deep dives to below 1 km. However, for the spatial analysis of maximum and average depths, all depth records were included, to reduce a bias towards the chosen maximum depth limit, which would otherwise be selected as the maximum depth in the majority of grid cells. To explain further, in many grid cells, deep dives equal to the specified threshold would be observed at least once, resulting in the maximum depth consistently being biased to whatever threshold was set. For both average and maximum depths however, a few extreme outliers were removed (e.g. depths of > 3000 m).

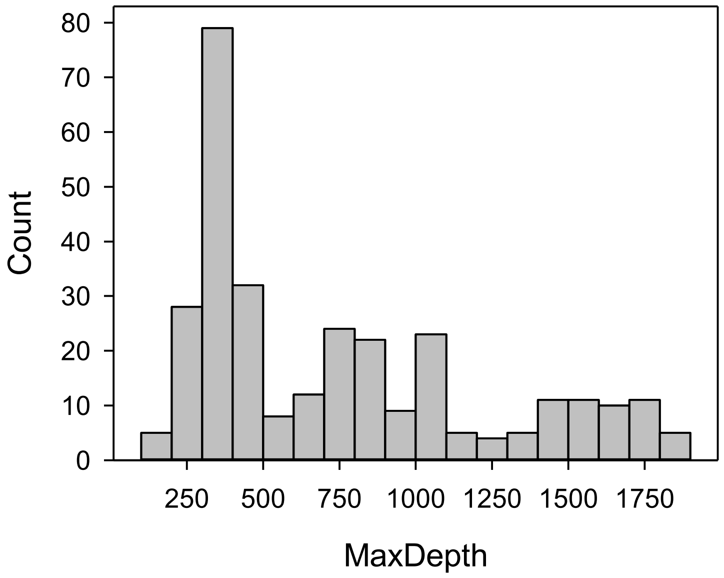

Figure S24: Histogram showing the distribution of maximum daily depth counts for BET

Again, using a simple model, we first determine the best fit distribution for the data, which in this case is inverse Gaussian (link = 1/mu^2). Note Gamma with link=identity produced errors.

Table S31: Models to determine the best fit distribution for BET maximum daily depth counts
The best fit model shown in **bold**

Distribution	df	AIC	wAIC
Gaussian	5.2308	4552.65	0.0000
Gaussian (link = log)	5.4824	4552.41	0.0000
**Inverse Gaussian (link = 1/mu^2)**	**5.8943**	**4410.29**	**1.0000**
Gamma (link = log)	5.9733	4439.14	0.0000
Gamma (link=identity)	5.2183	4437.28	0.0000
Poisson	9.9581	79279.81	0.0000

Using this distribution, we test to determine the most important depths for DO and temperature, which here are DO150 and Temp200. It is interesting that although Temp50 does explain the most deviance (19.6 %), temperatures at all depths also explain at least 17 % of deviance, suggesting that temperature generally is an important factor in maximum daily depth. For DO as well, while DO50 is the most important factor, DO100 also explains 16.6 % of deviance.

Table S32: Models to determine the most important depths for DO and temperature variables.
The selected depths shown in **bold**

Model	df	AIC	wAIC	% Deviance
DO50	7.6023	4400.55	0.0000	11.6
DO100	8.7302	4385.47	0.0023	16.6
**DO150**	**8.1536**	**4373.32**	**0.9977**	**19.6**
DO200	7.5738	4402.43	0.0000	11.1
DO250	4.5529	4420.15	0.0000	3.77
DO300	5.8943	4410.29	0.0000	7.7
Temp50	6.7239	4347.68	0.1934	25.4
Temp100	8.1099	4381.42	0.0000	17.4
Temp150	6.7149	4351.06	0.0357	24.6
**Temp200**	**7.5069**	**4344.92**	**0.7687**	**26.5**
Temp250	7.6101	4356.77	0.0021	23.6
Temp300	7.1520	4362.12	0.0001	22.0

Table S33: GAM outputs showing DO and temperature explain the most deviance

Model	df	AIC	wAIC	% Deviance
s(DO150)	7.5911	4504.40	0.0000	21.7
s(Temp200)	8.0112	4476.06	0.0000	29.0
s(SST)	6.5319	4478.72	0.0000	27.6
s(DO150) + s(Temp200)	14.5606	4360.52	0.0000	53.7
s(DO150) + s(SST)	12.8634	4459.46	0.0000	34.9
s(Temp200) + s(SST)	10.6767	4362.12	0.0000	52.2
s(DO150) + s(Temp200) + s(SST)	**15.3883**	**4320.25**	**1.0000**	**59.7**

The model comprising DO150, Temp200 and SST explained significantly more deviance than the other models. This result differs somewhat from expectations; it was expected that DO and temperatures at the deeper depths, where both factors have low, possibly threshold values, would be most important.

The details of the best model output are shown below:

```
Family: gaussian
Link function: log

Formula:
MaxDepth ~ s(SST) + s(DO150) + s(Temp200)

Parametric coefficients:
            Estimate Std. Error t value Pr(>|t|)
(Intercept)  6.46103    0.03465   186.5   <2e-16 ***
---
Signif. codes:  0 ‘***’ 0.001 ‘**’ 0.01 ‘*’ 0.05 ‘.’ 0.1 ‘ ’ 1

Approximate significance of smooth terms:
              edf Ref.df      F  p-value
s(SST)     0.9824      9  5.177 1.36e-11 ***
s(DO150)   5.3239      9  6.368 2.91e-11 ***
s(Temp200) 6.2327      9 17.793  < 2e-16 ***
---
Signif. codes:  0 ‘***’ 0.001 ‘**’ 0.01 ‘*’ 0.05 ‘.’ 0.1 ‘ ’ 1

R-sq.(adj) =  0.579   Deviance explained = 59.7%
-REML = 2176.6  Scale est. = 94741     n = 301
```

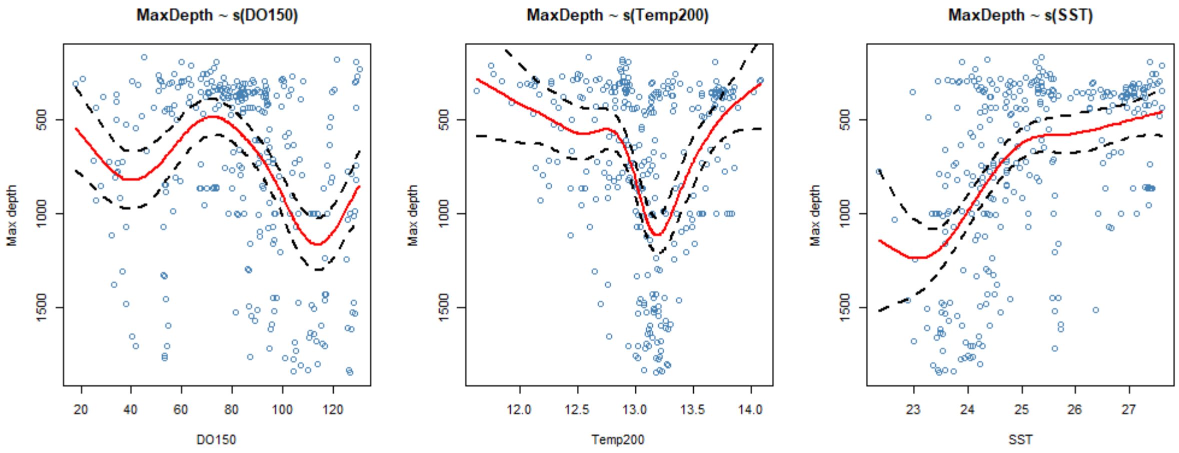

Figure S25: Plots of the two variables explaining most of the deviance.

All these terms are significant, and in combination explain 59.7 % of the deviance. However, the relationships are complex. Maximum depth appears to peak at DO concentrations of around 120 µmol/l, increasing temperature at 200 m produces a peak depth at around 13.25 C and increasing SST appears to reduce the maximum depth, contrary to expectations. Blue circles are observations; red line is best fit; dashed lines are 95% confidence intervals.

BET Average depth

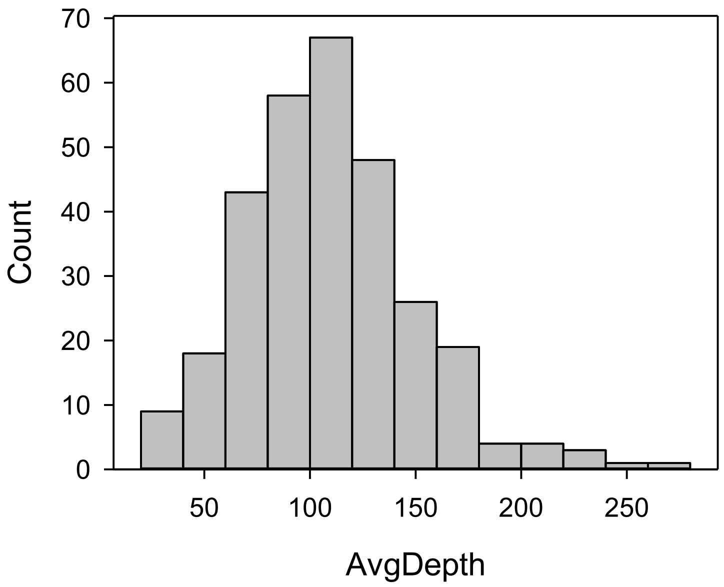

Figure S26: Histogram showing the distribution of average daily depth counts for BET

Again, using a simple model, we first determine the best fit distribution for the data, which in this case is Gaussian

Table S34: Models to determine the best fit distribution for BET average daily depth counts
The best fit model shown in **bold**

Distribution	df	AIC	wAIC
Gaussian	5.5720	3074.42	0.0000
Gaussian (link = log)	5.1313	3075.86	0.0000
Inverse Gaussian (link = 1/mu^2)	6.3222	3077.50	0.0000
Gamma (link = log)	6.5535	3053.26	0.0463
**Gamma (link=identity)**	**6.9783**	**3047.21**	**0.9537**
Poisson	9.4602	6032.17	0.0000

Using this distribution, we test to determine the most important depths for DO and temperature, which here are DO300 and Temp150. It is notable that temperature at 200 and 250 m both explain nearly as much deviance as that selected by AIC. DO at 300 m is clearly a more important factor than DO at other depths.

Table S35: Models to determine the most important depths for DO and temperature variables.
The selected depths shown in **bold**

Model	df	AIC	wAIC	% Deviance
DO50	3.1578	3061.18	0.0009	0.72
DO100	3.6586	3060.26	0.0014	1.34
DO150	4.5633	3056.79	0.0082	3.02
DO200	3.3415	3061.32	0.0009	0.80
DO250	2.0032	3061.11	0.0009	0.00
**DO300**	**6.9783**	**3047.21**	**0.9876**	**7.45**
Temp50	4.1494	3059.51	0.1368	1.90
Temp100	2.0031	3061.11	0.0614	**0.00**
**Temp150**	**4.6342**	**3057.07**	**0.4632**	**2.97**
Temp200	4.1526	3059.87	0.1142	1.78
Temp250	2.0024	3061.11	0.0614	0.00
Temp300	2.9470	3059.16	0.1629	1.24

Table S36: GAM outputs showing DO, temperature and SST explain the most deviance

Model	df	AIC	wAIC	% Deviance
s(DO300)	5.1313	3075.86	0.0000	3.13
s(Temp150)	4.7632	3074.88	0.0000	3.21
s(SST)	4.6090	3062.26	0.0016	7.09
s(DO300) + s(Temp150)	8.2125	3069.56	0.0000	7.06
s(DO300) + s(SST)	9.0245	3052.75	0.1898	12.6
s(Temp150) + s(SST)	6.7484	3060.88	0.0033	8.82
**s(DO300) + s(Temp150) + s(SST)**	**11.3459**	**3049.86**	**0.8052**	**14.7**

Here the model with DO at 300 m, temperature at 150 m and SST is favoured by AIC, explaining 14.7 % of the deviance.

```
Family: gaussian
Link function: log

Formula:
AvgDepth ~ s(SST) + s(DO300) + s(Temp150)

Parametric coefficients:
            Estimate Std. Error t value Pr(>|t|)
(Intercept)   4.6865     0.0201   233.2   <2e-16 ***
---
Signif. codes:  0 ‘***’ 0.001 ‘**’ 0.01 ‘*’ 0.05 ‘.’ 0.1 ‘ ’ 1

Approximate significance of smooth terms:
             edf Ref.df     F  p-value
s(SST)     2.030      9 2.603 1.81e-06 ***
s(DO300)   3.486      9 1.300  0.00714 **
s(Temp150) 1.886      9 0.594  0.03834 *
---
Signif. codes:  0 ‘***’ 0.001 ‘**’ 0.01 ‘*’ 0.05 ‘.’ 0.1 ‘ ’ 1

R-sq.(adj) =  0.126   Deviance explained = 14.7%
-REML = 1530.1  Scale est. = 1404.6    n = 301
```

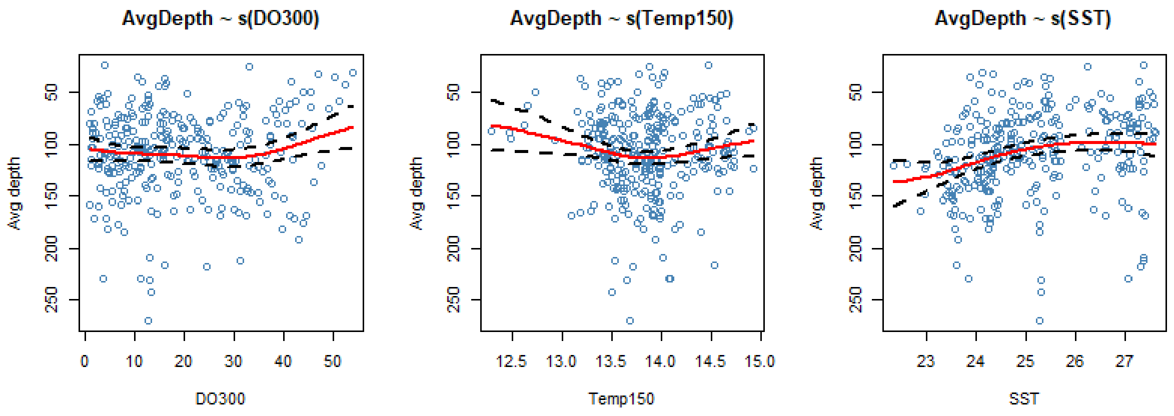

Figure S27: Plots of the variables explaining most of the deviance.
Blue circles are observations; red line is best fit; dashed lines are 95 % confidence intervals.

BET Vertical excursions

Two entries with zero excursions were dropped from the analysis to allow all distributions to be tested.

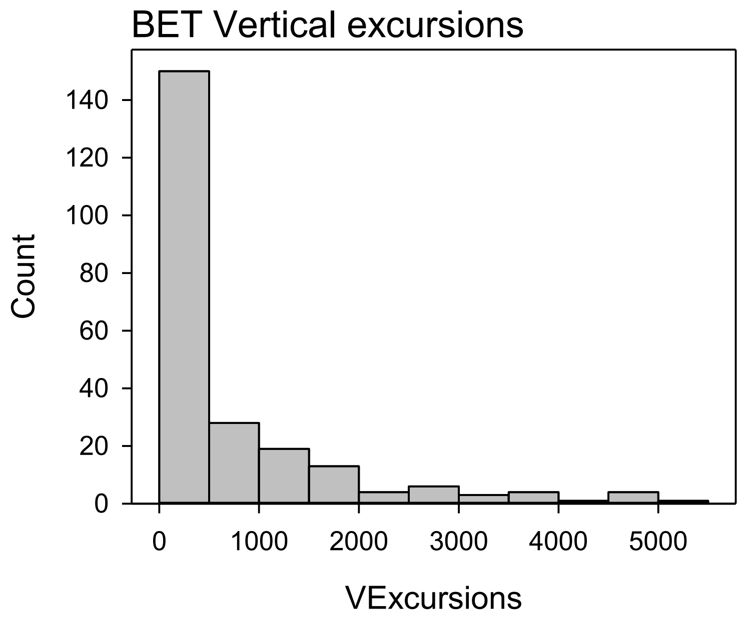

Figure S28: Histogram showing the distribution of daily vertical excursion counts for BET

Test to find the best distribution – note Gamma with link = identity gave warnings. Gamma is best here.

Table S37: Models to determine the best fit distribution for BET vertical excursion counts
The best fit model shown in **bold**, Gamma (link=identity) gave warnings

Distribution	df	AIC	wAIC
Gaussian	4.8760	5027.86	0.0000
Gaussian (link = log)	4.9013	5027.11	0.0000
Inverse Gaussian (link = 1/mu^2)	2.0040	4469.06	0.0000
**Gamma (link = inverse)**	**6.1708**	**4343.18**	**1.0000**
Poisson	10.0000	302342.44	0.0000

Using this distribution, we test to determine the most important depths for DO and temperature, which here are DO150 and Temp50, however temperatures at all depths explain more than 21 % of the deviance. Interestingly, it is DO at shallower depths that is more important. The expectation here was that it would be DO and temperature at depth that would be more important in the number of vertical excursions performed.

Table S38: Models to determine the most important depths for DO and temperature variables.
The selected depths shown in **bold**

Model	df	AIC	wAIC	% Deviance
DO50	7.3756	4301.64	0.0000	19.9
DO100	8.9240	4288.60	0.0000	23.2
**DO150**	**9.8779**	**4210.00**	**1.0000**	**38.1**
DO200	6.3193	4295.68	0.0000	20.7
DO250	5.7217	4337.47	0.0000	11.4
DO300	5.3908	4346.27	0.0000	9.18
**Temp50**	**8.2107**	**4186.96**	**1.0000**	**41.3**
Temp100	7.0976	4312.44	0.0000	17.5
Temp150	7.3978	4240.39	0.0000	31.9
Temp200	7.7595	4207.46	0.0000	37.8
Temp250	7.5303	4224.21	0.0000	34.8
Temp300	6.9360	4239.99	0.0000	31.8

Table S39: GAM outputs showing DO, SST and temperature explain the most deviance

Model	df	AIC	wAIC	% Deviance
s(DO150)	8.3570	4924.81	0.0000	38.1
s(Temp50)	6.5018	4929.09	0.0000	41.3
s(SST)	7.6602	4872.92	0.0000	45.3
s(DO150) + s(Temp50)	14.2216	4753.55	0.0000	64.8
s(DO150) + s(SST)	11.6765	4824.52	0.0000	54.7
s(Temp50) + s(SST)	12.1735	4777.86	0.0000	61.3
**s(DO150) + s(Temp50) + s(SST)**	**14.9881**	**4690.85**	**1.0000**	**71.6**

In this case DO150, Temp50 and SST were selected by AIC, although several models explain more than 50% of the deviance and DO150 itself explains 38%. It is possible that when surface waters are warmer, BET perform more vertical excursions to deeper water as they are better able to warm quickly between deeper dives.

```
Formula:
VExcursions ~ s(SST) + s(DO150) + s(Temp50)

Parametric coefficients:
            Estimate Std. Error t value Pr(>|t|)
(Intercept)   4.8113     0.3322   14.48   <2e-16 ***
---
Signif. codes:  0 ‘***’ 0.001 ‘**’ 0.01 ‘*’ 0.05 ‘.’ 0.1 ‘ ’ 1

Approximate significance of smooth terms:
            edf Ref.df      F  p-value
s(SST)    1.186      9  6.451 1.89e-14 ***
s(DO150)  5.478      9 11.353  < 2e-16 ***
s(Temp50) 4.603      9 12.912  < 2e-16 ***
---
Signif. codes:  0 ‘***’ 0.001 ‘**’ 0.01 ‘*’ 0.05 ‘.’ 0.1 ‘ ’ 1

R-sq.(adj) =  0.706   Deviance explained = 71.6%
-REML = 2360.5  Scale est. = 3.2395e+05  n = 301
```

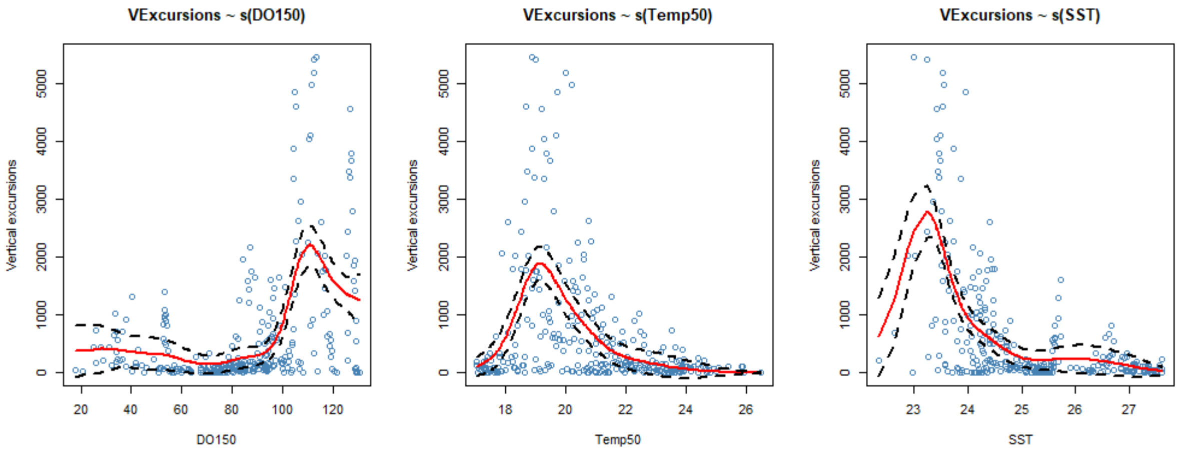

Figure S29: Variables explaining the most deviance for BET vertical excursions

The number of vertical excursions is greater at higher levels of DO, at low temperatures at 50 m and at higher SSTs, which is counter to expectations. It is likely that SST and Temp50 exhibit concurvity and vary in a similar way, despite the effect seeming to differ. Blue circles are observations; red line is best fit; dashed lines are 95 % confidence intervals.

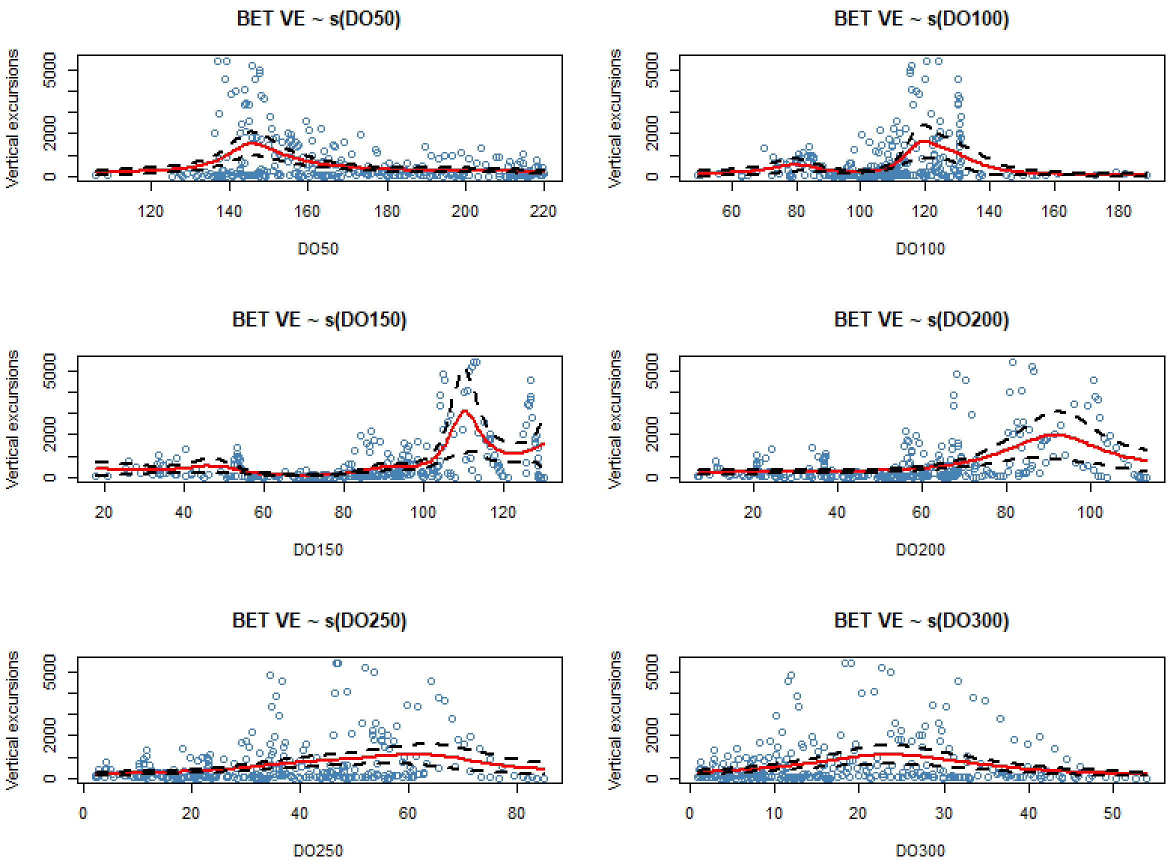

Figure S30 Plots of the GAM results for vertical excursions with DO at all depths

At no depth is there a clear relationship between DO and the number of vertical excursions performed.

GAM Analysis of YFT Spatial data

Time below 43 m

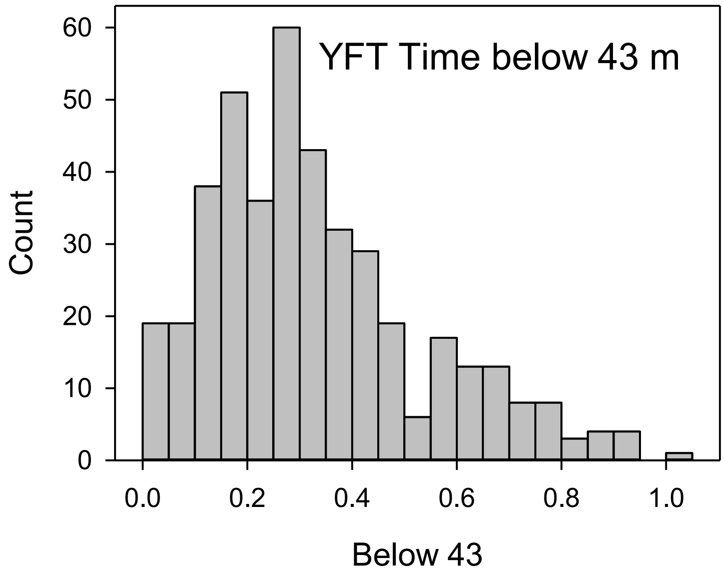

Figure S31: Histogram showing the distribution of time below 43 m proportions for YFT

The best fit distribution was found to be Gaussian with link=log.

Table S40: Models to determine the best fit distribution for YFT time below 43 m proportions
The best fit model shown in **bold**

Distribution	df	AIC	wAIC
Gamma (link = log)	6.360914	3658.196	0.0000
Gamma (link=inverse)	6.058294	3658.286	0.0000
Gaussian	4.105242	3886.661	0.0000
**Gaussian (link=log)**	**5.40120**	**3614.797**	**0.5366**
Inverse Gaussian (link = 1/mu^2)	6.503984	3615.09	0.4634
Poisson	8.841424	6343.068	0.0000

Using this distribution, we test to determine the most important depths for DO and temperature.

Table S41: Models to determine the most important depths for DO and temperature variables. The selected depths shown in **bold**

Model	df	AIC	wAIC	% Deviance
DO50	8.911423	3697.005	0.0000	15.7
DO100	6.058294	3658.286	0.0000	22.1
**DO150**	**6.097179**	**3584.005**	**0.9993**	**34.6**
DO200	6.579056	3598.414	0.0007	32.5
DO250	6.036823	3614.485	0.0000	29.7
DO300	5.608129	3622.497	0.0000	28.2
Temp50	8.26138	3685.601	0.0000	17.7
Temp100	8.53824	3589.81	0.0000	34.5
**Temp150**	**8.659674**	**3553.783**	**1.0000**	**39.9**
Temp200	8.377184	3689.288	0.0000	17.1
Temp250	8.666046	3638.328	0.0000	26.6
Temp300	8.428535	3668.118	0.0000	21.1

Using these results we tested whether SST, DO at 150 m or temperature at 150 m was a more important driver of the number of vertical excursions (i.e. explained a greater % of the deviance).

Table S42: GAM outputs showing temperature and SST explain the most deviance

Model	df	AIC	wAIC	% Deviance
s(DO150)	6.097179	3584.005	0.0000	34.6
s(Temp150)	8.659674	3553.783	0.0000	39.9
s(SST)	7.551848	3676.089	0.0000	19.3
s(DO150) + s(Temp150)	9.139362	3537.319	0.0000	42.3
s(DO150) + s(SST)	10.9132	3556.174	0.0000	40.2
**s(Temp150) + s(SST)**	**16.59388**	**3490.168**	**0.5459**	**50.2**
s(DO150) + s(Temp150) + s(SST)	18.08969	3490.536	0.4541	50.5

Although the model with all variables explained slight more of the deviance, the model with temperature and SST was selected by AIC. This model explains 50.2 % of the deviance.

```
Family: gaussian
Link function: log

Formula:
Below43P ~ s(SST) + s(Temp150)

Parametric coefficients:
            Estimate Std. Error t value Pr(>|t|)
(Intercept)    3.376      0.031   108.9   <2e-16 ***

Approximate significance of smooth terms:
             edf Ref.df     F p-value
s(SST)     6.295      9 10.61  <2e-16 ***
```

```
s(Temp150) 6.294      9 21.23  <2e-16 ***

R-sq.(adj) =  0.486   Deviance explained = 50.2 %
-REML = 1761.9  Scale est. = 214.28    n = 423
```

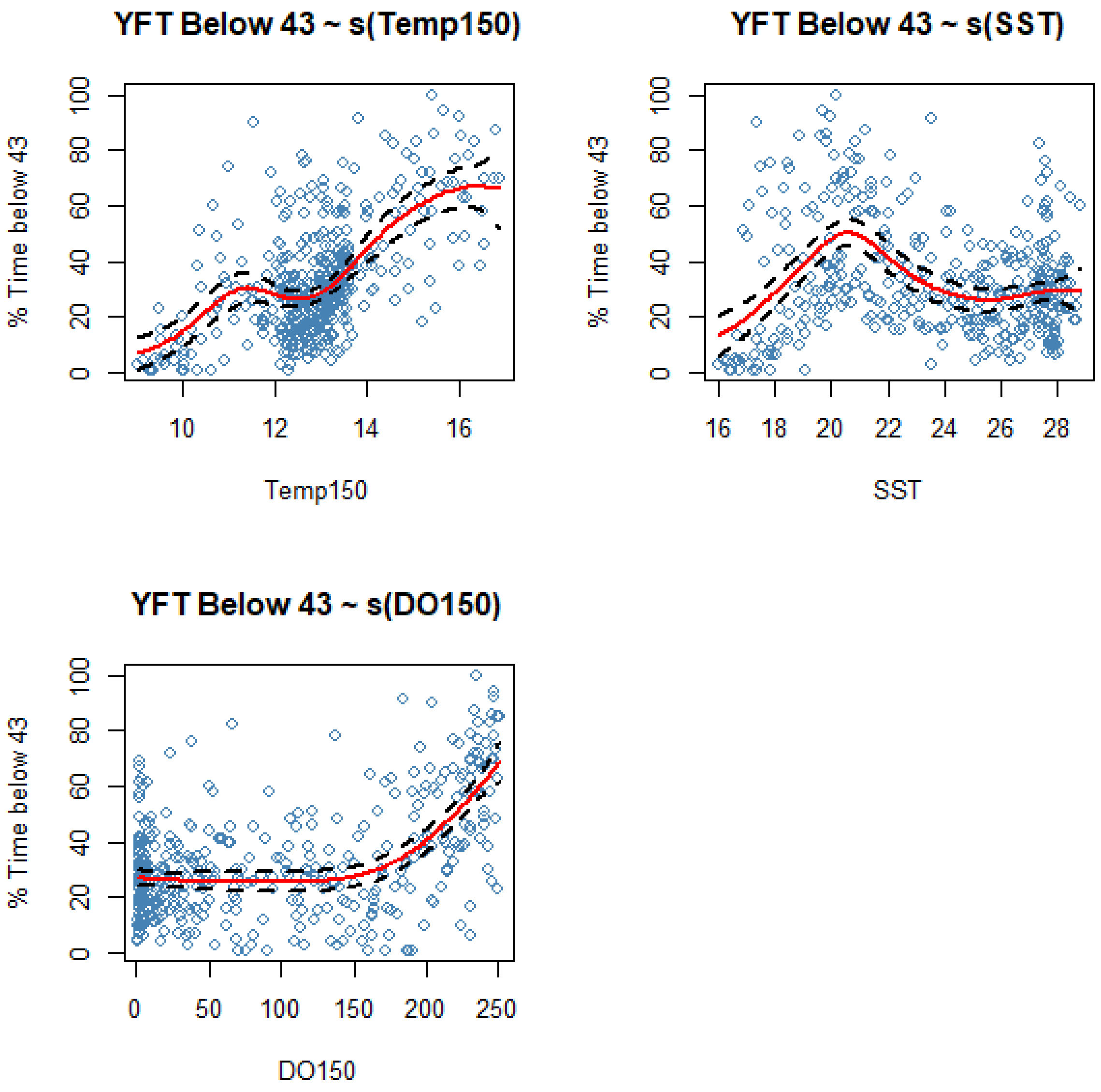

Figure S32: Plots of the effect of SST and temperature on YFT time below 43 m

Time below 43 m increases with increasing temperatures at 150 m. The effect of SST is more complex, resulting in less time below 43 m in lower temperatures, with a peak at around 22 °C, which then levels off. Time below 43 m shows a clear increase with increasing DO, once concentration exceeds 150 µmol/l. Blue circles are observations; red line is best fit; dashed lines are 95 % confidence intervals.

YFT Maximum daily depth

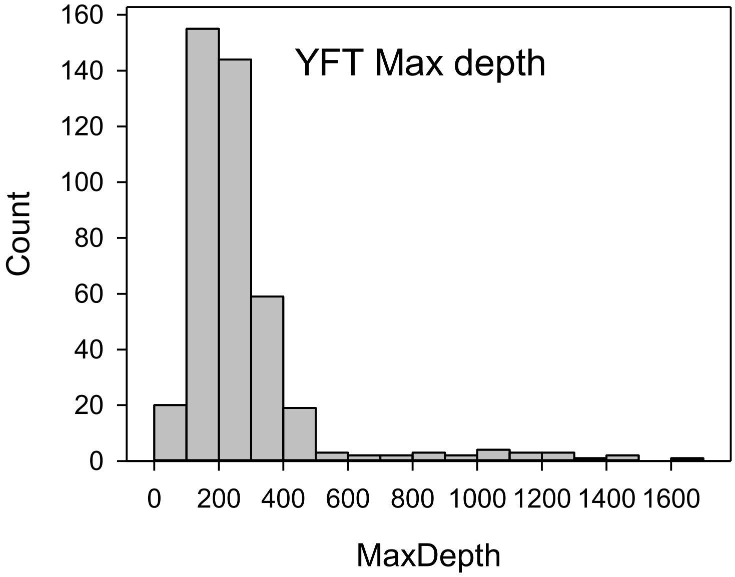

Figure S33: Histogram showing the distribution of maximum daily depth counts for YFT

Table S43: Models to determine the best fit distribution for YFT maximum depth counts
The best fit model shown in **bold**

Distribution	df	AIC	wAIC
Gaussian	7.5426	5689.40	0.0000
Gaussian (log)	8.1239	5689.64	0.0000
**Inverse Gaussian**	**8.0883**	**5204.80**	**1.0000**
Gamma (link = "inverse")	8.1283	5272.16	0.0000
Gamma (link = identity)	7.9980	5273.42	0.0000
Poisson	8.9872	40764.49	0.0000

Using this distribution, we test to determine the most important depths for DO and temperature.

Table S44: Models to determine the most important depths for DO and temperature variables. The selected depths shown in **bold**

Model	df	AIC	wAIC	% Deviance
DO50	3.0018	5294.33	0.0000	4.44
**DO100**	**8.0883**	**5204.80**	**1.0000**	**24.50**
DO150	7.6469	5243.32	0.0000	17.10
DO200	5.7927	5279.98	0.0000	8.84
DO250	4.7934	5290.48	0.0000	6.10
DO300	3.9236	5293.90	0.0000	4.95
Temp50	3.7976	5285.62	0.0051	1.45
**Temp100**	4.3716	5275.10	0.9743	4.14
Temp150	3.8121	5285.97	0.0042	1.38
Temp200	2.6501	5288.47	0.0012	0.24
Temp250	2.7500	5288.26	0.0014	0.34
Temp300	4.7738	5283.61	0.0138	2.38

Table S45: GAM outputs showing DO and SST explain the most deviance

Model	df	AIC	wAIC	% Deviance
s(DO100)	8.0883	5204.80	0.0001	24.50
s(Temp100)	4.6975	5299.28	0.0000	4.09
s(SST)	5.0695	5295.54	0.0000	5.10
s(DO100) + s(Temp100)	8.1058	5204.83	0.0001	24.50
**s(DO100) + s(SST)**	**11.2656**	**5188.79**	**0.4398**	**28.40**
s(Temp100) + s(SST)	7.3500	5272.80	0.0000	11.00
**s(DO100) + s(Temp100) + s(SST)**	**11.02791**	**5188.307**	**0.5599**	**28.4**

Here DO temperature and SST are selected as the best model by AIC, explaining 28.4 % of the deviance in maximum depth. However, the model output shows SST to not be significant (p =0.627), and therefore the model with DO100 and SST, which also explains 28.4 % deviance, should be selected.

```
Family: inverse.gaussian
Link function: 1/mu^2

Formula:
MaxDepth ~ s(SST) + s(DO100)

Parametric coefficients:
             Estimate Std. Error t value Pr(>|t|)
(Intercept) 1.717e-05  1.084e-06   15.84   <2e-16 ***

Approximate significance of smooth terms:
           edf Ref.df     F p-value
s(SST)   2.575      9 1.314  0.0026 **
s(DO100) 5.849      9 8.362 2.8e-15 ***

R-sq.(adj) =  0.213   Deviance explained = 28.4 %
```

```
-REML = 2614.7  Scale est. = 0.0016763  n = 423
```

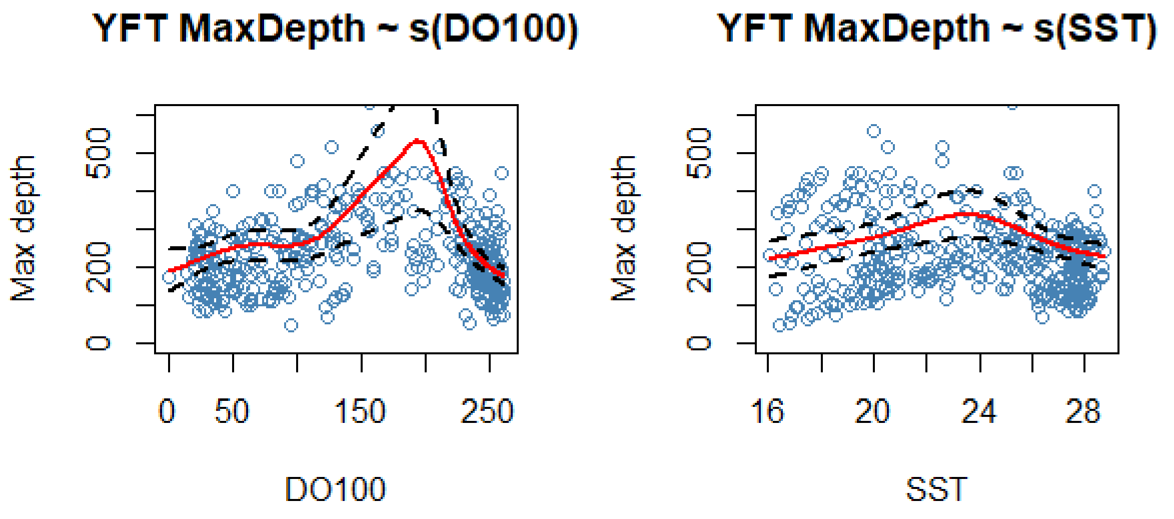

Figure S34: Plots of the effect of DO at 100 m and SST on YFT maximum depth

Depths > 600 m omitted for clarity. Maximum depth increases with increasing DO up to about 200 µmol/l, suggesting that DO at 100 m acts to reduce water column use. SST has a lesser effect, with a peak in maximum depth around 24 °C. Blue circles are observations; red line is best fit; dashed lines are 95 % confidence intervals.

YFT Average depth

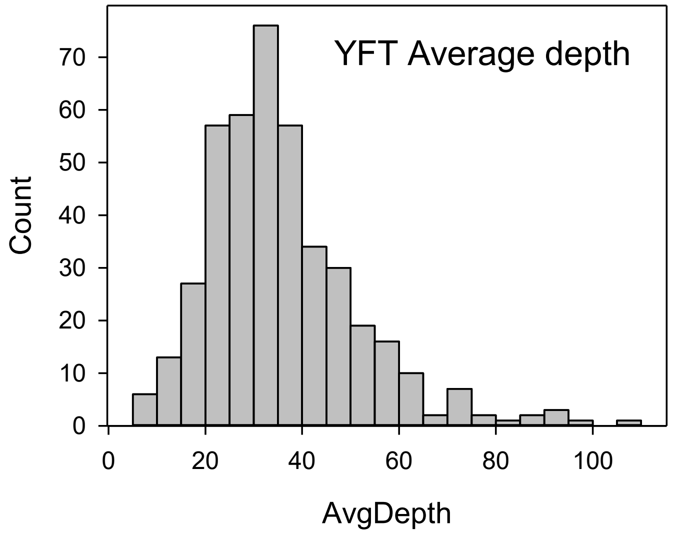

Figure S35: Histogram showing the distribution of average daily depth counts for YFT

Table S46: Models to determine the best fit distribution for YFT average depth counts

The best fit model shown in **bold**

Distribution	df	AIC	wAIC
Gaussian	5.3397	3439.78	0.0000
Gaussian (log)	5.1283	3439.32	0.0000
Inverse Gaussian (link = 1/mu^2)	4.5809	3386.60	0.0000
**Gamma (link = "inverse")**	**4.9506**	**3357.06**	**0.6130**
Gamma (link = identity)	5.3977	3357.98	0.3870
Poisson	8.7870	4410.49	0.0000

Using this distribution, we test to determine the most important depths for DO and temperature.

Table S47: Models to determine the most important depths for DO and temperature variables.

The selected depths shown in **bold**

Model	df	AIC	wAIC	% Deviance
DO50	9.2301	3364.166	0.0000	20.2
DO100	4.9291	3371.255	0.0000	17.3
**DO150**	**6.5245**	**3318.553**	**0.9958**	**27.3**
DO200	6.8802	3329.472	0.0042	25.6
DO250	6.3410	3344.132	0.0000	22.8
DO300	5.2970	3353.263	0.0000	20.8
Temp50	8.9330	3337.79	0.0000	22.20
Temp100	8.4121	3239.04	0.0000	38.00
**Temp150**	8.9485	3206.70	1.0000	42.70
Temp200	8.2569	3351.12	0.0000	19.50
Temp250	8.1383	3312.07	0.0000	26.50
Temp300	8.2357	3341.98	0.0000	21.20

Table S48: GAM outputs showing DO, temperature and SST explain the most deviance

Model	df	AIC	wAIC	% Deviance
s(DO150)	6.5225	3304.18	0.0000	27.30
s(Temp150)	8.9485	3206.70	0.0248	42.70
s(SST)	7.7564	3338.82	0.0000	21.60
s(DO150) + s(Temp150)	9.7212	3202.33	0.2206	43.50
s(DO150) + s(SST)	11.6089	3235.93	0.0000	39.40
**s(Temp150) + s(SST)**	**11.2946**	**3200.98**	**0.4334**	**44.10**
s(DO150) + s(Temp150) + s(SST)	11.4820	3201.58	0.3212	44.00

```
Family: Gamma
Link function: inverse
```

```
Formula:
AvgDepth ~ s(SST) + s(Temp150)

Parametric coefficients:
             Estimate Std. Error t value Pr(>|t|)
(Intercept) 0.0304536  0.0004865    62.6   <2e-16 ***

Approximate significance of smooth terms:
             edf Ref.df      F  p-value
s(SST)     1.936      9  1.312 0.000918 ***
s(Temp150) 5.680      9 27.383  < 2e-16 ***

R-sq.(adj) =  0.476   Deviance explained = 44.1 %
-REML = 1612.2  Scale est. = 0.10129    n = 421
```

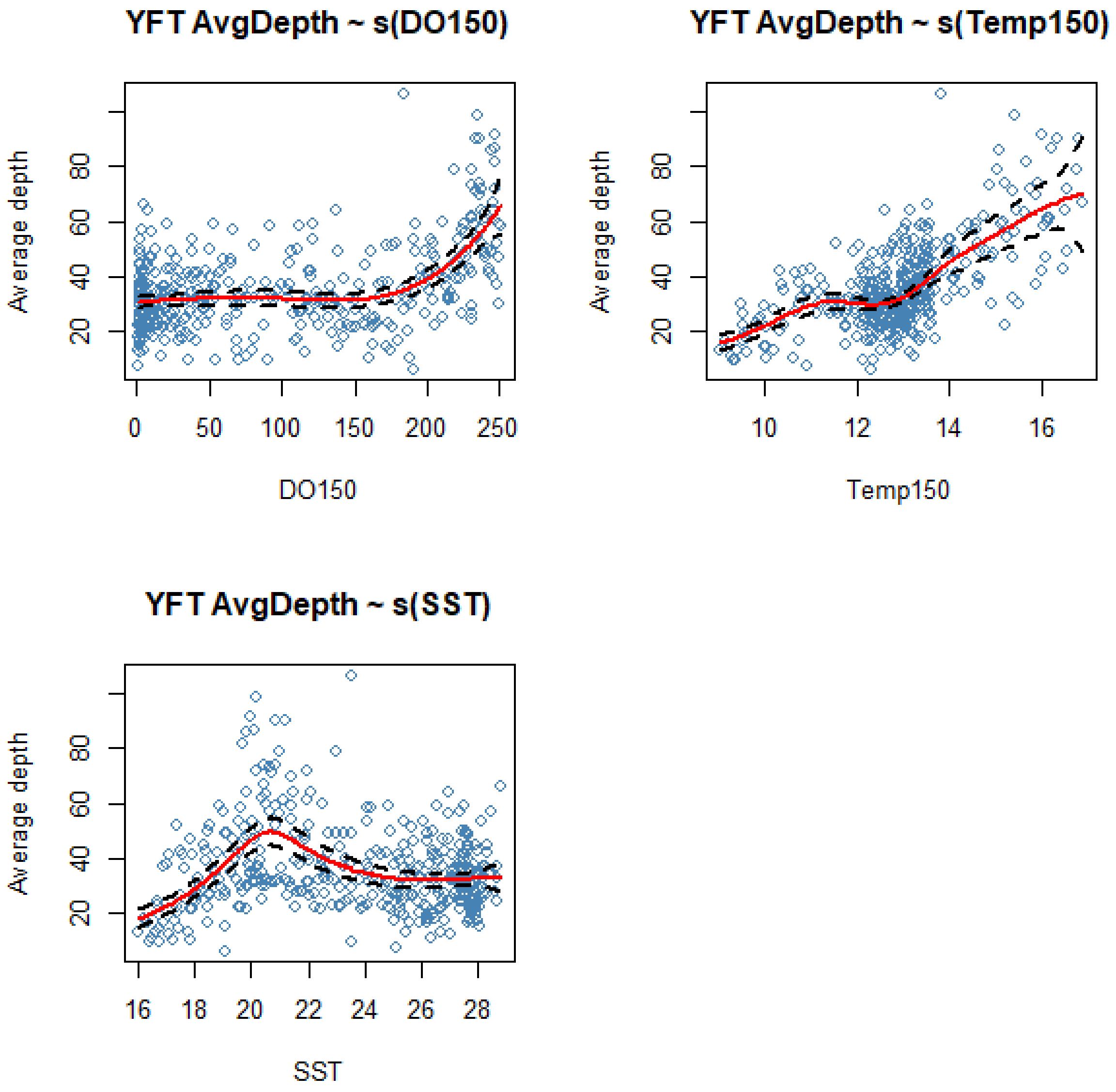

Figure S36: Plots of the effect of DO at 500 m, temperature at 150 m and SST on YFT average depth
With DO, average depth increases sharply once concentrations exceed ~175 µmol/l. Average depth increases with increasing temperature, suggesting that lower temperatures at 150 m act to reduce water column use. SST has a lesser effect, with a peak in average depth around 22 °C. Blue circles are observations; red line is best fit; dashed lines are 95 % confidence intervals.
